# Aberrant activation of the CD45-Wnt signaling axis promotes stemness and therapy resistance in colorectal cancer cells

**DOI:** 10.7150/thno.63446

**Published:** 2021-08-11

**Authors:** So-Yeon Park, Ji-Young Kim, Gyu-Beom Jang, Jang-Hyun Choi, Jee-Heun Kim, Choong-Jae Lee, Sunjae Lee, Jeong-Heum Baek, Kwan-Kyu Park, Jin-Man Kim, Hee Jin Chang, Nam-Chul Cho, Jeong-Seok Nam

**Affiliations:** 1 School of Life Sciences, Gwangju Institute of Science and Technology, Gwangju 61005, Republic of Korea.; 2 Cell Logistics Research Center, Gwangju Institute of Science and Technology, Gwangju 61005, Republic of Korea.; 3 Division of Colon and Rectal Surgery, Department of Surgery, Gil Medical Center, Gachon University College of Medicine, Incheon 21999, Republic of Korea.; 4 Department of Pathology, College of Medicine, Catholic University of Daegu, College of Medicine, Daegu 38430, Republic of Korea.; 5 Department of Pathology, College of Medicine, Chungnam National University, Daejeon 34134, Republic of Korea.; 6 Research Institute and Hospital, National Cancer Center, Goyang 10408, Republic of Korea.; 7 Drug Information Platform Center, Korea Research Institute of Chemical Technology, Daejeon 34114, Republic of Korea.

**Keywords:** Chemoradiation, Colorectal cancer, Therapy resistance, CD45, Cancer stem-like cell, Wnt, β-Catenin

## Abstract

**Rationale:** Chemoradiation (CRT) is commonly used as an adjuvant or neoadjuvant treatment for colorectal cancer (CRC) patients. However, resistant cells manage to survive and propagate after CRT, increasing the risk of recurrence. Thus, better understanding the mechanism of resistant cancer cells is required to achieve better clinical outcomes.

**Methods:** Here, we explored gene expression profiling of CRC patient tumors to identify therapy resistance genes and discovered that protein tyrosine phosphatase receptor type C (*PTPRC*), which encodes CD45, was increased in remnant tumor tissues after CRT and correlated with metastasis. Through multiple validations using patient tumors and CRC cell lines, we found for the first time the increase of CD45 expression in CRC (EpCAM+) epithelial cells surviving after CRT. Thus, we investigated the biological role and downstream events of CD45 were explored in human CRC cells and CRC mouse models.

**Results:** Increased CD45 expression in cancer cells in pretreated primary tumors accounts for poor regression and recurrence-free survival in CRT-treated patients. High CD45 expression promotes CRC cell survival upon 5-fluorouracil or radiation treatment, while CD45 depletion sensitizes CRC cells to CRT. Intriguingly, CD45 is preferentially expressed in cancer stem-like cells (CSCs), as determined by spheroid culture and the expression of CSC markers, and is required for the distinct functions of CSCs, such as cancer initiation, repopulation, and metastasis. Mechanistically, CD45 phosphatase activity promotes Wnt transcriptional activity by stabilizing the β-catenin protein, which collectively enhances stemness and the therapy-resistant phenotype.

**Conclusions:** Our results highlight a novel function of CD45 as a mediator of CRT resistance and provide a potential therapy strategy for CRC therapy.

## Introduction

Colorectal cancer (CRC) is a common malignant neoplasm and a major cause of cancer-related death worldwide 1. Complete surgical resection is the primary method of CRC treatment, and chemoradiation therapy (CRT) is a standard option for adjuvant and neoadjuvant treatment. However, tumors consist of subpopulations with differing CRT sensitivities, and cells with intrinsic resistance can survive CRT and continue to propagate 2. Therefore, treatment modalities targeting resistant cancer cells are needed to achieve better clinical outcomes.

Tumors consist of genetically and epigenetically divergent cell populations, including both cancer stem-like cells (CSCs) and differentiated cells 2. CSCs have the capacity to self-renew and repopulate the entire heterogeneous tumor cell population, thus accounting for tumor initiation, progression, and metastasis. Additionally, CSCs have distinct properties of CRT resistance 3. While CRT is cytotoxic to most tumor cells, it sometimes fails to eliminate CSCs 4, 5. Based on experimental evidence, tumors with a higher CSC density have a poorer response to CRT than those with a lower CSC density, confirming the contribution of CSCs to intrinsic resistance 6-10. Furthermore, non-CSCs may acquire CSC properties during therapy due to adaptive responses to microenvironmental changes, which is referred to as extrinsic resistance 11-13. These functional characteristics of CSCs allow them to survive during cancer therapy and cause local or distant recurrence; therefore, all CSCs must be inactivated or eliminated to achieve a permanent cure.

Strategies that block the signaling pathways underlying CSC resistance are under consideration as combination therapeutics, and some of these strategies have shown clinical advantages 14. Notably, CSC-related signaling pathways, including the Hedgehog, Wnt, and Notch signaling pathways, are attracting interest as therapeutic targets for CSCs 4. However, these signaling pathways also participate in the normal processes of development, homeostasis, and repair; therefore, the CSC-specific mechanism responsible for therapy resistance must be identified.

In this study, we performed gene expression profiling of tumors from CRC patients to identify therapy resistance genes and discovered that the expression of protein tyrosine phosphatase receptor type C (*PTPRC*), which encodes cluster of differentiation 45 (CD45), was increased in remnant tumor tissues after CRT and correlated with metastasis. Through multiple validations using patient tumors, we documented for the first time the existence of CD45-expressing CRC epithelial cells within patients' primary tumors, and investigated the biological role and downstream mechanism of CD45 in CRC epithelial cells.

## Methods

### Ethical approval and consent to participate

The retrospective study of patients with CRC (#20170410-BR-28-03-02) and immunohistological assessment of patient tissues (#20181106-BR-40-04-04) were preapproved by the Institutional Review Board (IRB) of Gwangju Institute of Science and Technology (GIST). Isolation of primary CRC cells from patient tissues was preapproved by the IRB of Gachon University School of Medicine (GCIRB-2013-66). All work related to human tissues was conducted in accordance with the Declaration of Helsinki. Written informed consent forms were signed and obtained from all subjects before participation. The gene expression profiles of therapy-resistant cancer were obtained using the “GEO2R” bioinformatics tool (available at http://www.ncbi.nlm.nih.gov/geo/geo2r/) and assessed using the Gene Expression Omnibus (GEO) webserver. The list of differentially expressed genes (DEGs) is attached in Table S1. Clinical information for the patient samples is described in Table S2. All animal experimental procedures were conducted according to protocols preapproved by the Institutional Animal Care and Use Committee (IACUC) of GIST (GIST-2017-038).

### Identification of gene expression signatures from public genomic databases

The gene expression profiles of therapy-resistant cancer were obtained using the “GEO2R” bioinformatics tool (available at http://www.ncbi.nlm.nih.gov/geo/geo2r/) and assessed using the Gene Expression Omnibus (GEO) web server. In patients with rectal cancer who underwent preoperative CRT (GSE93375), differentially expressed genes (DEGs) were obtained by comparing the residual CRC tissues after CRT (n = 9) with the pretreated tissues (n = 13, |fold change|≥2, *p*-value <0.05). The National Cancer Institute Colon Cancer Study (GSE68468) was obtained from the Georgetown Database of Cancer, a web platform that enables clinical research by integrating patient characteristics and clinical outcome data (https://gdoc.georgeto wn.edu/gdoc/), to identify the gene expression signature of metastatic cancer. DEGs were acquired by comparing nonmetastatic colon cancer (n = 183) and metastatic CRC in the liver or lung (n = 58, |fold change|≥1.5, *p*-value< 0.0005). DEGs in therapy-resistant cancer were compared with those in metastatic cancer, and commonly upregulated or downregulated genes in both cohorts were selected for further analysis. The DEG list is attached in Table S1. We used Ingenuity Pathway Analysis (IPA, Ingenuity System, Redwood City, CA, USA) to identify the potential upstream regulators that are significantly associated with DEGs. The STRING v10: functional protein association networks (https://string-db.org/) database was used to search for protein-protein interaction information.

### Isolation of primary CRC cells from patient tumors, fluorescence activated cell sorting (FACS) analysis, and validation

Primary tumors of CRC patients were minced completely until nearly liquid using scalpels and incubated with 0.1% collagenase type IV (Sigma-Aldrich, St. Louis, MO, USA) for 30 min. Contaminating red blood cells were lysed with RBC lysis buffer (Sigma-Aldrich). Live cells were gated with propidium iodide (PI) staining, and cells were stained with anti-epithelial cell adhesion molecule (EpCAM)-Alexa488 (eBioscience, San Diego, CA, USA) and anti-CD45-APC (BD Biosciences, San Diego, CA, USA) antibodies. EpCAM^+^ epithelial cells from CRC tissues were isolated using magnetic beads (Miltenyi Biotech, Auburn, CA, USA) and considered CRC cells. An immunohistological analysis was applied to validate CRC cells from primary cell mixtures based on EpCAM (Cell Signaling Technology, Beverly, MA, USA), CK-7 (Dako), and vimentin (BD Biosciences). Target proteins were visualized with Alexa488-conjugated secondary antibodies (Invitrogen, Carlsbad, CA, USA).

### Cell culture

Human CRC cell lines, including HCT116 (a colon carcinoma cell line), HT29 (a colon adenocarcinoma cell line), SW480 (a colon adenocarcinoma cell line), and DLD1 (a colon adenocarcinoma cell line), and the normal colon cell line CCD-18Co were purchased from the Korean Cell Line Bank (Seoul, Republic of Korea) in 2017-2019. Human CRC cells were cultured in Roswell Park Memorial Institute (RPMI) 1640 medium (Welgene, Daegu, Republic of Korea) supplemented with 5% fetal bovine serum (FBS) and 100 U/mL penicillin/streptomycin (Welgene) at 37 °C in humidified incubator with 5% CO_2_. Patient-derived primary CRC cells and CCD-18Co cells were cultured in Dulbecco's modified Eagle's Minimal Essential Medium (DMEM) (Welgene) supplemented with 10% FBS. Human lung fibroblast cells (MRC5) were a kind gift from Prof. Woo Keun Song (GIST, Gwangju, Republic of Korea) and cultured in Eagle's Minimum Essential Medium (MEM; Welgene) supplemented with 10% FBS. Irradiation was performed using a soft X-ray irradiator (Model M-100, SOFTEX, Tokyo, Japan). All experiments were performed with cells between passages 2-8. All cell lines were routinely tested for mycoplasma contamination every 6 months using the e-Myco^TM^ Mycopasma PCR detection kit (iNtron Biotechnology, Seongnam, Republic of Korea).

### Animal models for the *in vivo* limiting dilution assay and assessment of liver metastasis

For the comparison of tumor-initiating potential between CD45^high^ and CD45^low^ cells, patient-derived primary CRC cells or HCT116 cells were sorted into two groups according to the CD45 expression level using FACS (CD45^high^ and CD45^low^), and the cells were then subcutaneously inoculated into NSG mice (NOD.Cg-Prkdc^scid^ Il2rg^tm1Wjl^/SzJ, #005557, Jackson Laboratory, Bar Harbor, ME, USA) at various cell densities. After 28 days of observation, the frequency of tumor formation was monitored for 28 days and definitively determined by necropsy (n = 8 mice/group).

For the comparison of tumor-initiating potential between PTPRC knockdown and control HCT116 cells, cells were subcutaneously inoculated into NSG mice. On day 35, the mice were sacrificed, and the primary tumors were removed. Single tumor cells were isolated from the primary tumors by depleting mouse stromal cells with a mouse cell depletion kit (Miltenyl Biotec, Bergisch Gladbach, Germany), and then CRC cells were subjected to a limiting dilution assay to test their tumor-repopulating capability. The incidence of tumors in mice was monitored for 16 weeks and determined by performing a definitive necropsy. LDA graphs were generated and statistical analyses were performed using online software provided by Walter+Eliza Hall Bioinformatcis (http://bioinf.wehi.edu.au/software/elda/).

For the liver metastasis mouse model, shCTRL- or shPTPRC-transfected HCT116-luc cells (1×10^6^ cells/mouse) were inoculated into the spleen followed by splenectomy, and the surviving cells that grew in distant organs then contributed to the formation of liver metastases. Liver metastasis was routinely monitored weekly by visualizing luciferase activity for 28 days (n = 9 mice for shCTRL, n = 8 mice for shPTPRC). After sacrifice, the livers were removed to determine liver metastasis.

### Statistical analyses

The results are presented as the means ± standard errors of the means (SEM) for *in vivo* experiments or standard deviations (SD) for *in vitro* experiments. Statistical comparisons between two groups were performed using Student's *t-test* or using one-way ANOVA with Dunnett's multiple comparison tests for 3 or more groups. The log-rank test was used for the Kaplan-Meier analysis. Asterisks are used to indicate statistical significance. *, **, and *** indicate p-values < 0.05, < 0.01, and < 0.001, respectively.

Other detailed descriptions of the materials and methods, including analyses of single-cell RNA-sequencing data, fluorescence-activated cell sorting, organoid cultures, and clinical examinations, are provided in the Supplementary Materials and Methods. The lists of antibodies, short interfering RNA (siRNA) sequences, and primers used for real-time polymerase chain reaction (RT-qPCR) are provided in Tables S3-S5, respectively.

Supplementary information is available at *Theranostics*' website.

## Results

### CD45 expression is increased in surviving CRC epithelial cells after CRT

**Figure 1:**
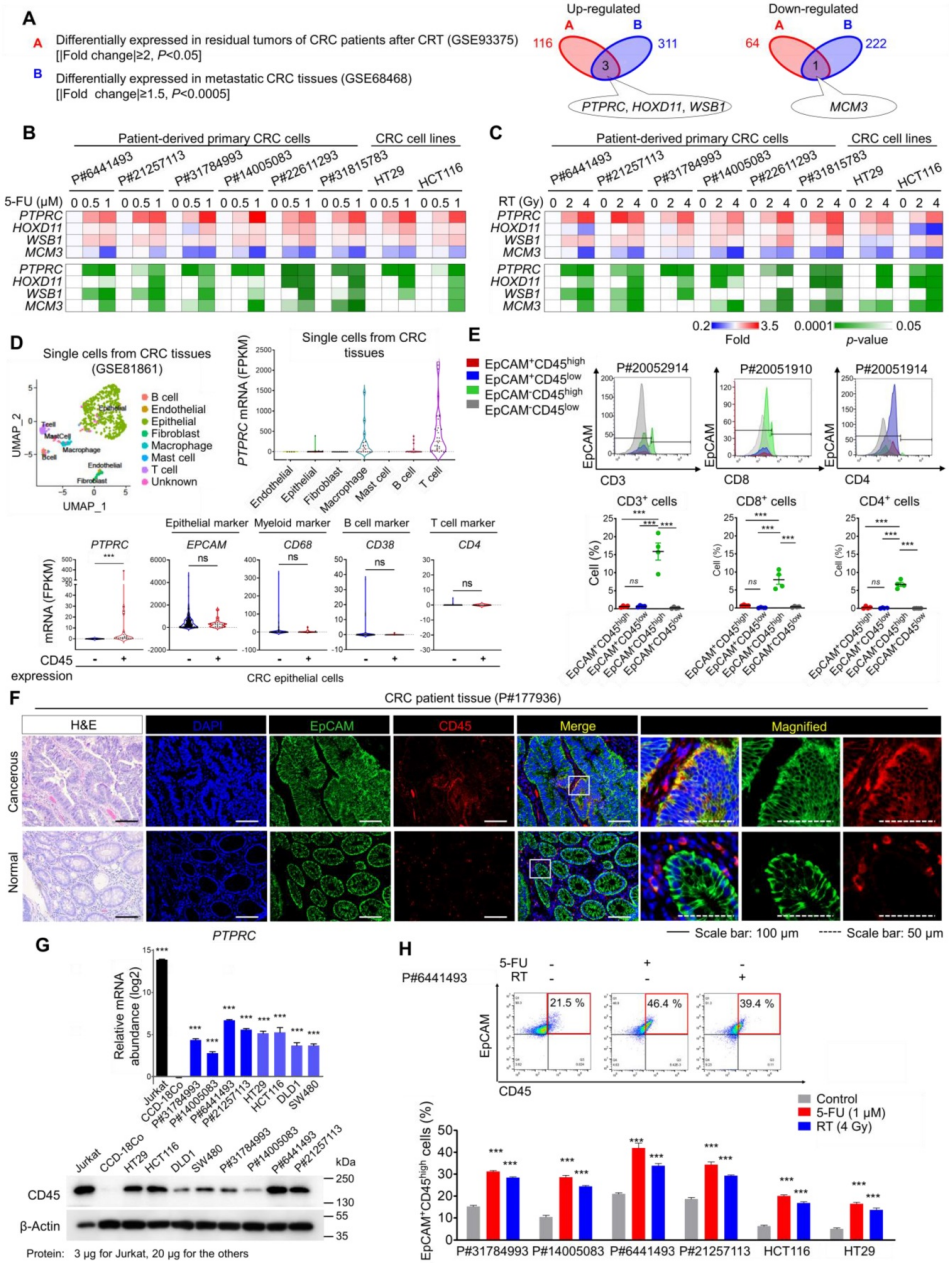
** CD45 expression is increased in surviving CRC epithelial cells after CRT. (A)** Identification of potential therapy resistance-related genes using gene expression data from patients with CRC. The DEG list identified in residual tumors after CRT (upregulated: 116, downregulated: 64, |fold change| ≥2, *p*-value< 0.05) was compared with that of metastatic tumors (upregulated: 311, downregulated: 222, |fold change| ≥1.5, *p*-value< 0.0005). In patients with rectal cancer who underwent preoperative CRT (GSE93375), DEGs were obtained by comparing the residual primary cancer tissues after CRT (n = 9) with the pretreated tissues (n = 13). In patients with colon cancer (GSE68468), DEGs were acquired by comparing nonmetastatic colon cancer (n = 183) and metastatic colon cancer (n = 58) residing in the liver or lung. **(B and C)** RT-qPCR validation of therapy resistance genes in multiple CRC cells at 48 h after (B) 5-FU or (C) radiation treatment. The mRNA expression levels of the indicated genes are presented in a heatmap with *p*-values. **(D)** The scRNA-seq data of CRC tumors were obtained from the GEO web server (GSE81861). Distribution of the expression of *PTPRC* and epithelial and leukocyte markers in the indicated subsets. **(E)** Triple-staining FACS analyses were performed in CRC tumor tissues as follows: (i) CD3, EpCAM, and CD45; (ii) CD4, EpCAM, and CD45; and (iii) CD8, EpCAM, and CD45. The dot blot shows the percentage of the indicated cellular population in tissues from patients with CRC (P#20051910, P#20051914, P#20052910, and P#20052914). **(F)** H&E and immunofluorescence staining of normal and cancerous tissues obtained from patients with CRC. **(G)** RT-qPCR and Western blot analysis of CD45 (*PTPRC*) expression in various CRC cells (n = 3 per group). **(H)** FACS plots show the increase in levels of the CD45 protein in CRC epithelial cells at 48 h after 5-FU or radiation treatment. ** and *** indicate p-values < 0.01 and < 0.001, respectively.

From the gene expression profiles of CRC patients, we obtained a therapy-resistance gene signature by comparing primary tumors and residual tumors after CRT and obtained a metastatic gene signature by comparing metastatic CRC tissues in distant organs with primary tumors (Table S1). Through the comparison, we found that *PTPRC*, homeobox D11 (*HOXD11*), and WD repeat and SOCS box-containing protein 1 (*WSB1*) were commonly upregulated and minichromosome maintenance complex component 3 (*MCM3*) was downregulated in residual CRC tissues after CRT and in metastatic tissues (Figure 1A). Using different human CRC cell lines and multiple patient-derived primary CRC cells isolated from primary tumors (Figure S1A and Table S2), we confirmed the most significant increase in *PTPRC* expression in both 5-fluorouracil (5-FU)-treated and irradiated CRC cells, with consistent results obtained for all tested cells (Figures 1B-C).

The *PTPRC* gene encodes the CD45 protein, which is an early marker of hematopoietic lineage cells. Thus, we explored the single-cell RNA-sequencing data of 375 cells isolated from 11 primary tumors from CRC patients to analyze CD45 expression in various cellular compartments within CRC tissues. Among 272 CRC epithelial cells, we discovered a CD45-expressing subpopulation (7.35%) that did not express other hematopoietic lineage markers (Figures 1D and S1B). Next, we isolated single cells from tumors derived from CRC patients and performed a triple staining FACS analysis for CD45, EpCAM (a conventional epithelium marker), and leukocyte markers. Consequently, we repeatedly detected a CD45-expressing subpopulation (EpCAM^+^CD45^high^) that was negative for CD3, CD4 or CD8 expression (Figures 1E and S1C). We compared CD45 expression between patient-derived CRC tissues and matched normal colorectal tissues to obtain greater insight into this finding. The proportion of EpCAM^+^CD45^high^ cells was significantly increased in CRC tissues (7.58-9.40%), but these cells were rarely detected in normal colorectal tissues (1.62-2.58%, Figure S1D). Consistently, images of immunofluorescence staining showed EpCAM^+^CD45^high^ cells within primary tumor tissues from CRC patients, while they were rarely detected in matched normal colorectal tissues (Figures 1F and S1E). Additionally, the EpCAM^+^CD45^high^ population was significantly increased in adenomatous intestines of APC^Min/+^ mice (9.82-13.50%) compared with normal intestines of wild-type mice (2.01-2.78%, Figure S1F).

Next, we examined CD45 expression in patient-derived primary CRC cells and used the Jurkat T-leukemic cell line as a positive control. All Jurkat cells expressed CD45 but not EpCAM (Figure S1G), while all patient-derived CRC cells expressed EpCAM, suggesting their epithelial origin. Notably, we confirmed that the CD45-expressing population lacked the expression of other leukocyte markers in all tested CRC cells (Figure S1G). Furthermore, CD45 mRNA and protein levels were significantly upregulated in all tested CRC cells compared to CCD-18Co cells (a normal colonic epithelial cell line, Figures 1G and S1H). More importantly, CD45 expression was significantly increased in CRC cells after 5-FU or radiation treatment (Figures 1H and S1I). Collectively, our data clearly show CD45 upregulation in CRC epithelial cells surviving after CRT.

### High CD45 expression in CRC epithelial cells is associated with a poor response to preoperative CRT

**Figure 2:**
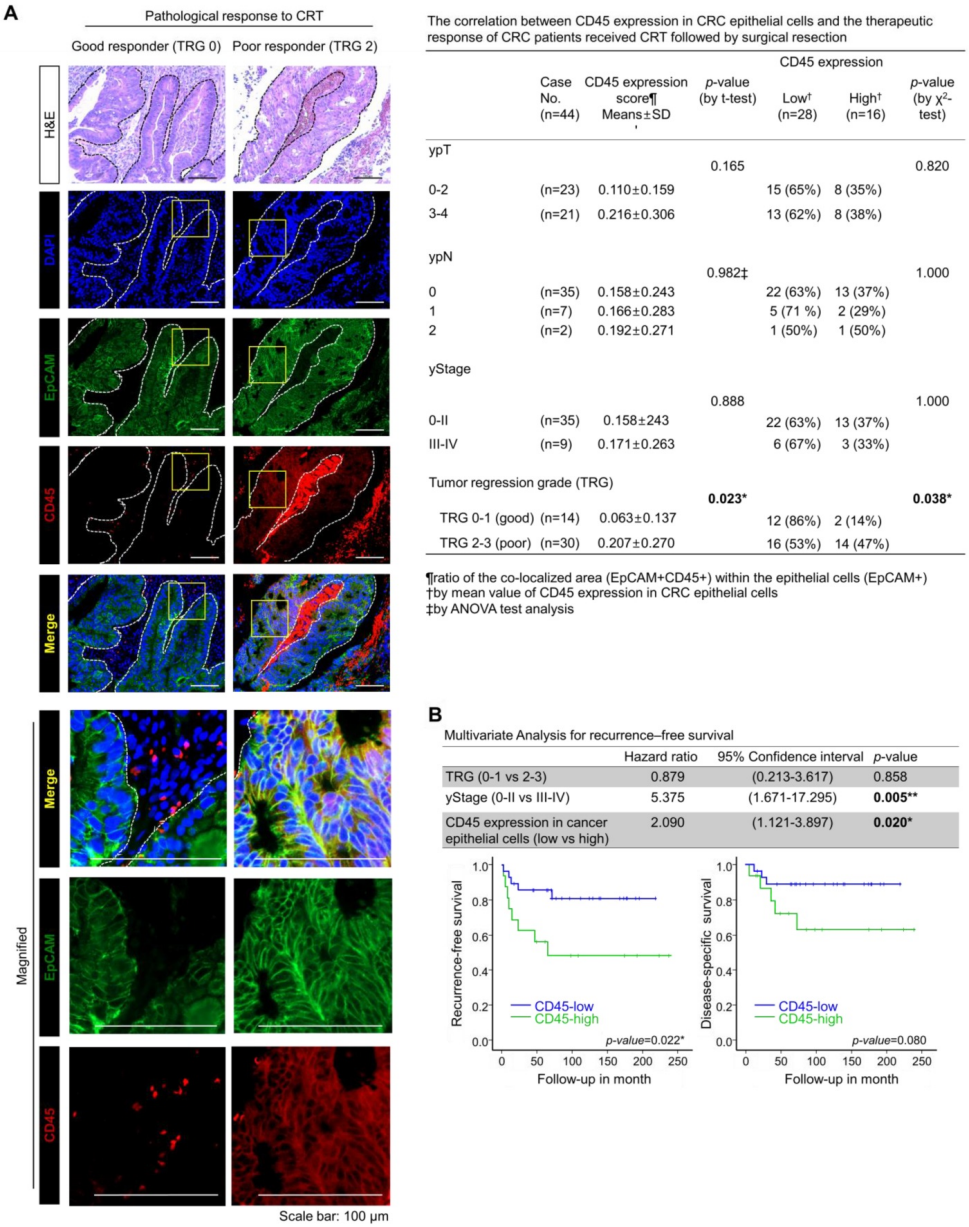
** Higher CD45 expression in rectal cancer epithelial cells confers a poor therapeutic response to preoperative CRT. (A)** A total of 44 pretreatment paraffin-embedded tissue samples obtained from patients with locally advance distal rectal cancer were subjected to immunofluorescence staining for CD45 and EpCAM to determine the relevance of CD45 expression in cancer cells to clinical outcome. The extent of therapeutic response to CRT was pathologically evaluated according to the AJCC TRG scoring. TRG 0 (complete response) and -1 (near complete response) were considered good responses, while TRG 2 (partial response) and -3 (poor or no response) were considered poor responses. Representative immunofluorescence images from the good responder (TRG 0-1) and poor responders (TRG 2-3) groups are presented in the left panel. H&E counterstaining was used to distinguish normal and tumor regions. The CD45 expression levels were scored based on the ratio of the colocalized area (EpCAM+CD45+) within the entire cancer epithelial cell area (EpCAM+) using Image-Pro Premier 9.0 software (Media Cybermetics, right panel, 3-5 spots/sample). The CD45^high^ and CD45^low^ groups were divided by the mean ratio of the colocalized area (EpCAM+CD45+) within the epithelial cells (mean value=0.16096±0.24376; high: n = 16, low: n = 28). The correlation between CD45 expression and TRG was analyzed using the χ2 test. **(B)** The multivariate analysis of recurrence-free survival indicated that the CD45 expression level in rectal cancer epithelial cells was significantly associated with shorter recurrence-free survival in 44 patients (hazard ratio 2.090; 95% confidence interval 1.121-3.897; p=0.020, upper panel). Kaplan-Meier plots also show the recurrence-free (lower left) and disease-specific (lower right) survivals of 44 patients with rectal cancer treated with preoperative CRT followed by surgical resection, according to the CD45 expression levels in primary tumor epithelial cells (*p*=0.022 and *p*=0.08, respectively).

To examine the relevance of CD45 expression to patient clinical outcome, we explored the RNA-sequencing data of CRC patients using the public database, R2: Genomic analysis and visualization platform (https://hgserver1.amc.nl/cgi-bin/r2/main.cgi). As a result, we found a positive correlation of CD45 expression with poor recurrence-free survival in patients with colon cancer (GSE39582, Figure S2A). However, the RNA-sequencing data of patient tumors reflect gene alterations comprehensively derived from diverse cellular compartments such as cancer cells and stromal cells. Thus, we investigated the association between CD45 expression in CRC epithelial cells and the clinical outcomes of CRC patients who underwent preoperative CRT followed by curative surgery. CD45 expression was scored in 44 pretreatment rectal cancer biopsy samples by performing costaining with EpCAM antibodies (Figure 2A), and the tumor regression grade (TRG) was pathologically determined in surgically removed specimens according to the American Joint Committee on Cancer tumor regression grading system 15. The clinicopathological characteristics of patients are described in Figure S2B. As a result, the mean ratio of the colocalized area (EpCAM^+^CD45^+^) within (EpCAM^+^) epithelial cells was determined to be 0.16096±0.24376, and more importantly, the CD45 staining intensity among cancer epithelial cells was higher in poor responders (TRG 2-3, 20.69%) than in good (complete or near complete) responders (TRG 0-1, 6.26%, Figure 2A). Notably, higher CD45 expression was positively correlated with a poorer TRG suggesting therapeutic resistance (Figure 2A); however, CD45 expression in cancer epithelial cells was not associated with post-therapeutic tumor stage (yStage), depth of tumor invasion (ypT) or lymph node metastasis (ypN). In a multivariate analysis of recurrence-free survival, TRG was not significantly associated with recurrence-free survival; however, advanced yStage (III-IV) and high CD45 expression in cancer cells were significantly associated with shorter recurrence-free survival (Figure 2B, upper panel). This result suggests that the amount of residual tumor after CRT was not necessarily related to the risk of recurrence, supporting the hypothesis that even a small subpopulation harboring a therapeutic resistance phenotype, such as cancer stem-like cells, may survive CRT and could be associated with a higher risk of recurrence after CRT 6-10. Patients with high CD45 expression showed significantly shorter recurrence-free survival than patients with low CD45 expression (p=0.022, Figure 2B, lower left panel), and the disease (rectal cancer)-specific survival also tended to be lower in the CD45 higher expression group than the lower expression group (p=0.08, Figure 2B, lower right panel). Although further clinical validation with a larger number of CRC patients including pre-treatment and post-treatment tissues is still necessary to verify the clinical implication that CD45 represents an independent predictive biomarker of a poor clinical outcome, our retrospective study is the first to propose a positive correlation between CD45 expression in cancer cells and a poor response to CRT in CRC patients.

### CD45^high^ CRC cells display a therapy resistance phenotype

**Figure 3:**
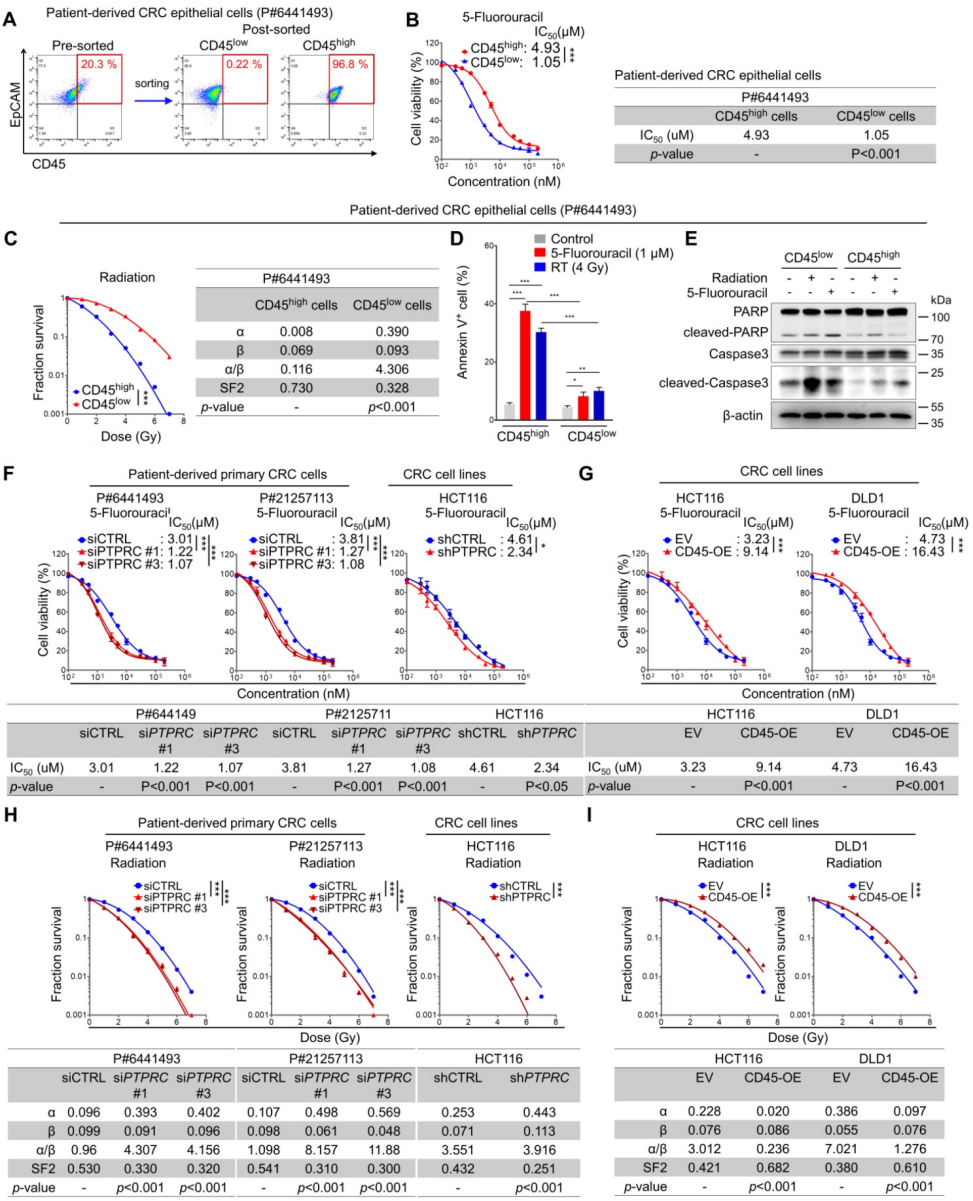
** CRC cells with high CD45 expression display resistance to CRT. (A-E)** Intrinsic therapy resistance was compared between CD45^high^ and CD45^low^ CRC cells obtained from patients with CRC. (A) Patient-derived primary CRC epithelial cells were divided into two groups according to CD45 expression levels. (B) MTT assays showed that CD45^high^ CRC cells were more resistant to 5-FU than CD45^low^ CRC cells. IC_50_ values for 5-FU and significant differences between the two groups were calculated using GraphPad Prism software version 5 (n = 3/group). (C) Radiation dose-survival curves for CD45^high^ and CD45^low^ CRC cells. The radiation-related biological parameters and significant differences between the two groups were analyzed using a linear-quadratic model (n = 3/group). (D and E) The percentage of apoptotic cells at 24 h after 5-FU or radiation treatment was visualized (D) by performing Annexin V staining (n = 3/group) or (E) by visualizing the level of cleaved PARP or cleaved caspase 3. **(F-I)** The functional role of CD45 in CRC therapy resistance was determined by inducing CD45 knockdown or overexpression in multiple CRC cells. The IC_50_ values for 5-FU were determined in (F) CD45 knockdown CRC cells and (G) CD45-overexpressing (OE) CRC cells as described in (B). Radiosensitivity was determined in (H) CD45 knockdown or (I) CD45-OE CRC cells as described in (C). * and *** indicate p-values < 0.05 and < 0.001, respectively. EV, empty vector.

We isolated CD45^high^ and CD45^low^ populations from patient-derived primary CRC cells (Figure 3A) and compared their relative sensitivity to 5-FU by determining the half-maximal inhibitory concentration (IC_50_) values based on reductions in cell viability to investigate the potential association between CD45 and intrinsic therapy resistance. Similarly, their sensitivity to radiation was compared using traditional methods; the survival potential of irradiated cells was estimated with clonogenic assays, and radiation-related biological parameters and statistical significance were then analyzed using a linear-quadratic model. The CD45^high^ population was significantly more resistant to 5-FU and radiation than the CD45^low^ population (Figures 3B-C, S3A-B). Consistently, 5-FU- or radiation-induced apoptosis was more significantly increased among CD45^low^ cells than among CD45^high^ cells (Figures 3D-E). In addition, 5-FU or radiation-induced reduction of cell proliferation was more significantly increased among CD45^low^ cells than among CD45^high^ cells (Figure S3C). Interestingly, interference with the DNA damage response via treatment with a CHK1 inhibitor ameliorated the therapy resistance phenotype of CD45^high^ cells (Figure S3D) and attenuated the increase in CD45 mRNA levels in viable cells after CRT (Figure S3E). These data suggest that the CD45^high^ subpopulation exhibits a therapy-resistant phenotype and manages to survive through CRT, leading to an enrichment of the CD45^high^ subpopulation and an increase in CD45 expression in remnant CRC cells after CRT.

Next, we silenced CD45 expression in CRC cells using siRNAs or shRNA vector (Figure S3F-S3I). CD45 knockdown successfully sensitized CRC cells to 5-FU and radiation (Figure 3F), while CD45 overexpression promoted a more resistant phenotype (Figures 3G, S3J-K). Similarly, CD45 knockdown or overexpression also regulated the surviving fraction of CRC cells after radiation, suggesting the contribution of CD45 to radioresistance (Figures 3H-I). Further Western blot analyses showed that control, CD45-depleted, and -overexpressing cells had a similar DNA damage content (gamma-H2AX) immediately after radiation exposure; however, after 24 hours, extent of DNA damage was more noticeably reduced in CD45-overexpressing cells than in control cells but remained high in CD45-depleted cells, suggesting that high CD45 expression enhances the therapy-resistant phenotype of CRC cells (Figure S3L). Interestingly, CD45 knockdown also attenuated CRC growth under serum-limited conditions (0.1% FBS) and ultralow attachment conditions, all of which are known to induce cellular stress and death in epithelial cells 16-21 (Figures S3M-O), suggesting that high CD45 expression enhanced CRC cell growth under stressful conditions, such as therapy treatment, serum deprivation, and ultralow attachment conditions.

Additionally, we examined the effect of CD45 pharmacological inhibition on cancer cell survival by using a selective CD45 inhibitor, NQ-301 22. NQ-301 treatment successfully sensitized CRC cells to 5-FU and radiation (Figures S3P-Q). NQ-301 treatment also significantly increased 5-FU- or radiation-induced apoptosis (Figures S3R-U). These data suggest that pharmacological inhibition of CD45 may attenuate cancer cell survival.

### CD45 expression enhances the stemness of CRC cells

**Figure 4:**
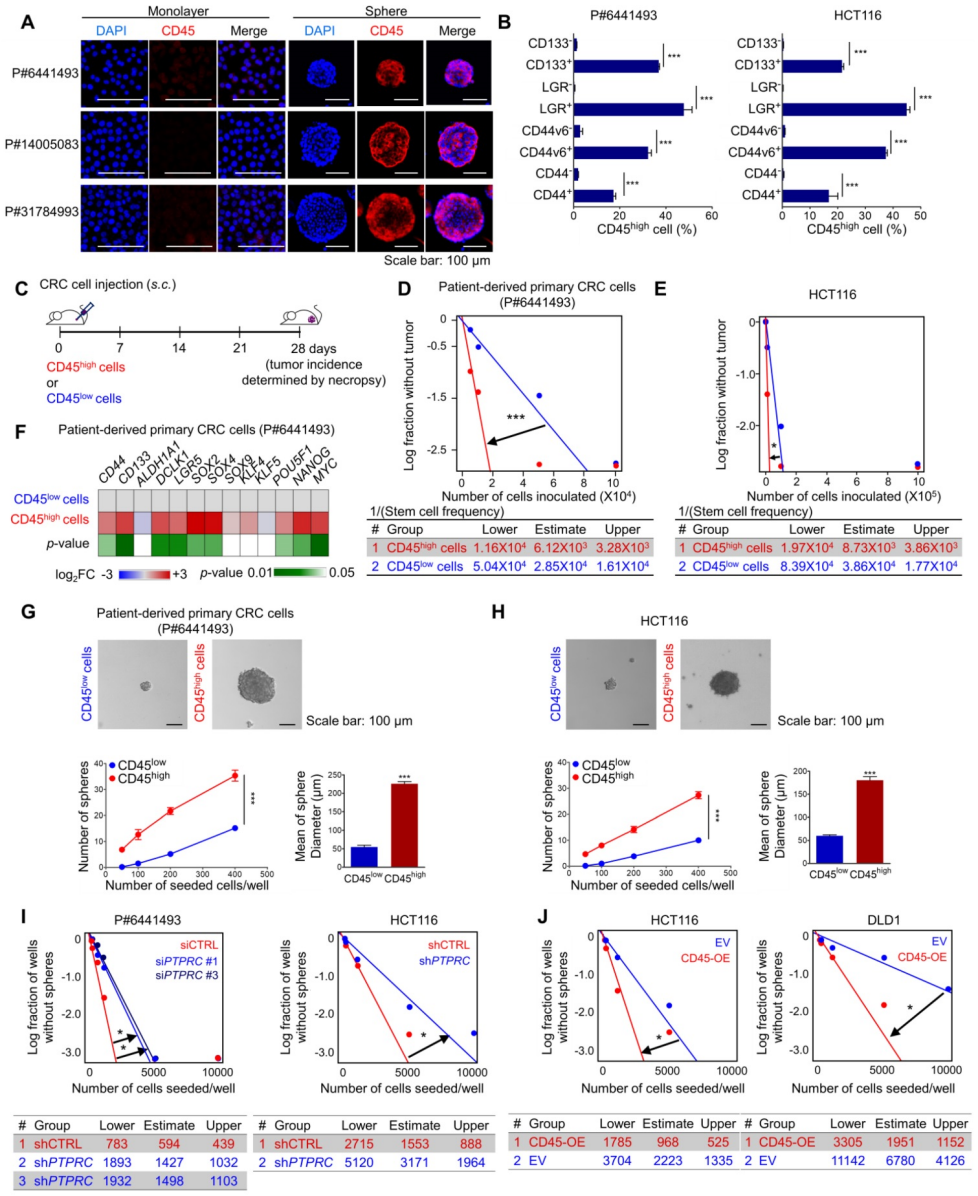
** CD45 expression increases cancer stemness in CRC cells. (A)** Immunofluorescence staining for CD45 in CSC-enriched spheres and in monolayer bulk cells. **(B)** FACS analysis showing that CSCs (CD133^+^, LGR5^+^, CD44s+, and CD44v6^+^) included a greater percentage of CD45^high^ cells than their CSC marker-negative counterparts (n = 3/group). **(C)** Schematic images of the *in vivo* limiting dilution assay (n = 8/group). **(D and E)** Plots of the limiting dilution assay show the higher tumor-initiating potential of CD45^high^ CRC cells than their CD45^low^ counterparts. **(F)** A heatmap shows the transcript levels of stem-related genes in CD45^high^ CRC cells relative to those in CD45^low^ CRC cells (n = 3/group). **(G and H)** Sphere-forming potentials of CD45^high^ and CD45^low^ cells were compared using (G) patient-derived primary CRC cells and (H) HCT116 cells (n = 6/group). **(I and J)** *In vitro* limiting dilution assays were performed to compare the sphere-forming potential (I) between CD45 knockdown and control CRC cells and (J) between CD45-OE and control CRC cells (n = 12/group). * and *** indicate p-values < 0.05 and < 0.001, respectively.

Accumulating evidence suggests that CSCs are resistant to CRT-induced cell death 4, 5. We performed CSC enrichment with CRC cells using methods described in previous reports to investigate the relevance of CD45 to the CSC phenotype 23, 24. The transcriptional comparison suggested a global trend of CSC enrichment in spheres rather than in whole bulk cells by showing an increase in the expression of CSC surface markers and stem-cell-related transcription factors (TFs) and a decrease in the expression of differentiation markers (Figure S4A). In this set of experiments, we also observed an increase in CD45 expression in CSC-enriched spheres at the mRNA and protein levels (Figures 4A, S4A and S4B). Consistent with these findings, FACS analyses showed that CD45 was preferentially expressed in CD133^+^, LGR5^+^, CD44v6^+^, and CD44^+^ CSCs compared with their counterparts (Figures 4B and S4C). We performed an *in vivo* limiting dilution assay using CD45^high^ and CD45^low^ CRC cells isolated from patient-derived primary CRC cells and HCT116 cells to further confirm the characteristic functions of CD45 in maintaining stemness (Figure 4C). Consequently, the CD45^high^ population promoted tumor formation at a higher frequency and produced a larger tumor burden than the CD45^low^ population (Figures 4D-E, S4D-E). Additionally, increases in the expression of various CSC markers and pluripotent TFs were observed in the CD45^high^ population compared with the CD45^low^ counterpart (Figure 4F). The CD45^high^ population generated spheres *in vitro* at a greater number and with a larger size than CD45^low^ CRC cells, suggesting their superior sphere-forming potential (Figures 4G-H). Moreover, when we repeated the isolation of CD45^high^ and CD45^low^ cells from HCT116 spheres to generate second-passage spheres, we confirmed that the CD45^high^ population exhibited greater sphere-forming potential than the CD45^low^ population (Figure S4F). Furthermore, spheres generated from CD45^high^ CRC cells consisted of both CD45^high^ and CD45^low^ CRC populations with a ratio similar to that of the initial CD45^high^ and CD45^low^ populations, while cancer cells generated from a CD45^low^ population only consisted of a CD45^low^ population (Figure S4G). Similarly, the histopathological analysis of HCT116 xenografts revealed that tumor tissues generated from the CD45^high^ population consisted of both CD45^low^ and CD45^high^ populations, while the CD45^high^ population was not observed in tumor tissues generated from the CD45^low^ population (Figure S4H). Furthermore, CD45 gene silencing abrogated sphere-forming potential in both CRC cells from patients and HCT116 cells (Figure 4I), while CD45 overexpression increased the sphere-forming potential (Figure 4J). Additionally, CD45 pharmacological inhibition with NQ-301 treatment significantly attenuated sphere-forming potential in both patient-derived CRC cells and HCT116 cells (Figure S4I). Together, these data suggest that CD45 expression is linked to the CSC phenotype of CRC cells.

### Targeting CD45 abrogates CRC initiation, repopulation, and metastasis by disrupting stemness

**Figure 5:**
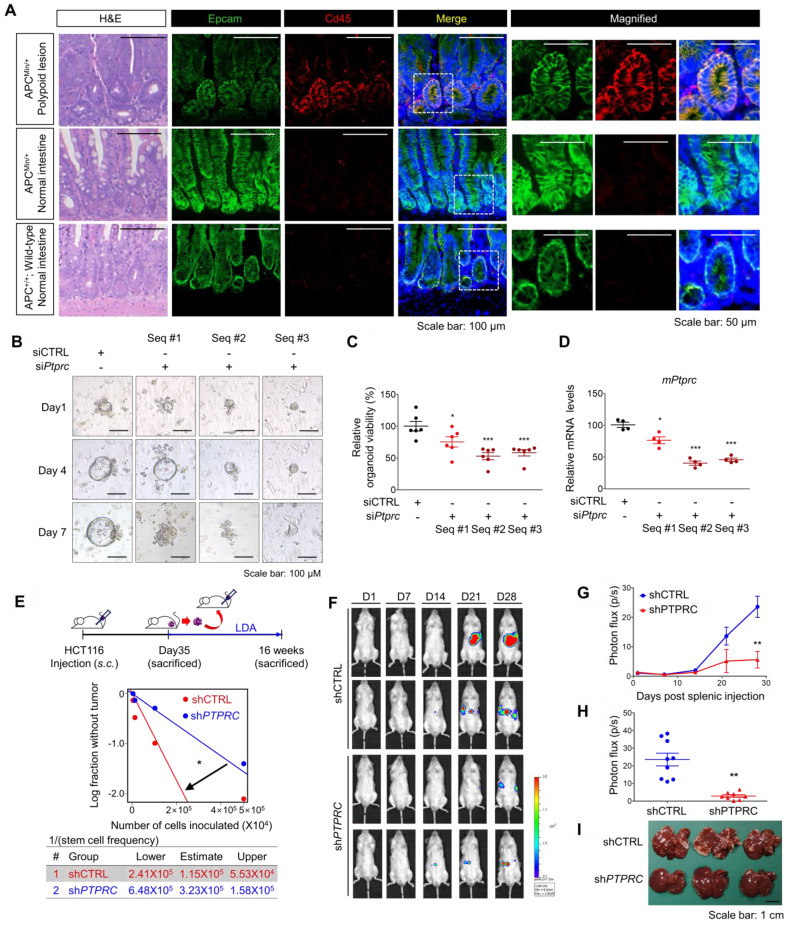
** CD45 inhibition attenuates the CSC properties of cancer initiation, repopulation, and metastasis. (A)** H&E and immunofluorescence staining of polypoid and normal tissues obtained from APC^Min/+^ mice and normal intestines from APC^+/+^ wild-type mice. **(B-D)** Single cells were isolated from intestinal polyps of 20-week-old APC^Min/+^ mice and transfected with siCTRL or siPTPRC using an NEPA21 superelectroporator. These cells were seeded and grown under organoid culture conditions. (B) Brightfield images of APC^Min/+^ mouse polyp-derived organoid cultures with or without CD45 knockdown. (C) The viability of organoids was compared by performing a resazurin-based Cell Titer Blue assay on the 7th day of organoid culture (n = 6/group). (D) The CD45 knockdown efficiency was determined on the 3^rd^ day of organoid culture (n = 4/group). **(E)** Serial transplantation assay comparing the tumor-repopulating potential between shCTRL and shPTPRC cells (n = 8/group). **(F-I)** Comparison of the metastatic potential between shCTRL and shPTPRC HCT116-luc cells. Cells were inoculated into the spleen followed by splenectomy (n = 9 for shCTRL, n = 8 for shPTPRC). (F and G) Liver metastasis was monitored weekly by visualizing luciferase activity for 28 days. (H) Luciferase activity on the 28^th^ day after inoculation. (I) Metastatic nodules on the liver were determined after sacrifice. *, **, and *** indicate p-values < 0.05, < 0.01, and < 0.001, respectively.

Next, we employed multiple mouse CRC models to estimate the potential roles of CD45 in tumor initiation, progression, and metastasis. First, we utilized APC^Min/+^ mice, which are highly susceptible to spontaneous intestinal adenoma 25. Intriguingly, we observed an increase in *PTPRC* mRNA levels in APC^Min/+^ polyps compared to normal intestines of wild-type mice (Figure S5A). A subsequent histological assessment revealed high CD45 expression in EpCAM^+^ intestinal epithelial cells within APC^Min/+^ mouse polyps, while CD45 expression was rarely observed in normal intestinal epithelium from wild-type mice (Figure 5A). To examine the functional role of CD45 in the organogenesis of APC^Min/+^ mouse polyps, we isolated single cells from APC^Min/+^ mouse polyps and silenced CD45 expression using three different siRNAs targeting *mPtprc*. On day 1, both control- and siPtprc-transfected cells showed a cyst-like morphology in organoid cultures; however, CD45-depleted cells did not generate an epithelium, and consequently, organoids did not grow and ultimately died (Figure 5B-D). Consistently, CD45 pharmacological inhibition with NQ-301 treatment significantly reduced the growth of organoids derived from APC^Min/+^ mouse polyps (Figure S5B), suggesting that CD45 may be required for active stem cells residing in APC^Min/+^ polyps. Although further investigation is still necessary, our results suggest the potential role of CD45 during CRC tumorigenesis.

In parallel, a series of transplantation assays in HCT116 xenograft mouse models showed that CD45 knockdown attenuated both primary and secondary tumor growth (Figure S5B), with a significant reduction in the tumor-repopulating potential (Figure 5E). Next, we performed a splenic injection experiment, a common murine model, to estimate the step governing metastasis and distant organ colonization 26. CD45 depletion dramatically reduced the colonization potential of CRC cells in the liver, indicating a critical role for CD45 in the metastasis of CRC cells (Figures 5F-I). In order to evaluate the effect of CD45 pharmacological inhibition on the migration and invasion potentials of CRC cells, we carried out Transwell assays. In this experiment, to avoid a reduction in cell number, we chose 0.1 uM NQ-301, which did not influence the viability of patient-derived CRC cells and HCT116 cells (Figure S5D). Consistently, CD45 pharmacological inhibition with NQ-301 treatment reduced the migration and invasion potentials in both CRC cells from patients and HCT116 cells (Figure S5E). Based on these results, we concluded that CD45 expression is required for distinct functions of CSCs, such as cancer initiation, repopulation, and metastasis, in CRC mouse models.

### CD45 enhances CSC-enriched Wnt signaling by promoting β-catenin accumulation

**Figure 6:**
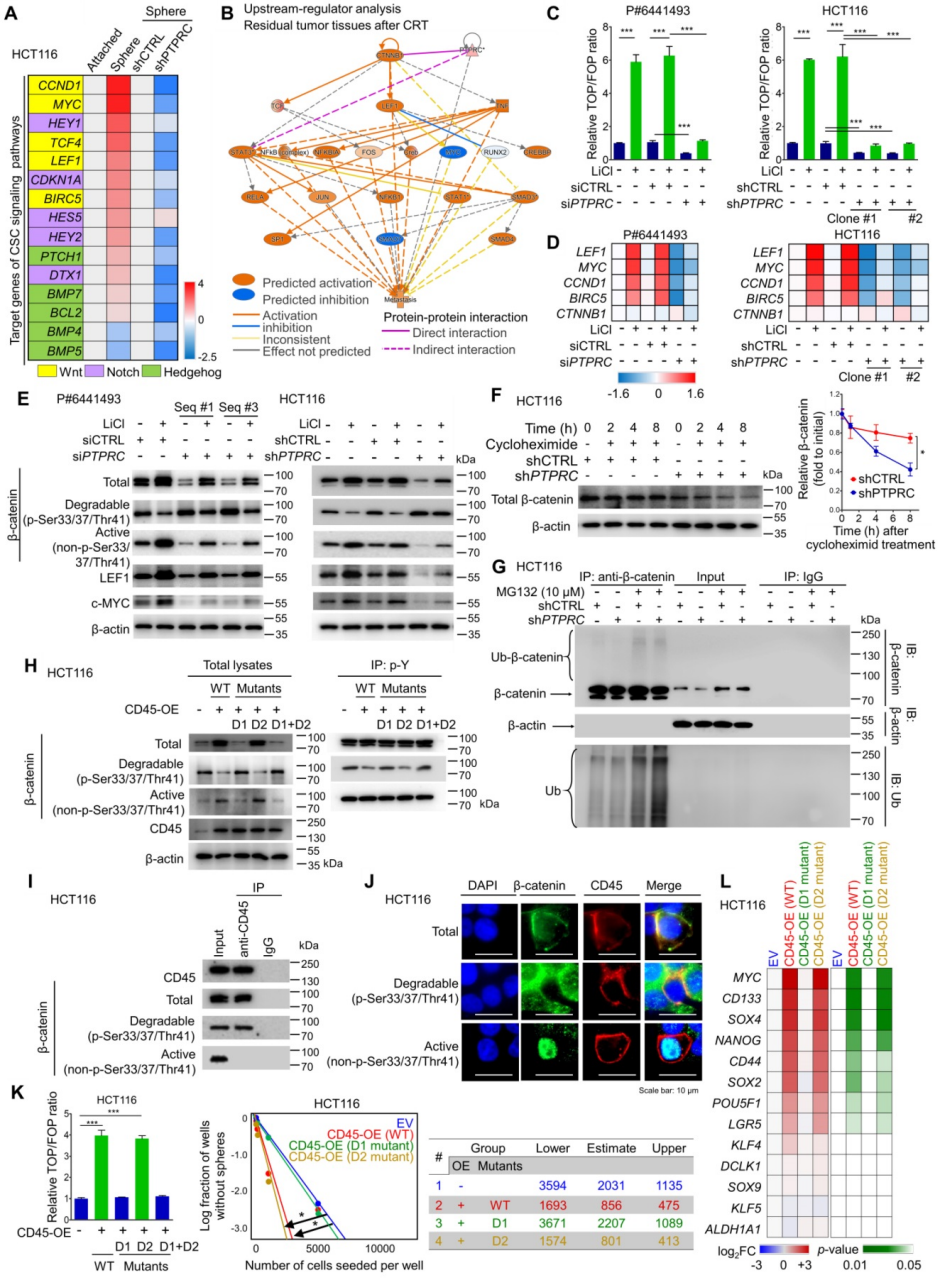
** CD45 expression augments the activation of Wnt/β-catenin signaling in CRC cells. (A)** RT-qPCR screening of transcript levels of target genes involved in well-known CSC signaling pathways, including Wnt/β-catenin, Notch, and Hedgehog signaling (n = 3/group). **(B)** Upstream regulators in CRT-treated tumors were predicted using IPA software based on the change in gene expression in CRT-treated CRC tumors (GSE15781). **(C)** A TOP/FOP assay was performed in CRC cells with or without CD45 knockdown in combination with Wnt activator treatment (10 mM LiCl, 18 h, n = 3/group). **(D)** Relative mRNA expression levels of β-catenin and various Wnt target genes (n = 3/group). **(E)** Western blot assays show total β-catenin levels and its phosphorylation status, as well as the levels of Wnt target proteins. **(F)** The effect of CD45 on β-catenin degradation was measured during protein synthesis blockade by cycloheximide treatment (n = 3/group). **(G)** The effect of CD45 on β-catenin ubiquitination was determined during proteasomal degradation blockade by MG132 treatment. **(H)** The tyrosine phosphorylation status of β-catenin was determined using immunoprecipitation (IP). (F) IP revealed the binding of CD45 to β-catenin. **(J)** Immunofluorescence staining revealed the colocalization of CD45 with β-catenin at cellular membranes. **(K)** The effects of wild-type or mutant CD45 overexpression on the transcriptional activity of Wnt/β-catenin signaling (n = 3/group, left panel) and the sphere-forming potential (n = 12/group, right panel) are shown. **(L)** A heatmap shows the effect of wild-type or mutant CD45 overexpression on the transcript levels of stemness-related genes (n = 3/group). * and *** indicate p-values < 0.05 and < 0.001, respectively.

We performed a set of RT-qPCR screening assays for CSC signaling pathways (Wnt, Notch, and Hedgehog) to identify the CD45-mediated signaling pathway. Consequently, Wnt target genes were most highly enriched in CSCs and concomitantly inhibited by CD45 knockdown (Figure 6A). Additionally, IPA visualized the activation of β-catenin, a major TF involved in Wnt signaling, in residual tumors after CRT (Figure S6A), creating a molecular network with lymphoid enhancer binding factor 1 (LEF1) and the T-cell factor (TCF) complex that contributes to metastasis (Figure 6B). Consistently, both IPA and the STRING database visualized the potential protein-protein interactions between CD45 and β-catenin (Figures 6B and S6B). Therefore, we tested whether CD45 is involved in Wnt/β-catenin signaling by performing multiple validation experiments.

CD45 knockdown diminished Wnt transcriptional activity in patient-derived primary CRC cells and HCT116 cells (Figure 6C). In parallel, the mRNA levels of Wnt target genes were reduced by CD45 knockdown without affecting β-catenin transcript levels (Figure 6D). At the protein level, CD45 knockdown also reduced β-catenin accumulation by increasing levels of phosphorylated β-catenin (degradable form) and reducing levels of active β-catenin, leading to reductions in the levels of Wnt target proteins (Figure 6E). Conversely, CD45 overexpression enhanced Wnt transcriptional activity (Figure S6C), increased active β-catenin levels and reduced degradable β-catenin levels, resulting in increased Wnt target gene expression (Figure S6D). Interestingly, CD45 knockdown accelerated the decrease in β-catenin levels even after cycloheximide treatment, which inhibits protein synthesis (Figure 6F). Further analysis using the proteasome inhibitor MG132 revealed that CD45-knockdown cells showed increased β-catenin ubiquitination (Figure 6G). Collectively, CD45 may promote β-catenin accumulation through protein stabilization rather than effects on its mRNA levels.

According to the UniProt database, CD45 has two distinct tyrosine phosphatase domains, domain I and domain II (Figure S6E). Phosphocysteine intermediates are present in both domains at cysteine 853 and cysteine 1169. However, a cysteine-to-serine mutation in domain I is linked to a loss of CD45 catalytic activity, while domain II mutations do not exert detectable effects 27. Therefore, we tested whether the phosphatase activity of CD45 is critical for β-catenin regulation using CD45 vectors with mutations in domain I (D1), domain II (D2), or both domains I and II (D1+D2). D1 and D1+D2 mutations abrogated the regulatory effects of CD45 on β-catenin accumulation, while D2 mutation alone produced no detectable effect (Figure 6H). Interestingly, CD45 overexpression reduced tyrosine phosphorylation levels of degradable β-catenin but not those of active β-catenin. D1 and D1+D2 mutations abrogated tyrosine dephosphorylation of CD45, while no significant effect was observed for the D2 mutation. This result is also supported by the protein-protein interaction between CD45 and β-catenin, where CD45 binds only to degradable β-catenin and not to active β-catenin (Figure 6I). Further immunofluorescence assays visualized the colocalization of CD45 with only total and degradable β-catenin at the cellular membrane, while active β-catenin was mainly located in the nucleus (Figure 6J), suggesting that membrane-anchored CD45 binds to total and degradable β-catenin but not to active β-catenin. In addition, D1 and D1+D2 mutations disrupted CD45-induced Wnt transcriptional activity and sphere-forming potential (Figure 6K). Moreover, a set of RT-qPCR arrays against stemness-related transcripts revealed that D1 mutation significantly ameliorated the CD45-induced increases in the expression of various CSC markers and pluripotent TFs, while the D2 mutant rarely affected the levels of these transcripts (Figure 6L). Collectively, these data suggest that the phosphatase activity of CD45 is linked to the dynamics of β-catenin, leading to an increase in Wnt signaling and stemness.

Subsequently, β-catenin knockdown sensitized CRC cells to 5-FU and radiation (Figure S6F and S6G), and diminished the sphere-forming potential of CRC cells (Figure S6H). Additionally, CD45 overexpression did not increase sphere-forming efficiency under β-catenin knockdown conditions (Figure S6I). The CD45-induced increase in sphere-forming potential was reduced by treatment with ICG001, an inhibitor of β-catenin-mediated transcription, in a dose-dependent manner (Figure S6J), suggesting that β-catenin accumulation promoted by CD45 serves as a mediator of therapy resistance and stemness. Together, these data indicate that the tyrosine phosphatase activity of CD45 is crucial for enhancing Wnt/β-catenin signaling, which is responsible for stemness and therapy resistance in CRC cells.

## Discussion

In this study, we aimed to identify therapy-resistant genes by exploring clinical genomic profiles and discovered a concomitant increase in CD45 expression in residual tumors after CRT and in metastatic tumors. CD45 has been used as a pan-leukocyte marker for decades. However, its clinical significance in patients with CRC has been ambiguous; the increased infiltration of CD45^+^ immune cells within primary tumors was reported to be associated with a better survival rate in patients with CRC 28, while the comparison of gene expression profiles between metastatic tumors and primary tumors suggested an association between increased CD45 expression and CRC metastasis 29. In this context, through costaining with the epithelial marker EpCAM, we first discovered the existence of a CD45-expressing CRC subpopulation residing in primary tumors from CRC patients (Figure 1D and E). Notably, elevated CD45 expression in CRC epithelial cells was significantly correlated with poor tumor regression and shorter recurrence-free survival in CRT-treated patients (Figure 2). These findings provide a rationale for the use of CD45 as a new prognostic biomarker of therapy resistance.

CRT is one of the most effective therapies for eliminating the tumor burden 30. However, emerging evidence suggests that CSCs are more resistant to CRT than bulk tumor cells and are capable of escaping cytotoxicity, surviving therapy, and promoting metastasis and recurrence after therapy termination 31-33. Therefore, various strategies that directly attack CSCs based on surface markers or indirectly inhibit CSC functions have been investigated 34. Wnt/β-catenin signaling has become a promising target for inhibiting CSCs, along with other CSC signaling pathways 35. As shown in our previous studies, Wnt signaling is critical for maintaining CSC properties, such as self-renewal, metastasis, and chemoresistance; moreover, disrupting the transcriptional activity of Wnt/β-catenin signaling has been widely appreciated as a promising therapeutic strategy against the CSC phenotype 23, 36-39. However, additional investigations are required to explain why Wnt/β-catenin signaling is aberrantly activated in CSCs and to understand how CSC-specific Wnt/β-catenin signaling can be targeted without affecting normal signaling essential for homeostasis and regeneration. This study identified CD45 as a therapy resistance gene in CRC cells and confirmed its contribution to increased Wnt signaling through β-catenin accumulation. β-Catenin regulation is highly complex and organized, with diverse kinases and phosphatases altering the phosphorylation status of β-catenin, which collectively determines the dynamics of β-catenin, such as ubiquitination, degradation, nuclear translocation, and transcription 40, 41. Although the link between tyrosine phosphorylation and β-catenin degradation is not yet clear, we discovered that CD45 expression caused β-catenin accumulation (Figure 6). Additionally, the phosphatase activity of CD45 reduced the tyrosine phosphorylation of degradable β-catenin, thus increasing Wnt transcriptional activity and cancer stemness. Collectively, our results suggest that CD45 provides a new explanation for how CSCs maintain aberrantly activated Wnt signaling. This novel function of the CD45/β-catenin axis could provide a potential therapeutic strategy for CRC treatment. To date, several small molecule inhibitors that specifically inhibits the phosphatase activity of CD45 have been developed and tested in preclinical studies as a means to regulate immune responses such as T cell activation, autoimmunity, organ graft rejection and inflammation 42, 43. Considering that the phosphatase activity of CD45 is critical for multiple leukocyte functions, pan-CD45 phosphatase inhibitors will probably not be applicable for CRC therapy because of adverse effects. In this regard, a more rational approach is required to specifically target the β-catenin regulatory function of CD45. The use of protein-protein interaction (PPI) modulators may be one of potential ways to overcome this challenge 44,45. PPI modulators designed to specifically inhibit the interaction between CD45 and β-catenin may have a profound potential to eliminate the CD45-mediated β-catenin accumulation without affecting the leukocyte functions of CD45. By performing a protein-protein docking simulation with ZDOCK and ZRANK algorithms in the Discovery Studio program (Accelrys, San Diego, CA, USA), we found the possible interaction site between CD45 (PDB code: 1YGR) and β-catenin (PDB code: 1G3J). As shown in Figure S7, the simulation result showed that phosphatase domain D1 of CD45 is more favorable for the interaction of β-catenin. The armadillo repeat domain (ARM) 4-9 of β-catenin (AA 268-505) has the potential to interact with the CD45 phosphatase domain D1 at β-strand β4-β7 (AA 727-757) and loop β11-α3 (AA 793-803). Therefore, further investigation using PPI technology is desired to investigate these possibilities.

In summary, this study provides strong evidence for CD45 expression in CRC epithelial cells and proposes that CD45 promotes certain CSC properties, including tumor initiation, repopulation, metastasis and therapy resistance, at least in part by modulating the phosphorylation status of β-catenin, which results in increased Wnt/β-catenin transcriptional activity. Although several studies have reported the existence of EpCAM^+^CD45^+^ cells in biopsies from patients with cancer, the molecular mechanism underlying the aggressive phenotype of this novel population remains ambiguous 46, 47. In this context, this study advances the understanding of how CD45 expression within cancer epithelial cells supports cancer aggressiveness by highlighting the importance of CD45 as a novel mediator of therapy resistance and cancer stemness.

## Supplementary Material

Supplementary figures and tables.

## Abbreviations

5-FU: 5-Fluorouracil
AJCC: American Joint Committee on Cancer
ALPI: Intestinal alkaline phosphatase
ANPEP: Membrane alanyl aminopeptidase
APC: Adenomatous polyposis coli
BIRC5: Baculoviral inhibitor of apoptosis (IAP) repeat containing 5
c-caspase 3: Cleaved-caspase 3
CCND1: Cyclin D1
CD133: Cluster of differentiation 133
CD44: Cluster of differentiation 44
CD44v6: Cluster of differentiation 44 variant 6
CD45: Cluster of differentiation 45
CRC: Colorectal cancer
CRT: Chemoradiation therapy
CSC: Cancer stem-like cell
CTNNB1: Catenin beta 1
DEG: Differentially expressed gene
EpCAM: Epithelial cell adhesion molecule
FABP1: Fatty acid binding protein 1
HOXD11: Homeobox D11
IC_50_: Half-maximal inhibitory concentration
IPA: Ingenuity Pathway Analysis
LEF1: Lymphoid enhancer binding factor 1
LGR5: Leucine-rich repeat containing G protein-coupled receptor 5
MCM3: Minichromosome maintenance complex component 3
PARP: Poly(ADP-ribose) polymerase
POU5F1: POU class homeobox 1
PTPRC: Protein tyrosine phosphatase receptor type C
SOX2: Sex-determining region Y (SRY)-box 2
SOX4: Sex-determining region Y (SRY)-box 4
TCF: T-cell factor
TRG: Tumor regression grade
WSB1: WD repeat and SOCS box-containing protein 1
