# Supplementary material for: Aberrant activation of the CD45-Wnt signaling axis promotes stemness and therapy resistance in colorectal cancer cells
Source: Theranostics. 2021 Aug 11;11(18):8755–70. doi: 10.7150/thno.63446 (PMC8419050; doi:10.7150/thno.63446)
Supplement: Supplementary file 1 — Supplementary figures and tables. [file thnov11p8755s1.pdf]

Supplementary Information

Aberrant activation of the CD45-Wnt signaling axis promotes stemness and therapy resistance in colorectal cancer cells

So-Yeon Park[1,2], Ji-Young Kim[1], Gyu-Beom Jang[1], Jang-Hyun Choi[1], Jee-Heun Kim[1], Choong-Jae Lee[1], Sunjae Lee[1], Jeong-Heum Baek[3], Kwan-Kyu Park[4], Jin-Man Kim[5], Hee Jin Chang[6], and Jeong-Seok Nam[1,2#]

[1]School of Life Sciences, Gwangju Institute of Science and Technology, Gwangju 61005, Republic of Korea

[2]Cell Logistics Research Center, Gwangju Institute of Science and Technology, Gwangju 61005, Republic of Korea

[3]Division of Colon and Rectal Surgery, Department of Surgery, Gil Medical Center, Gachon University College of Medicine, Incheon 21999, Republic of Korea

[4]Department of Pathology, College of Medicine, Catholic University of Daegu, College of Medicine, Daegu 38430, Republic of Korea

[5]Department of Pathology, College of Medicine, Chungnam National University, Daejeon 34134, Republic of Korea

[6]Research Institute and Hospital, National Cancer Center, Goyang 10408, Republic of Korea

[#]**Corresponding Author**: Jeong-Seok Nam

School of Life Sciences, Gwangju Institute of Science and Technology, Gwangju 61005, Republic of Korea

E-mail: namje@gist.ac.kr

Phone: +82-62-715-2893

Fax: +82-62-715-2484

Supplementary figures

Fig. S1 (continued)

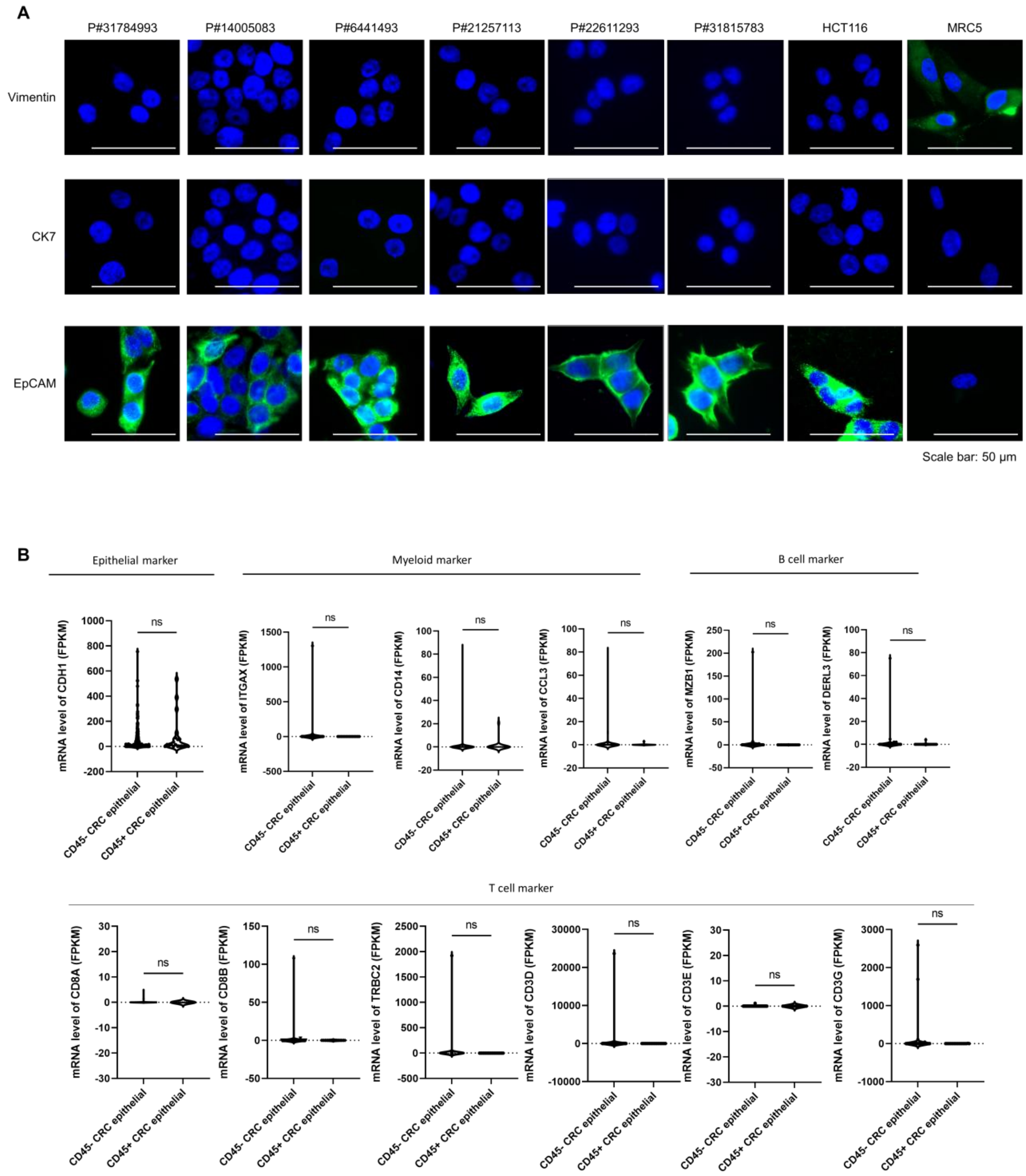

Fig. S1 (continued)

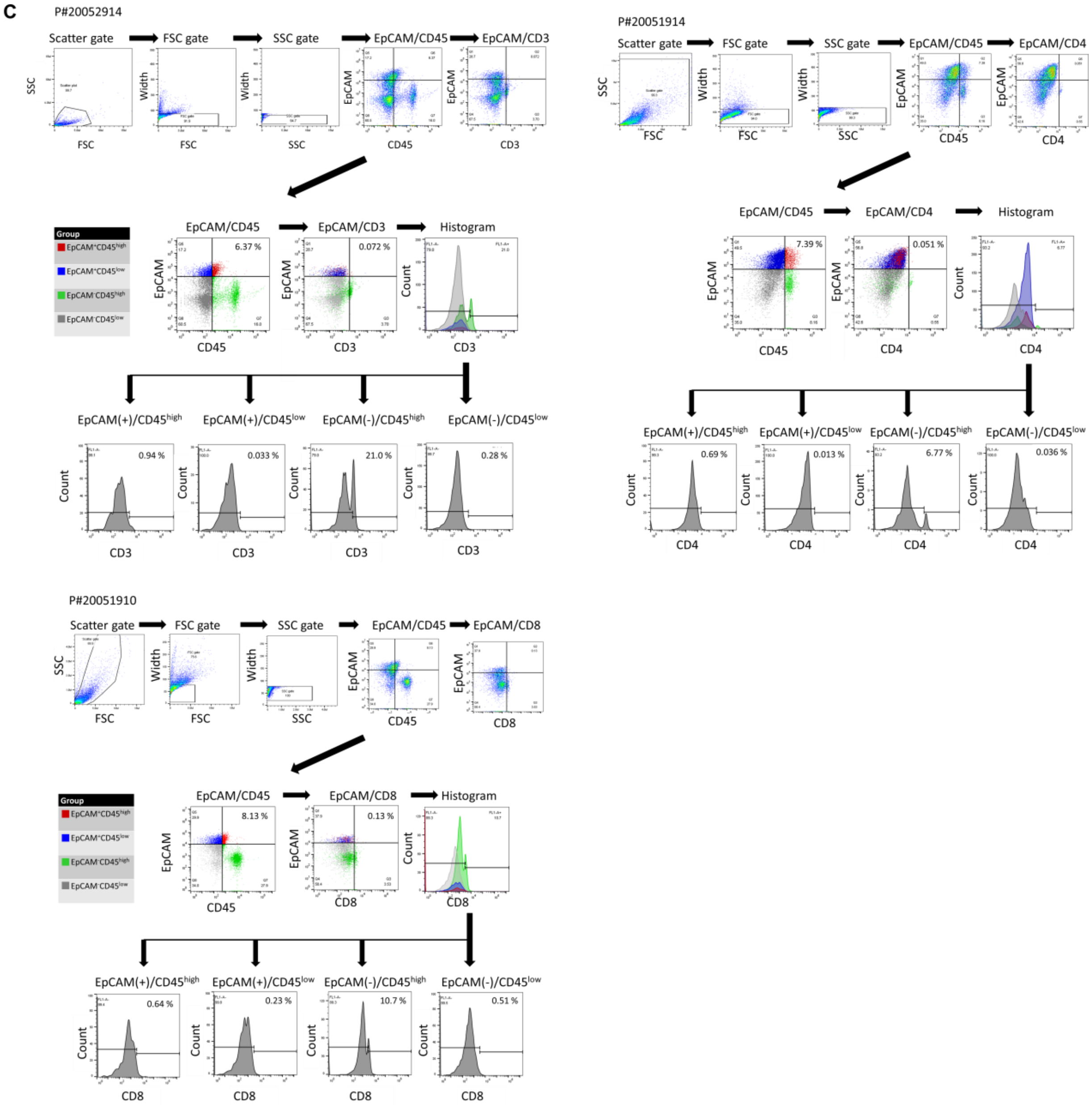

Fig. S1 (continued)

D

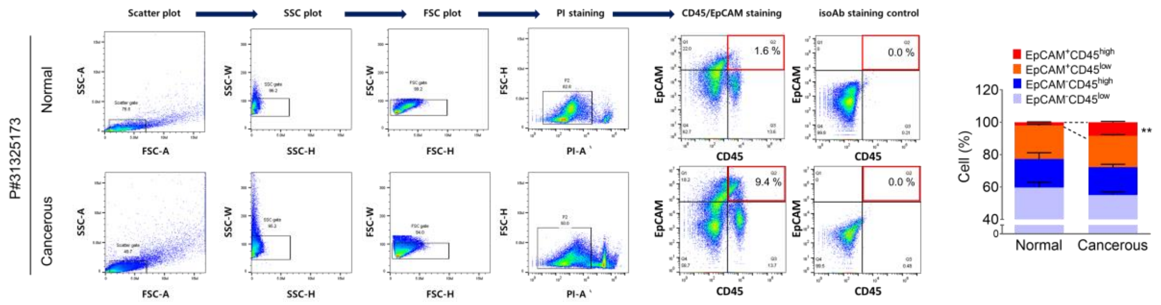

E

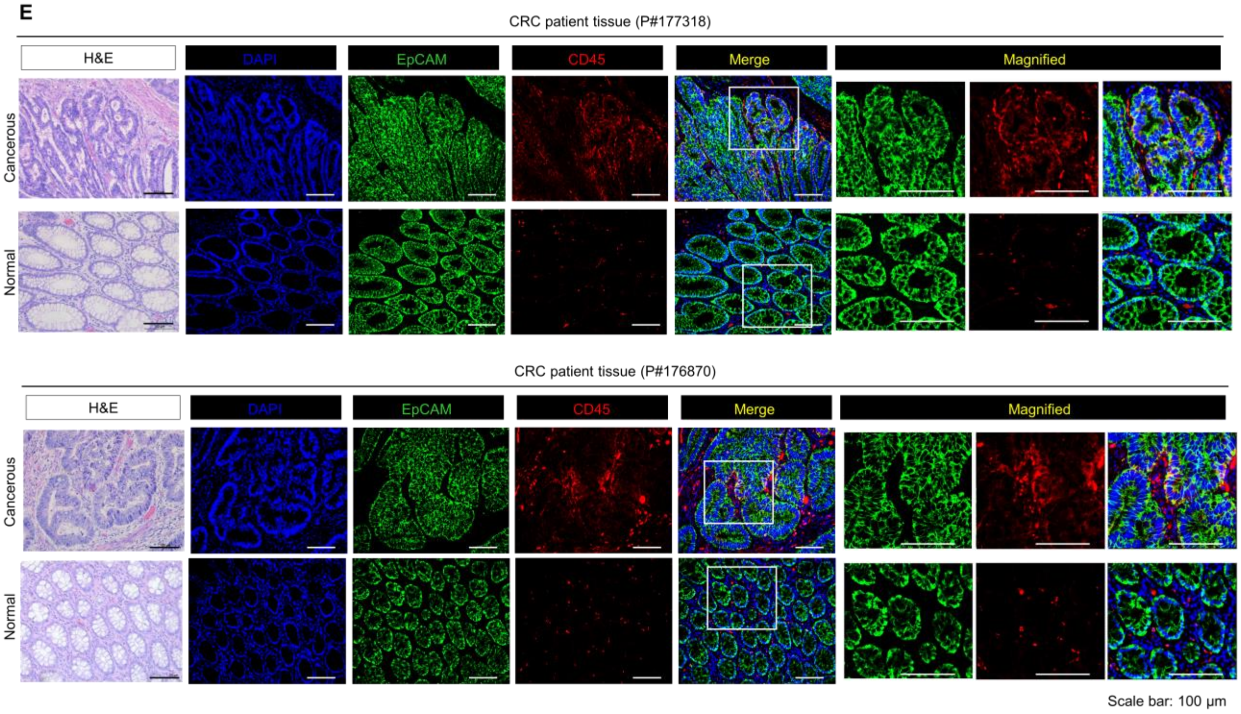

F

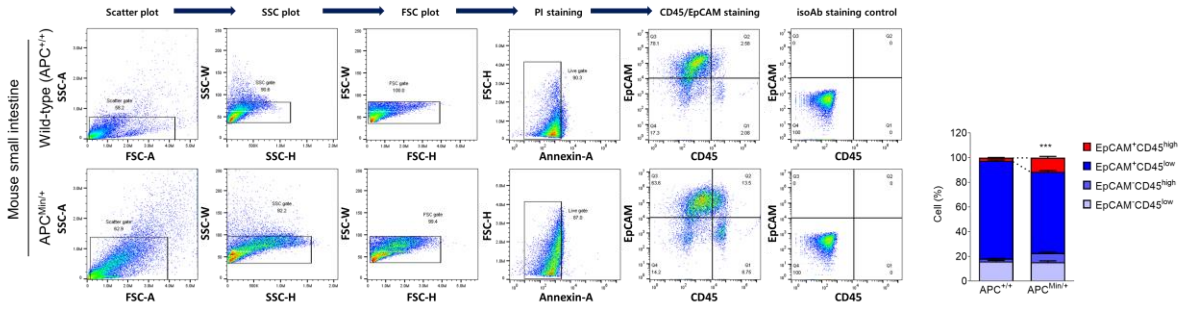

Fig. S1 (continued)

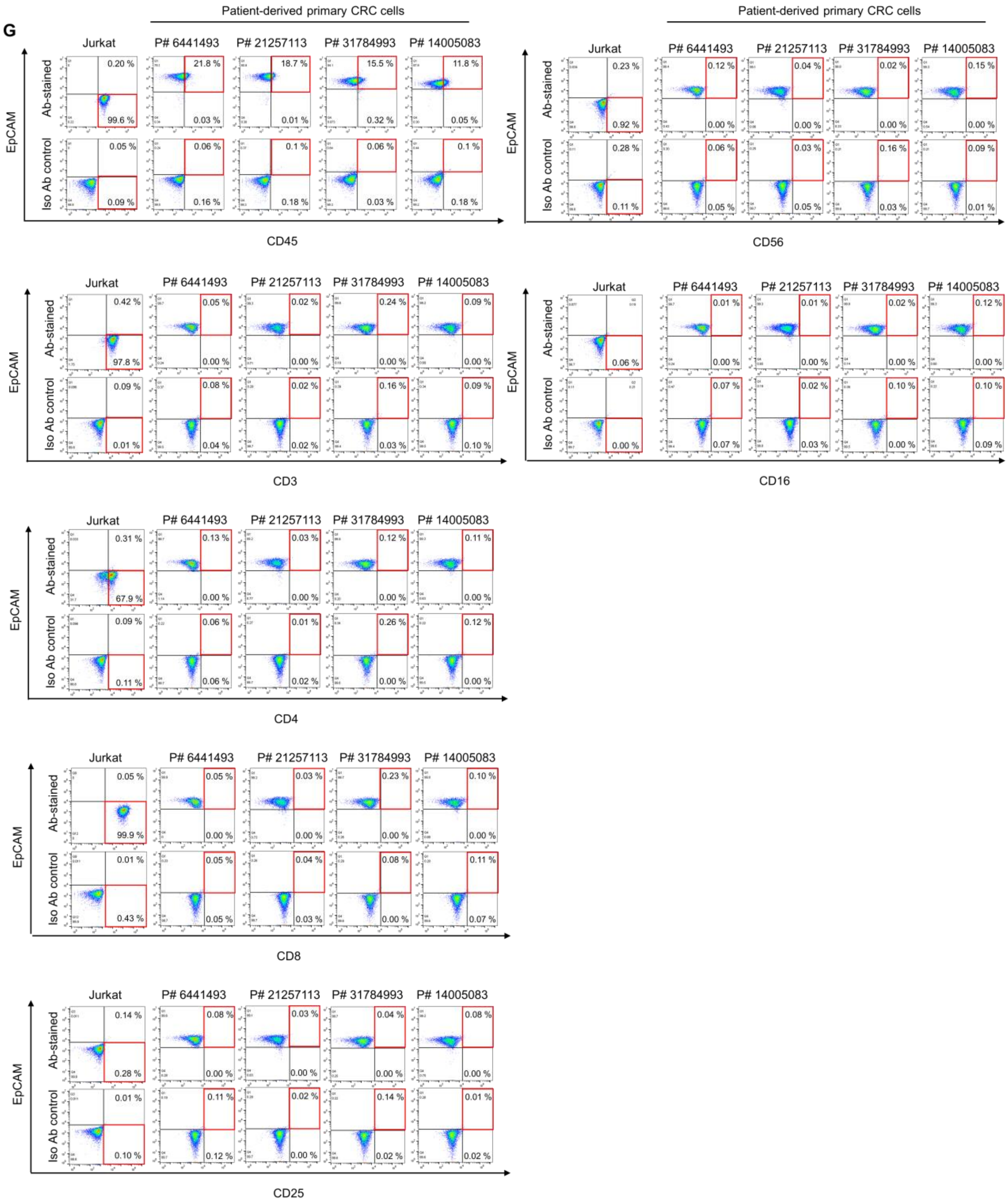

Fig. S1

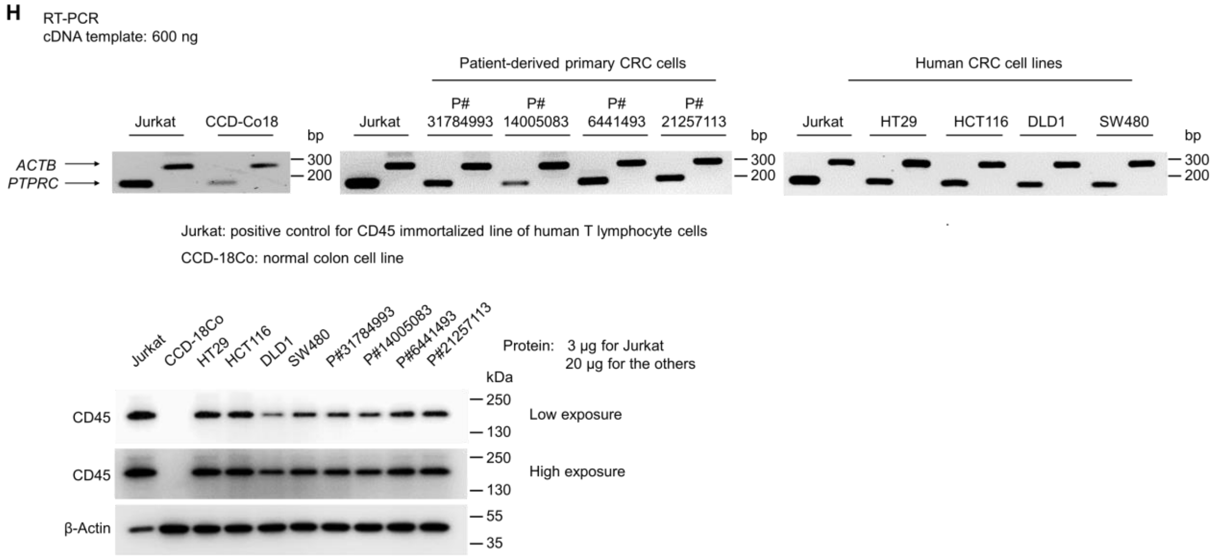
H
RT-PCR
cDNA template: 600 ng
Patient-derived primary CRC cells
Human CRC cell lines
Jurkat
CCD-Co18
bp
Jurkat
P# 31784993
P# 14005083
P# 6441493
P# 21257113
bp
Jurkat
HT29
HCT116
DLD1
SW480
bp
ACTB
PTPRC
300
200
Jurkat: positive control for CD45 immortalized line of human T lymphocyte cells
CCD-18Co: normal colon cell line
Jurkat
CCD-18Co
HT29
HCT116
DLD1
SW480
P#31784993
P#14005083
P#6441493
P#21257113
Protein: 3 μg for Jurkat
20 μg for the others
kDa
CD45
Low exposure
CD45
High exposure
β-Actin
250
130
55
35

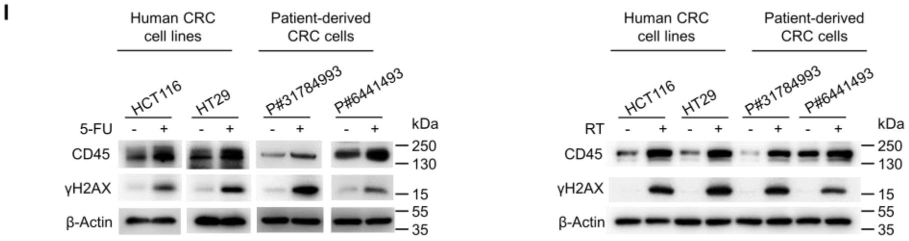
I
Human CRC cell lines
Patient-derived CRC cells
HCT116
HT29
P#31784993
P#6441493
5-FU
RT
kDa
CD45
γH2AX
β-Actin
250
130
15
55
35

Figure S1 (related to Figure 1).

(A) Immunohistological validation of CRC cells isolated from primary tumors from patients with CRC (P#31784993, P#14005083, P#6441493, P#21257113, P#22611293, and P#31815783). CRC epithelial cells were isolated using a Tumor Cell Isolation Kit (#130-108-339, Miltenyl Biotec, USA) with modifications. Briefly, we isolated epithelial CRC cells through positive selection of EpCAM+ cells using an anti-EpCAM antibody (#130-111-000, Miltenyl Biotec) and a magnetic separation column (#130-090-544, Miltenyl Biotec). HCT116 cells were used as a positive control for the CRC epithelial phenotype (EpCAM+, Vimentin-, CK7-), and MRC5 cells were used as a positive control for the fibroblast phenotype (EpCAM- Vimentin+, CK7-). (B) The scRNA-seq data of CRC tumors were obtained from the GEO web server (GSE81861). Comparison of multiple hematopoietic lineage marker expressions between in CD45-expressing epithelial cancer cells and non-expressing counterparts. (C) Triple-staining FACS analyses were performed in CRC tumor tissues as follows: (i) CD3, EpCAM, and CD45; (ii) CD4, EpCAM, and CD45; and (iii) CD8, EpCAM, and CD45. The dot blot shows the percentage of the indicated cellular population in tissues from patients with CRC (P#20051910, P#20051914, P#20052910, and P#20052914). (D) FACS plots show higher CD45 protein levels in CRC epithelial cells than in matched normal colorectal epithelial cells. Primary cells were isolated independently from normal or cancerous tissues obtained from patients with CRC (P#31325173, P#31701313, P#27423233). The total isolated cells were subjected to FACS analysis. Single cells were selected through sequential gating strategies, including scatter plots, SSC gates, and FSC gates. Then, live cells were gated through PI staining. These live cells were stained with antibodies against EpCAM (#53-8326-41, Thermo Fisher Scientific, NJ, USA) and CD45 (#555485, BD Pharmingen, CA, USA). EpCAM and CD45 expression patterns were plotted and

compared to those of cells stained with isotype control antibodies. (E) The CD45 protein expression pattern in CRC epithelial cells (EpCAM+) was visualized using immunofluorescence staining of formalin-fixed paraffin-embedded primary tumors from patients with CRC. Red, green, and blue indicate CD45 (#8216, Abcam, MA, USA), EpCAM (#93790, Cell Signaling Technology, MA, USA) and nuclei, respectively. Membrane expression of CD45 was detected in some CRC epithelial cells and displayed as a yellow signal in the merged images. H&E counterstaining was used to distinguish normal and tumor regions. (F) Primary cells were isolated from the normal intestine of wild-type mice or from adenomatous intestinal polyps of $APC^{Min/+}$ mice (n = 4 animals/group). Singe cells were gated as described in (D). Live cells were gated through Annexin V staining. Then, these live cells were stained with antibodies against EpCAM (#347198, BD Pharmingen) and CD45 (#555485, BD Pharmingen). (G) The FACS plot shows the CD45 and EpCAM expression patterns in CRC epithelial cells isolated from patients' primary tumors using the Jurkat cell line, which is a positive control for CD45 expression but lacks EpCAM expression (n = 3/group). All tested patient-derived CRC cells (P#6441493, P#21257113, P#31784993, and P#14005083) expressed EpCAM, suggesting their epithelial origin, and included the CD45-expressing population. Moreover, FACS analyses of the colocalization of EpCAM with other leukocyte markers confirmed the absence of other leukocyte markers (CD3, CD4, CD8, CD25, CD56, and CD16) in all patient-derived CRC cells, suggesting that the CD45-expressing population identified among patient-derived CRC cells comprises epithelial cells distinct from hematopoietic lineage cells. (H) RT-qPCR analysis of CD45 (PTPRC) expression in various CRC cells. The bar graph shows the PTPRC transcript level as the fold change compared with that of CCD-18Co, a normal colonic epithelial cell line. CD45 protein levels were determined in the same cell lysate samples used in Figure 1G by

performing a Western blot analysis with different anti-CD45 antibodies (#ab8216). β-Actin served as a loading control. (I) Cells were treated with 5-fluorouracil (5-FU, 1 μM) and radiation (4 Gy). After 48 h, the surviving cells were subjected to Western blot analysis to determine CD45 protein levels (#10558, Abcam). γH2AX (#11175, Abcam) was used as a marker for DNA damage induced by 5-FU or radiation. Statistical analyses comparing two groups were performed using Student's t-test. *** indicates p-values <0.001.

Fig. S2

A

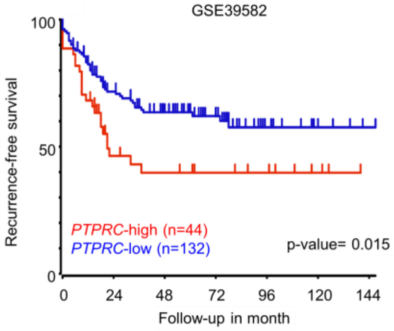

B

Clinicopathological characteristics of patients in the retrospective study

Residual tumor size	
mean	1.90±1.62 cm (range 0-5.0 cm)
Tumor grade	Low grade
Depth of tumor invasion (ypT)	
0	15 34.1%
1	0 8.0%
2	8 18.2%
3	20 45.5%
4	1 2.3%
Lymph node metastasis (ypN)	
0	35 79.5%
1	7 15.9%
2	2 4.5%
Post-therapeutic primary tumor stage (yStage)	
0	12 27.3%
I	9 20.5%
II	14 31.8%
III	5 11.4%
IV	4 9.1%
Post-therapeutic primary tumor regression grade (TRG)*	
TRG0	12 27.3%
TRG1	2 4.5%
TRG2	23 52.3%
TRG3	7 15.9%
CD45-expressing CRC epithelial cells	
Mean ratio 0.16096±0.24376 (range 0.0009-0.872804)	
Recurrence	
absent	31 70.5%
present	13 29.5%
Survival	
alive	32 72.7%
dead	12 27.3%
Follow-up duration	
mean	100±66.5 mos (range 5-237 mos)

***AJCC tumor regression grade (TRG)**

-TRG0: complete response; no viable cancer cells
-TRG1: near-complete response; single cells or rare small groups of cancer cells
-TRG2: partial response; residual cancer with evident tumor regression
-TRG3: poor response; extensive residual cancer

Figure S2 (related to Figure 2).

(A) Kaplan-Meier plots show the recurrence-free survival of 176 patients with colon cancer (GSE39582), according to the *PTPRC* expression levels in primary tumor tissues (p-value = 0.015, upper panel). RNA-sequencing data of colon cancer patients were obtained from public database, R2: Genomic analysis and visualization platform (https://hgserver1.amc.nl/cgi-bin/r2/main.cgi). (B) The clinicopathological characteristics of the patients (n = 44) in the retrospective study are summarized. CD45 expression in CRC cells was measured by performing immunofluorescence staining (#8216, Abcam) of pretreatment biopsy cancer tissues from patients with CRC who had received preoperative chemoradiotherapy (CRT). The results were scored based on the ratio of the colocalized area (EpCAM+CD45+) to the entire area of epithelial cancer cells (EpCAM+, #93790, Cell Signaling Technology) using image analysis software (Image-Pro Premier 9.0, Media Cybernetics, MD, USA). Three to five spots per sample were selected randomly and subjected to image analysis, and the mean value was calculated for each sample. The surgically resected tumors were pathologically diagnosed according to the World Health Organization classification scheme and classified according to the American Journal of Critical Care (AJCC) TNM system. Therapeutic responses to preoperative CRT were estimated by two pathologists according to the AJCC tumor regression grade (TRG).

Fig. S3 (continued)

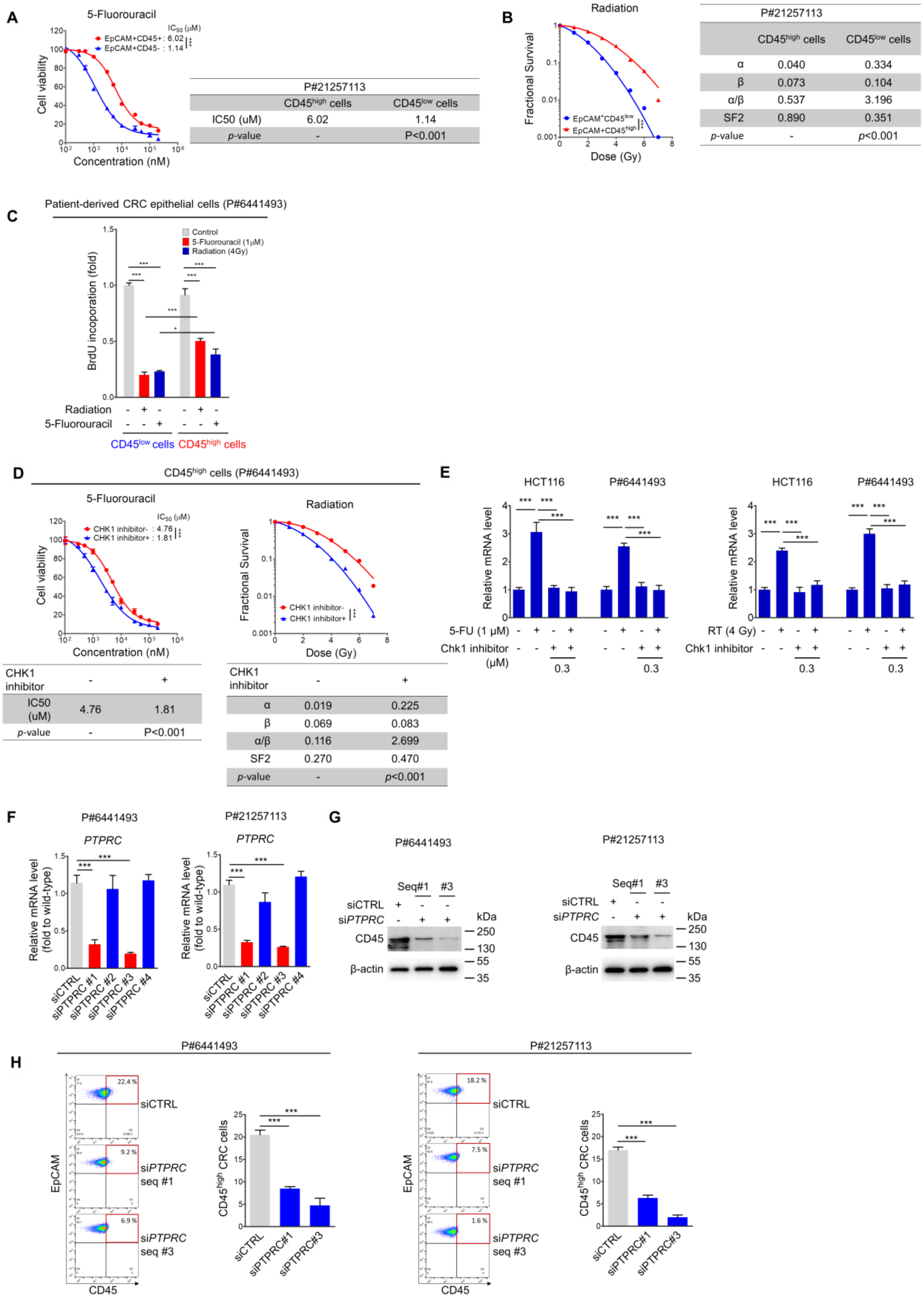

A (P#21257113)

	CD45high cells	CD45low cells
IC50 (uM)	6.02	1.14
p-value	-	P<0.001

B (P#21257113)

	CD45high cells	CD45low cells
α	0.040	0.334
β	0.073	0.104
α/β	0.537	3.196
SF2	0.890	0.351
p-value	-	*p*<0.001

D

CHK1 inhibitor	-	+
IC50 (uM)	4.76	1.81
p-value	-	P<0.001

CHK1 inhibitor	-	+
α	0.019	0.225
β	0.069	0.083
α/β	0.116	2.699
SF2	0.270	0.470
p-value	-	*p*<0.001

Fig. S3 (continued)

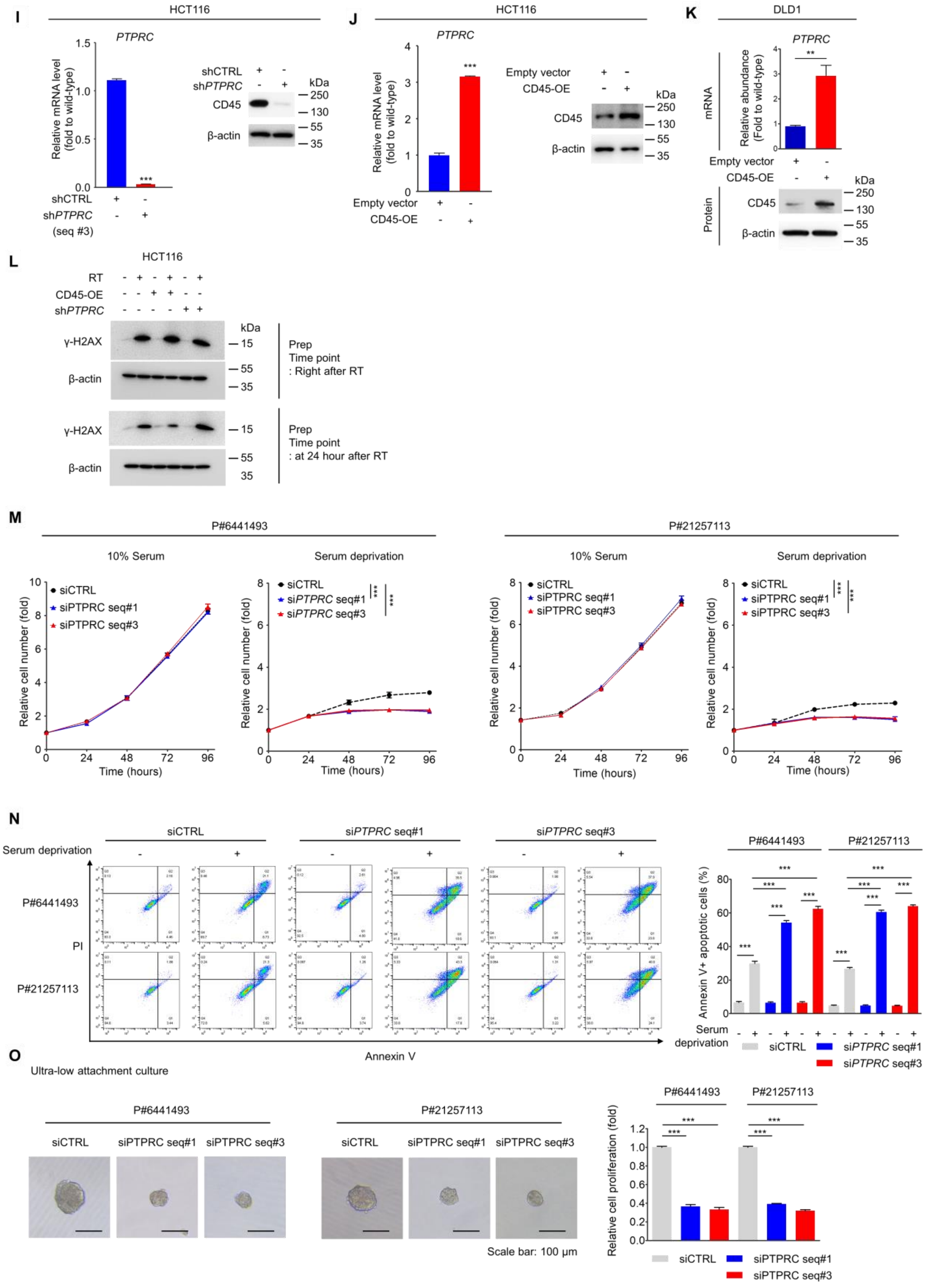

Fig. S3

P

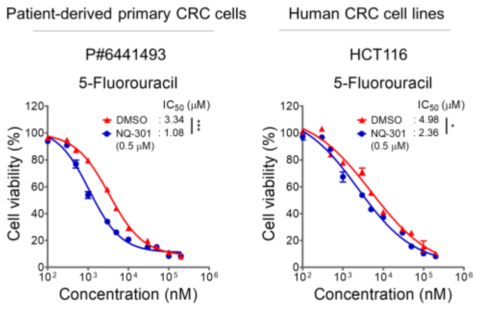

	P#644149		HCT116	
	DMSO	NQ-301	DMSO	NQ-301
IC50 (uM)	3.34	1.08	4.98	2.36
p-value	-	P<0.001	-	P<0.05

Q

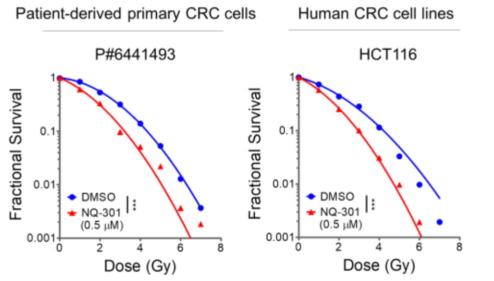

	P#6441493		HCT116	
	DMSO	NQ-301	DMSO	NQ-301
α	0.091	0.375	0.249	0.449
β	0.102	0.106	0.071	0.112
α/β	0.899	3.533	3.503	4.019
SF2	0.531	0.332	0.434	0.252
p-value	-	*p*<0.001	-	*p*<0.001

R

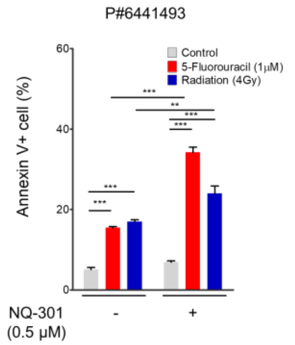

S

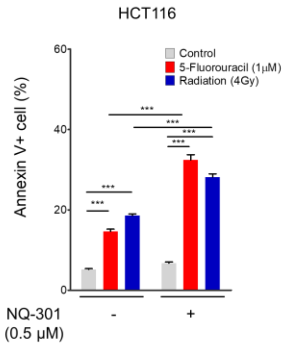

T

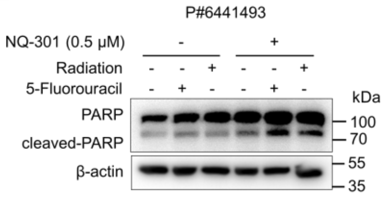

U

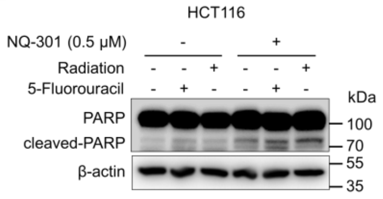

Figure S3 (related to Figure 3).

(A) Patient-derived primary CRC cells were separated into two groups according to the CD45 expression level ($CD45^{high}$ and $CD45^{low}$). The IC_{50} values of 5-FU were determined using an MTT assay (n = 5/group) and calculated using GraphPad Prism 5 software (GraphPad Software, Inc., CA, USA). (B) Radiation sensitivity was measured using traditional methods, in which the survival potential of irradiated cells was estimated with clonogenic assays, and the radiation biological parameters and statistical significance were then analyzed using a linear-quadratic model (n = 3/group). The parameters and statistical values were calculated using GraphPad Prism 5 software. (C) The extent of proliferating cells at 24 h after 5-FU or radiation treatment was measured by using Bromodeoxyuridine (BrdU) incorporation assay. (D) Reduction in the therapy resistance phenotype in $CD45^{high}$ cells (P#6441493) by interference with the DNA damage response via treatment with a CHK1 inhibitor (n = 5/group, left panel). The IC_{50} values of 5-FU were determined as described in (A) in the presence or absence of the CHK1 inhibitor (0.3 μM). Relative sensitivities of cells to radiation in the presence of absence of the CHK1 inhibitor were determined as described in (B). (E) *PTPRC* mRNA levels were determined using RT-qPCR after 5-FU or radiation treatment with or without CHK1 the inhibitor (0.3 μM, n = 3/group). (F) CD45 knockdown experiments were performed with siRNAs targeting the *PTPRC* gene (siPTPRC). At 24 h after siRNA transfection, total RNA was extracted and subjected to RT-qPCR. Significant reductions in *PTPRC* mRNA levels were determined using one-way ANOVA with Dunnett's multiple comparison tests (GraphPad Prism 5, n = 3/group). (G) Decreases in CD45 protein levels were confirmed using Western blotting. (H) FACS analysis validated the reductions in CD45 protein levels after CD45 knockdown (n = 3/group). Statistical significance was determined as described in (F). (I) The most potent siPTPRC sequence (#3) was cloned into

the pLKO-puro lentiviral vector to generate an shRNA plasmid (shPTPRC). The shPTPRC vector was transfected into the HCT116 cell line, and stable cells were generated by antibiotic selection. CD45 knockdown efficiency was confirmed using RT-qPCR and Western blot analyses (n = 3/group). (J) HCT116 and (K) DLD1 cells overexpressing (OE) CD45 were generated via lentiviral vector transfection (LV277909, abmGood, Vancouver, Canada). After antibiotic selection, CD45 overexpression was confirmed using RT-qPCR and Western blot analyses (n = 3/group). (L) Western blot analyses were performed immediately after or 24 h after radiation exposure using lysates from control, CD45-OE, and CD45-depleted HCT116 cells. DNA damage was analyzed by estimating the γH2AX levels in cell lysates. (M) Cell proliferation was compared between control and CD45-depleted CRC cells. Cells were transfected with the control (siCTRL) or siPTPRC and incubated with 10% fetal bovine serum (FBS) or harsh serum-limited media (0.1% FBS). Numbers of viable cells were counted every 24 h for 4 days (n = 3/group). (N) FACS analyses show the Annexin V+ apoptotic cells among siCTRL- or siPTPRC-transfected CRC cells at 96 h after serum deprivation (n = 3/group). (O) CRC cells were transfected with siCTRL or siPTPRC and then cultured under ultralow attachment conditions for 5 days. Surviving cells proliferated and formed spheres. The relative cell viability was determined using the resazurin-based Cell Titer Blue assay (Promega, WI, USA, n = 5/group). (P and Q) Relative sensitivity to 5-FU and radiation was compared between in NQ-301-treaed and control (DMSO) CRC cells (0.5 μM, 48 h). (P) The IC_{50} values of 5-FU were determined using an MTT assay as described in (A, n = 5/group). (Q) After DMSO or NQO-301 treatment (0.5 μM, 48 h), radiation sensitivity was measured as described in (B, n = 3/group). (R-U) The percentage of apoptotic cells at 24 h after 5-FU or radiation treatment was visualized (R and S) by performing Annexin V staining (n = 3/group) or (T and U) by visualizing the level of cleaved

PARP with or without NQ-301 treatment (0.5 μM, 24 h). Statistical analyses were performed using one-way ANOVA followed by Dunnett's multiple comparison tests for comparisons to the control group or Student's t-test for comparisons between two groups. ** and *** indicate $p<0.01$ and $p<0.001$, respectively.

Fig. S4 (continued).

A

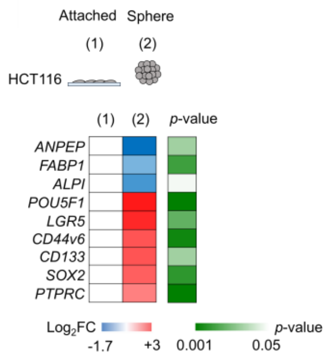

B

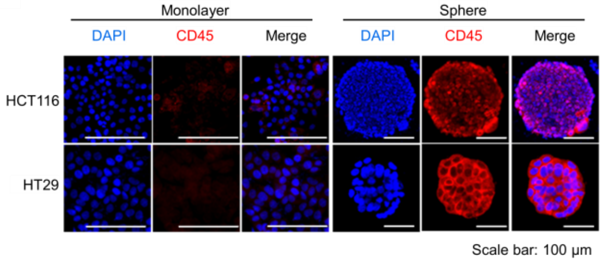

C

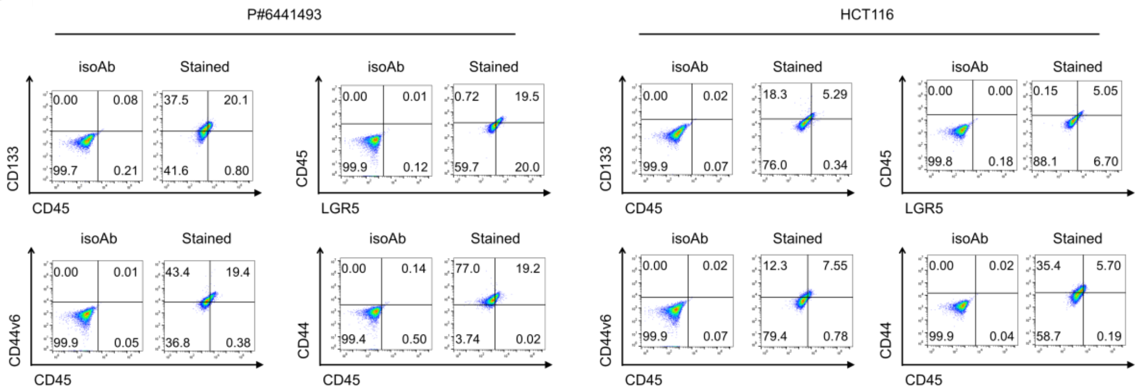

D

Cell no. of inoculation	1X10^5 CD45^high cells	1X10^5 CD45^low cells	5X10^4 CD45^high cells	5X10^4 CD45^low cells	1X10^4 CD45^high cells	1X10^4 CD45^low cells	5X10^3 CD45^high cells	5X10^3 CD45^low cells	*p*-value
	Frequency of Tumor formation (P#6441493)								
Day 7	0 % (0 of 8)	0 % (0 of 8)	0 % (0 of 8)	0 % (0 of 8)	0 % (0 of 8)	0 % (0 of 8)	0 % (0 of 8)	0 % (0 of 8)	-
Day 14	0 % (0 of 8)	0 % (0 of 8)	0 % (0 of 8)	0 % (0 of 8)	0 % (0 of 8)	0 % (0 of 8)	0 % (0 of 8)	0 % (0 of 8)	-
Day 16	75.2 % (6 of 8)	50.0 % (4 of 8)	62.5 % (5 of 8)	25.0 % (2 of 8)	25.0 % (2 of 8)	0 % (0 of 8)	0 % (0 of 8)	0 % (0 of 8)	$2.97X10^{-2}$
Day 18	87.5 % (7 of 8)	50.0 % (4 of 8)	87.5 % (7 of 8)	37.5 % (3 of 8)	37.5 % (3 of 8)	12.5 % (1 of 8)	12.5 % (1 of 8)	0 % (0 of 8)	$1.51X10^{-3}$
Day 20	100 % (8 of 8)	75.0 % (6 of 8)	100 % (8 of 8)	37.5 % (3 of 8)	50.0 % (4 of 8)	12.5 % (1 of 8)	25.0 % (2 of 8)	0 % (0 of 8)	$1.38X10^{-2}$
Day 22	100 % (8 of 8)	87.5 % (7 of 8)	100 % (8 of 8)	37.5 % (3 of 8)	62.5 % (5 of 8)	12.5 % (1 of 8)	37.5 % (3 of 8)	0 % (0 of 8)	$2.95X10^{-5}$
Day 24	100 % (8 of 8)	100 % (8 of 8)	100 % (8 of 8)	37.5 % (3 of 8)	75.0 % (6 of 8)	25.0 % (2 of 8)	50.0 % (4 of 8)	12.5 % (1 of 8)	$2.62X10^{-6}$
Day 26	100 % (8 of 8)	100 % (8 of 8)	100 % (8 of 8)	62.5 % (5 of 8)	75.0 % (6 of 8)	25.0 % (2 of 8)	50.0 % (4 of 8)	12.5 % (1 of 8)	$5.37X10^{-5}$
Day 28	100 % (8 of 8)	100 % (8 of 8)	100 % (8 of 8)	75.0 % (6 of 8)	75.0 % (6 of 8)	37.5 % (3 of 8)	62.5 % (5 of 8)	12.5 % (1 of 8)	$5.88X10^{-4}$

(Determined during monitoring period)

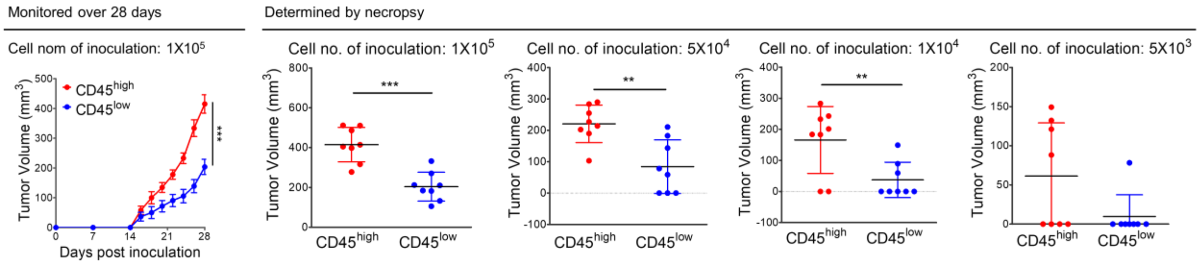

Fig. S4

E

Cell no. of inoculation	Frequency of Tumor formation (HCT116)								*p*-value
	$1X10^6$		$1X10^5$		$1X10^4$		$1X10^3$		
	$CD45^{high}$ cells	$CD45^{low}$ cells	$CD45^{high}$ cells	$CD45^{low}$ cells	$CD45^{high}$ cells	$CD45^{low}$ cells	$CD45^{high}$ cells	$CD45^{low}$ cells	
Day 7	0 % (0 of 8)	0 % (0 of 8)	0 % (0 of 8)	0 % (0 of 8)	0 % (0 of 8)	0 % (0 of 8)	0 % (0 of 8)	0 % (0 of 8)	-
Day 11	75.0 % (6 of 8)	50.0 % (4 of 8)	37.5 % (3 of 8)	25.0 % (2 of 8)	25.0 % (2 of 8)	0 % (0 of 8)	0 % (0 of 8)	0 % (0 of 8)	$7.20X10^{-2}$
Day 14	100.0 % (8 of 8)	62.5 % (5 of 8)	62.5 % (5 of 8)	37.5 % (3 of 8)	50.0 % (4 of 8)	12.5 % (1 of 8)	0 % (0 of 8)	0 % (0 of 8)	$1.10X10^{-5}$
Day 18	100.0 % (8 of 8)	75.0 % (6 of 8)	75.0 % (6 of 8)	50.0 % (4 of 8)	62.5 % (5 of 8)	25.0 % (2 of 8)	0 % (0 of 8)	0 % (0 of 8)	$8.30X10^{-6}$
Day 21	100 % (8 of 8)	100 % (8 of 8)	100.0 % (8 of 8)	75.0 % (6 of 8)	75.0 % (6 of 8)	25.0 % (3 of 8)	0 % (0 of 8)	0 % (0 of 8)	$2.06X10^{-3}$
Day 28	100 % (8 of 8)	100 % (8 of 8)	100.0 % (8 of 8)	87.5 % (7 of 8)	75.0 % (6 of 8)	37.5 % (3 of 8)	0 % (0 of 8)	0 % (0 of 8)	$1.29X10^{-2}$

(Determined during monitoring period)

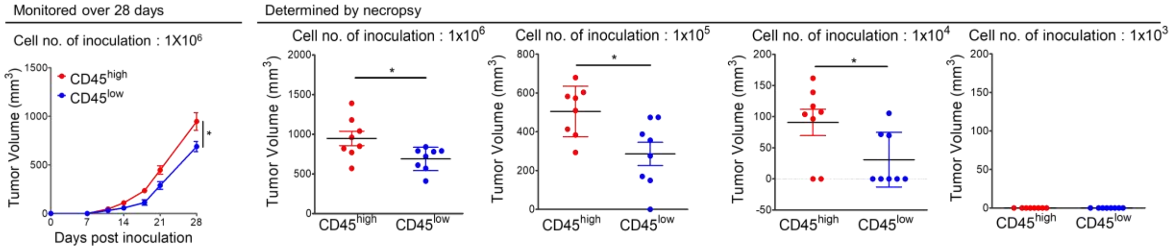

F

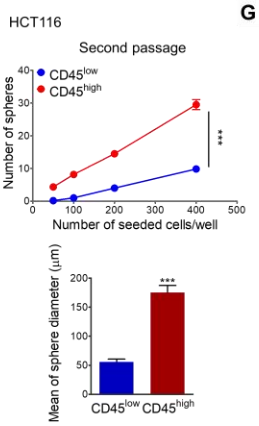

G

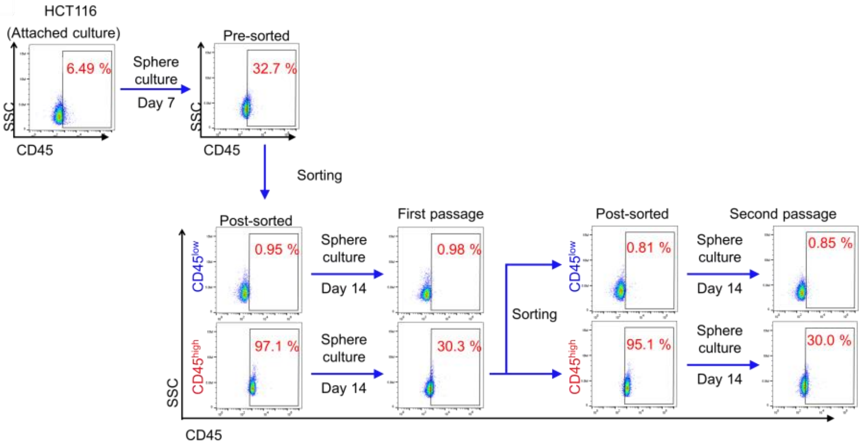

H

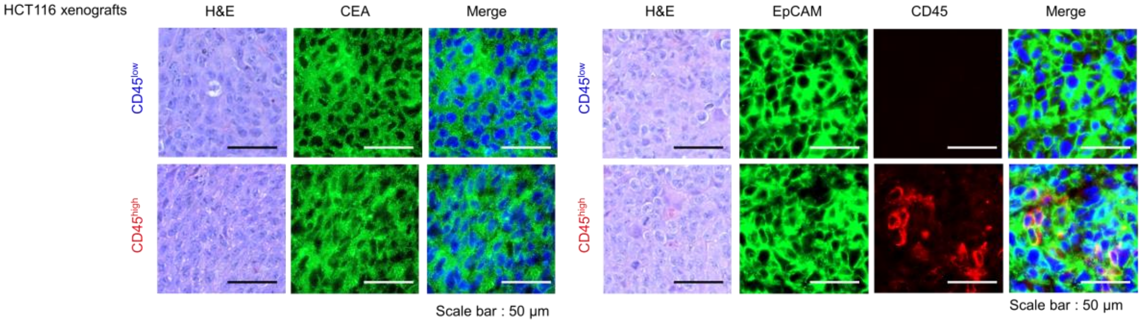

Scale bar : 50 μm

Scale bar : 50 μm

Fig. S4

I

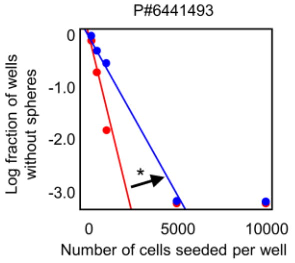

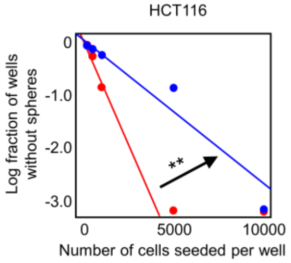

#	Group	Lower	Estimate	Upper
1	DMSO	1181	733	454
2	NQ301	2712	1673	1032

#	Group	Lower	Estimate	Upper
1	DMSO	2116	1293	790
2	NQ301	5821	3709	2364

Figure S4 (related to Figure 4).

(A) RT-qPCR shows an increase in the expression of CSC surface markers (*CD44v6*, *LGR5* and *CD133*) and stem cell-related transcription factors (*POU5F1* and *SOX2*) and a decrease in the expression of differentiation markers (*ANPEP*, *ALP*I, and *FABP1*) in CRC spheres, suggesting global trends of CSC enrichment in spheres compared with whole monolayer bulk cells (n = 3/group). (B) Immunofluorescence staining for CD45 in CSC-enriched spheres and in bulk cancer cells. (C) FACS images show the colocalization of CD45 with a panel of stem cell or CSC markers (LGR5, CD133, CD44, and CD44v6) in CRC cells. $CD45^{high}$ cells (%) among the indicated marker-positive or marker-negative populations are presented in Figure 4B. (D and E) The incidence of tumor-bearing mice was monitored for 28 days after the inoculation of (D) patient-derived CRC cells (P#6441493) or (E) HCT116 cells. The definitive tumor volume was determined by necropsy on day 28. Statistical significance of differences between two groups was determined using Student's t-test. *, ** and *** indicate $p<0.05$, $p<0.01$ and $p<0.001$, respectively. (F) Sphere-forming potentials were compared between $CD45^{high}$ and $CD45^{low}$ cells isolated from HCT116 spheres generated from $CD45^{high}$ cells, as shown in Figure 4H. Cells were seeded at varying cell densities and incubated under sphere culture conditions for 14 days, and then the number and size of spheres were counted (n = 6/group). (G) FACS plots show the increase in the $CD45^{high}$ population under sphere culture conditions from approximately 6% to 30%. HCT116 spheres were dissociated into single cells and divided into two groups according to the CD45 expression levels ($CD45^{high}$ and $CD45^{low}$ cells). These cells were seeded and incubated under sphere culture conditions for 14 days to generate spheres (the first-passage spheres). These first-passage spheres were dissociated into single cells and subjected to FACS to analyze CD45 expression levels and then proceeded to a second-passage sphere formation assay.

The first-passage spheres from the $CD45^{high}$ population consisted of both $CD45^{high}$ and $CD45^{low}$ populations with a ratio similar to that of the initial $CD45^{high}$ and $CD45^{low}$ populations (approximately 30%), while the spheres generated from the $CD45^{low}$ population consisted of only a $CD45^{low}$ population. Consistently, in the second-passage spheres, the generation of $CD45^{low}$ cells from a $CD45^{high}$ population was confirmed with a ratio similar to that of the initial $CD45^{high}$ and $CD45^{low}$ cells. (H) Immunohistological staining of CD45 expression levels in HCT116 xenograft tissues generated from $CD45^{high}$ and $CD45^{low}$ populations (Figure 4E). In this experiment, carcinoembryonic antigen (CEA), a CRC marker, was used to identify CRC cells in tissues. EpCAM was used to validate their epithelial origin. (I) *In vitro* limiting dilution assays were performed to compare the sphere-forming potential between NQ-301-treated (0.5 μM, 14 days) or control (DMSO) CRC cells (n = 12/group). Statistical analyses comparing differences between two groups were performed using Student's t-test. *, ** and *** indicate p-values <0.05, <0.01 and <0.001, respectively.

Fig. S5

A

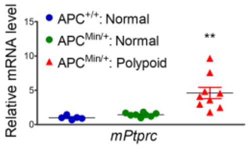

B

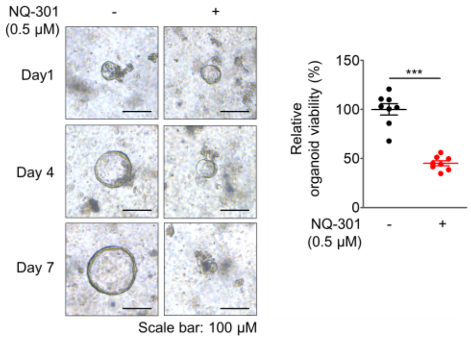

C

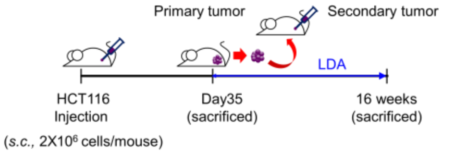

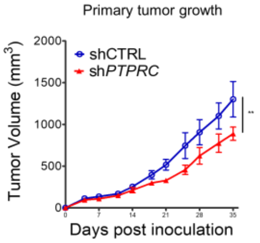

Secondary tumor incidence

Cell no. of inoculation	Frequency of Tumor formation (HCT116) 5X10^5 shCTRL	5X10^5 shPTPRC	1X10^5 shCTRL	1X10^5 shPTPRC	1X10^4 shCTRL	1X10^4 shPTPRC	1X10^3 shCTRL	1X10^3 shPTPRC	*p*-value
Day 0	0 % (0 of 8)	0 % (0 of 8)	0 % (0 of 8)	0 % (0 of 8)	0 % (0 of 8)	0 % (0 of 8)	0 % (0 of 8)	0 % (0 of 8)	-
Day 14	12.5 % (1 of 8)	0 % (0 of 8)	0 % (0 of 8)	0 % (0 of 8)	0 % (0 of 8)	0 % (0 of 8)	0 % (0 of 8)	0 % (0 of 8)	2.30X10^{-1}
Day 28	25.0 % (2 of 8)	12.5 % (1 of 8)	0 % (0 of 8)	0 % (0 of 8)	0 % (0 of 8)	0 % (0 of 8)	0 % (0 of 8)	0 % (0 of 8)	5.27X10^{-1}
Day 42	50 % (4 of 8)	37.5 % (3 of 8)	25.0 % (2 of 8)	12.5 % (1 of 8)	12.5 % (1 of 8)	0 % (0 of 8)	0 % (0 of 8)	0 % (0 of 8)	2.85X10^{-1}
Day 56	62.5 % (5 of 8)	50.0 % (4 of 8)	37.5 % (3 of 8)	12.5 % (1 of 8)	12.5 % (1 of 8)	0 % (0 of 8)	0 % (0 of 8)	0 % (0 of 8)	1.84X10^{-1}
Day 70	75.0 % (6 of 8)	50.0 % (4 of 8)	50.0 % (4 of 8)	25.0 % (2 of 8)	25.0 % (2 of 8)	12.5 % (1 of 8)	0 % (0 of 8)	0 % (0 of 8)	8.09X10^{-2}
Day 84	87.5 % (7 of 8)	62.5 % (5 of 8)	50.0 % (4 of 8)	25.0 % (2 of 8)	25.0 % (2 of 8)	12.5 % (1 of 8)	12.5 % (1 of 8)	0 % (0 of 8)	3.21X10^{-2}
Day 98	87.5 % (7 of 8)	76.0 % (6 of 8)	62.5 % (5 of 8)	25.0 % (2 of 8)	37.5 % (3 of 8)	12.5 % (1 of 8)	12.5 % (1 of 8)	0 % (0 of 8)	2.65X10^{-2}
Day 112	87.5 % (7 of 8)	75.0 % (6 of 8)	62.5 % (5 of 8)	25.0 % (2 of 8)	37.5 % (3 of 8)	12.5 % (1 of 8)	12.5 % (1 of 8)	0 % (0 of 8)	2.65X10^{-2}

(Determined during monitoring period)

Secondary tumor growth

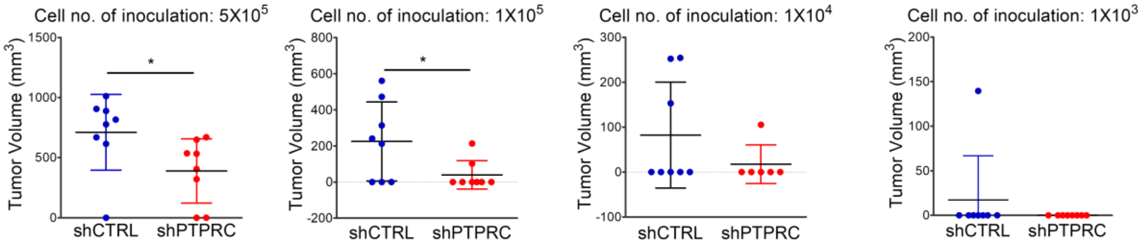

(Determined by necropsy)

Fig. S5

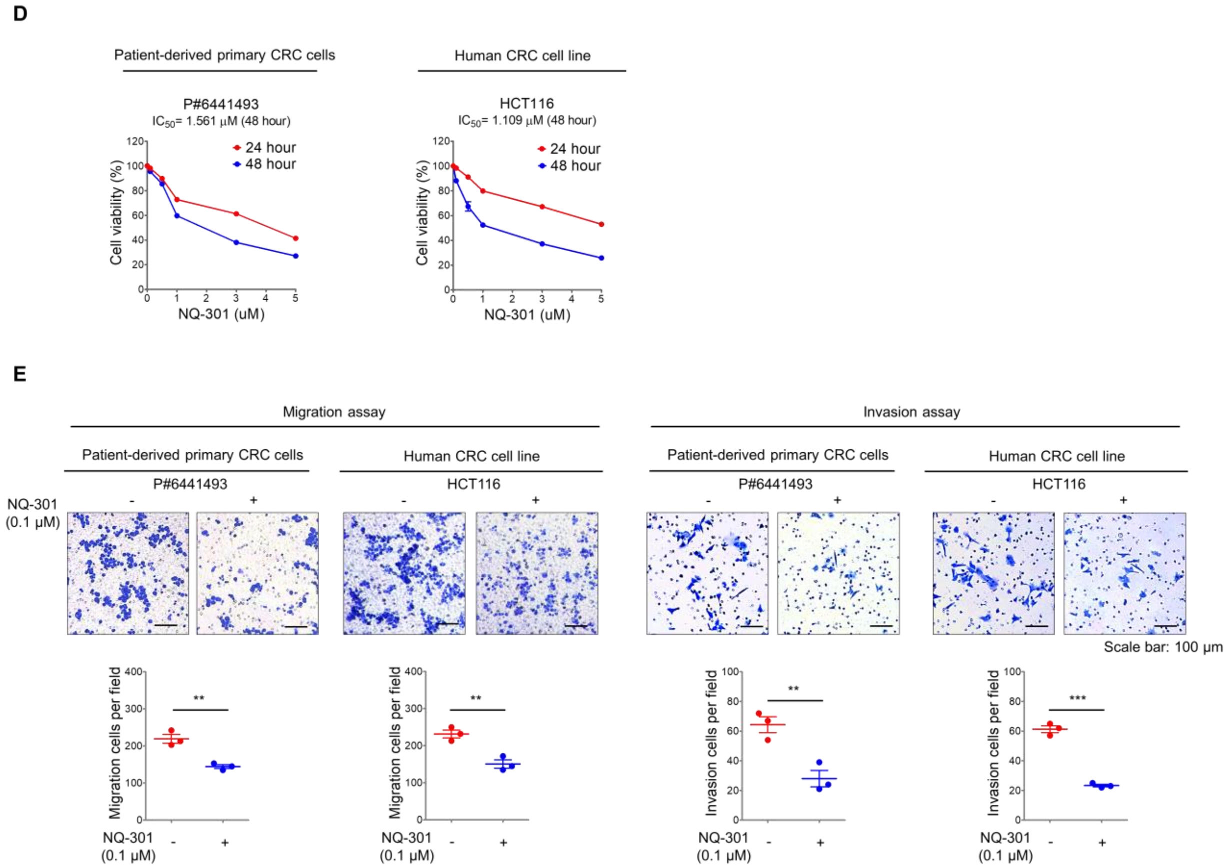

D
Patient-derived primary CRC cells
Human CRC cell line
P#6441493
IC$_{50}$= 1.561 μM (48 hour)
HCT116
IC$_{50}$= 1.109 μM (48 hour)
24 hour
48 hour
Cell viability (%)
NQ-301 (uM)
E
Migration assay
Invasion assay
Patient-derived primary CRC cells
Human CRC cell line
P#6441493
HCT116
NQ-301
(0.1 μM)
Scale bar: 100 μm
Migration cells per field
Invasion cells per field

Figure S5 (related to Figure 5).

(A) RT-qPCR analysis of gene expression in polypoid and normal intestines from $APC^{Min/+}$ mice relative to that in normal intestines from wild-type mice (n = 9 for $APC^{Min/+}$ mouse polypoid lesions, n = 8 for $APC^{Min/+}$ mouse normal intestines, and n = 5 for wild-type mouse normal intestines). (B) Brightfield images of $APC^{Min/+}$ mouse polyp-derived organoid cultures with or without NQ-301 treatment (0.5 μM). The viability of organoids was compared by performing a resazurin-based Cell Titer Blue assay on the 7th day of organoid culture (n = 8/group). (C) Primary tumor growth was monitored for 35 days after cell inoculation. On day 35, the mice were sacrificed, and the primary tumors were removed. Single tumor cells were isolated from the primary tumors by depleting mouse stromal cells with a mouse cell depletion kit (Miltenyl Biotec, Bergisch Gladbach, Germany), and then CRC cells were subjected to a limiting dilution assay to test their tumor-repopulating capability. The incidence of tumors in mice was monitored for 16 weeks and determined by definitive necropsy. The secondary tumor volume was compared between shCTRL and shPTPRC cells after necropsy. (D) Growth of CRC cells treated with NQ-301 and detected by MTT assays. Half maximal inhibitory concentrations (IC_{50}) of NQ-301 in patient-derived CRC cells (P#6441493) and HCT116 cells were 1.561 and 1.109 μM, respectively. (E) Transwell assays were performed to compare the migration (without matrigel coating) and invasion potentials (with matrigel coating) between NQ-301-treated (0.1 μM, 24 h) or control (DMSO) CRC cells (n = 3/group). Statistical analyses were performed using Student's t-test or the chi-square test. *, ** and *** indicate p-values <0.05, <0.01 and <0.001, respectively.

Fig. S6 (continued)

A

	Upstream regulator	Z-score	*p*-value
1	TGFB1	7.131	3.89E-64
2	IL1B	6.457	2.78E-18
3	TNF	6.082	9.03E-33
4	TP53	5.871	1.55E-51
5	IKBKB	5.849	3.81E-14
6	IL6	5.476	8.91E-13
7	CDKN2A	5.152	8.98E-19
8	P38 MAPK	4.929	8.91E-16
9	IFNG	4.796	4.93E-19
10	TWIST1	4.176	5.9E-15
11	SMARCA4	3.833	5.73E-21
12	OSM	3.72	3.22E-17
13	NFKBIA	3.719	2.04E-19
14	AGT	3.626	5.45E-16
**15**	**CTNNB1**	**3.603**	**5.39E-13**
16	SP1	3.530	9.32E-24
17	1APP	3.392	1.65E-19
18	RABL6	-5.775	4.06E-22
19	Estrogen receptor	-5.906	8.13E-17
20	MYC	-6.278	4.56E-26
21	Alpha catenin	-6.49	1.14E-15

B

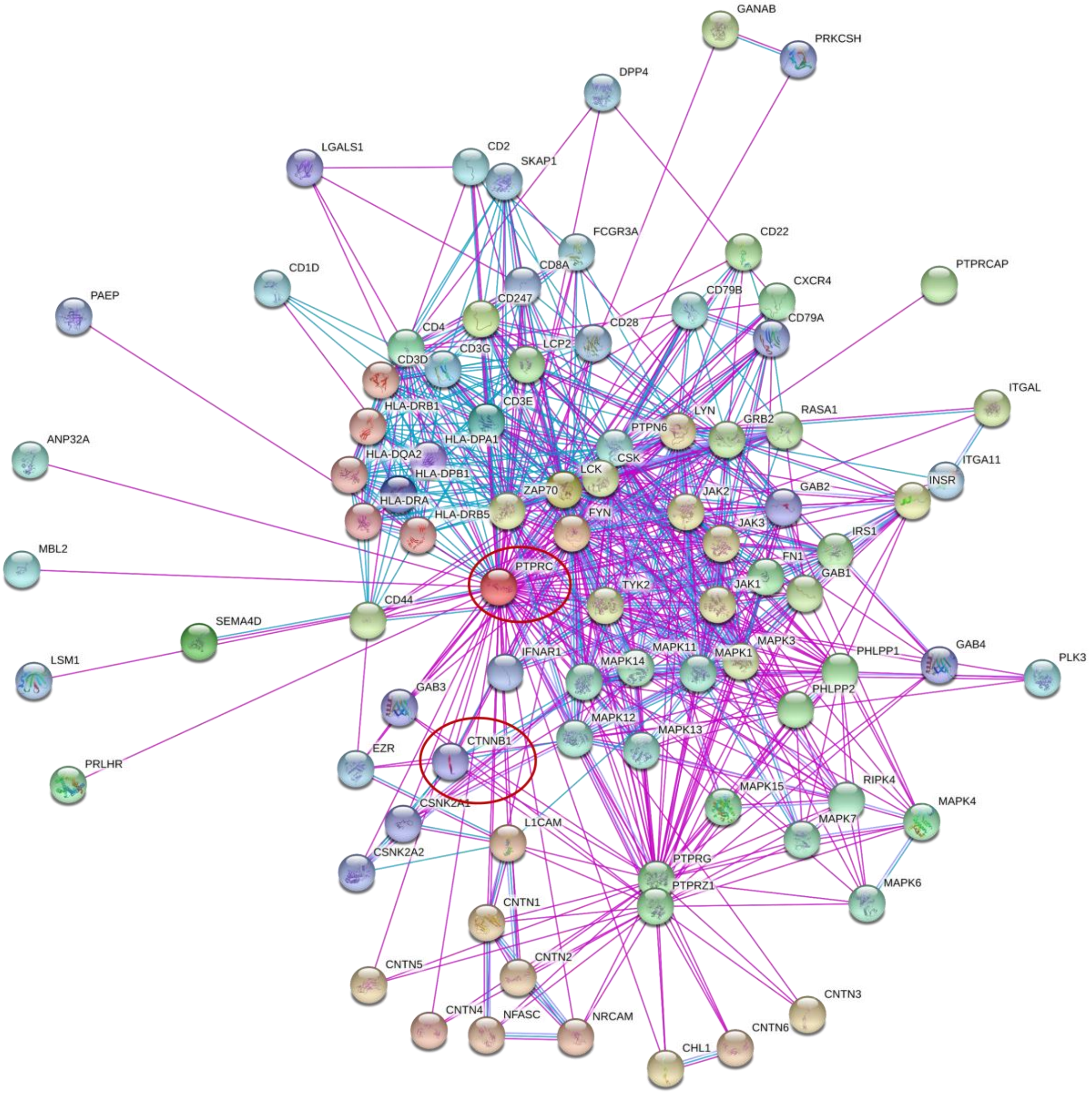

Fig. S6

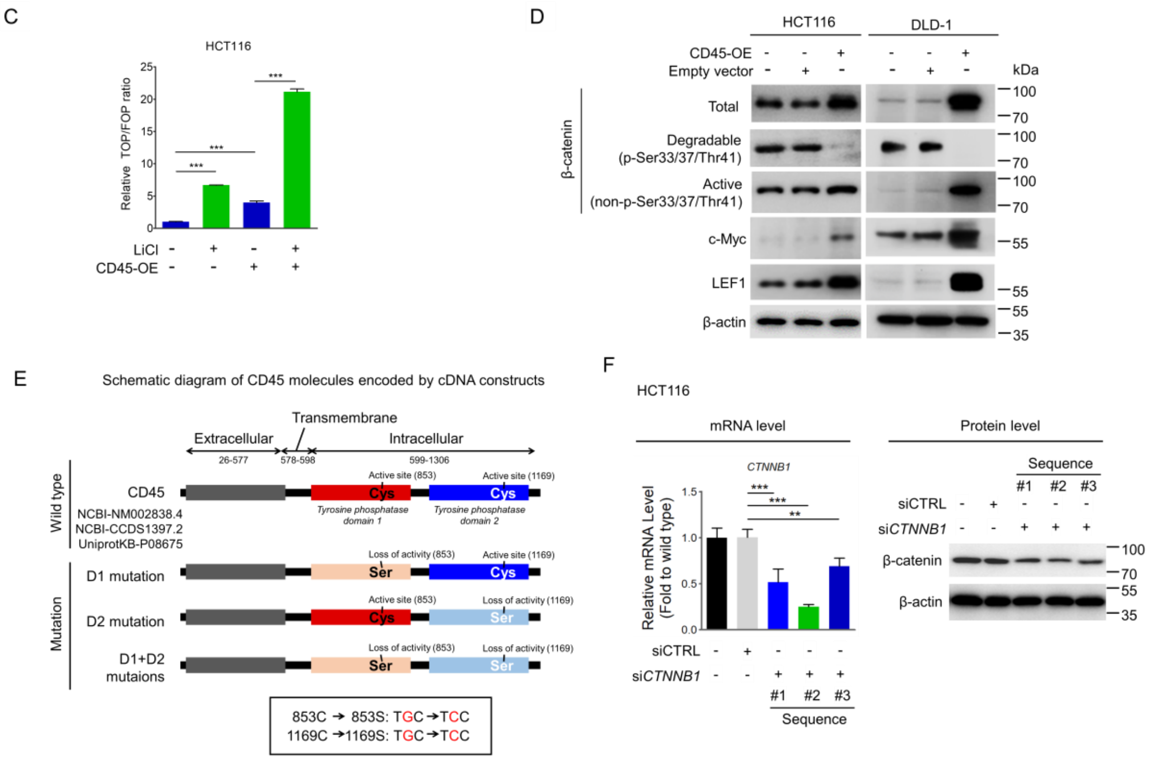

HCT116

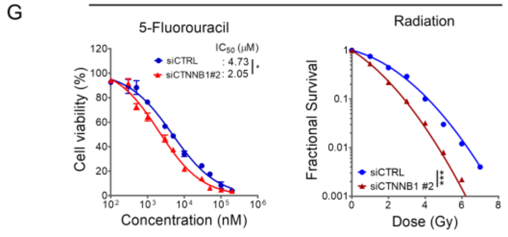

HCT116		
	shCTRL	si*CTNNB* *1*#3
IC50 (uM)	4.73	2.05
p-value	-	P<0.05

HCT116		
	siCTRL	si*CTNNB1*#2
α	0.218	0.556
β	0.081	0.089
α/β	2.682	6.237
SF2	0.432	0.22
p-value	-	*p*<0.001

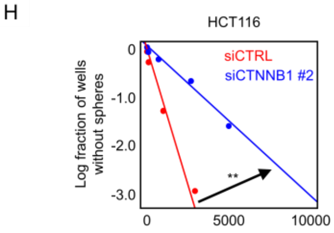

#	Group	Lower	Estimate	Upper
1	siCTRL	3201	1983	1042
2	si*CTNNB1* #2	5839	4203	2133

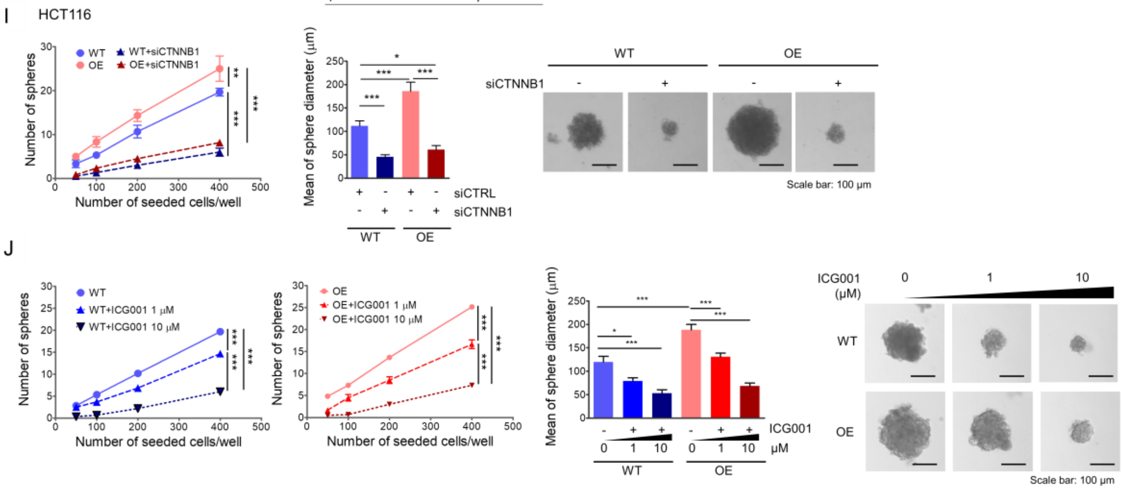

Figure S6 (related to Figure 6).

(A) Upstream regulators in residual tumors after CRT were predicted using IPA software based on the change in gene expression in tissues from patients with CRC after CRT (GSE15781) (cutoff: Z-score >3.3, Z-score <-3.3, and p-value <1E-12). (B) STRING, a protein interaction database, indicated a direct protein-protein interaction between β-catenin and CD45. (C) Effect of CD45 on the transcriptional activity of Wnt/β-catenin signaling in CD45-OE CRC cells. A TOP/FOP assay was performed in combination with Wnt activation (10 mM LiCl, 18 h, n = 3/group). (D) The total protein levels and phosphorylation status of β-catenin were determined in CD45-OE CRC cells using Western blotting. Wnt target proteins (c-Myc and LEF1) were also analyzed. (E) Schematic diagram of CD45 wild-type and mutant sequences showing the mutated sites in tyrosine phosphatase domains I and II, which disrupt CD45 phosphatase activity. (F) The efficiency of three siRNA sequences against CTNNB1 was determined using RT-qPCR, and the most potent sequence (#2) was selected for further experiments (n = 3/group). (G) Relative sensitivities to 5-FU and radiation were compared between β-catenin-knockdown and control CRC cells. The relative sensitivities to 5-FU were compared by determining the half-maximal inhibitory concentration (IC_{50}) values based on reductions in cell viability (left panel, n = 3/group). In addition, the sensitivity of the cells to radiation was measured using traditional methods; the survival potential of irradiated cells was estimated with clonogenic assays, and the radiation-related biological parameters and statistical significance were then analyzed using a linear-quadratic model (right panel, n = 3/group). Statistically significant differences between the two groups were calculated using GraphPad Prism software version 5. (H) An *in vitro* limiting dilution assay was performed to compare the sphere-forming potential between β-catenin-knockdown and control CRC cells (n = 12/group). (I) A sphere-forming assay was conducted to

examine the effect of β-catenin knockdown on CD45-induced stemness. Wild-type or CD45-OE cells were seeded at varying cell densities after siCTRL or siCTNNB1 transfection and incubated under sphere culture conditions for 14 days. Then, the number and size of spheres were counted (n = 6/group). (J) A sphere-forming assay was conducted to confirm the involvement of β-catenin in CD45-induced stemness using ICG001, an inhibitor of β-catenin–mediated transcription. EV-transfected or CD45-OE cells were seeded at varying cell densities and incubated under sphere culture conditions with or without ICG001 (1 or 10 μM) for 14 days. Then, the number and size of spheres were counted (n = 6/group). Statistical analyses comparing results with the control group were performed using one-way ANOVA followed by Dunnett's multiple comparison tests or Student's t-test for comparisons between two groups. *, ** and *** indicate p-values <0.05, <0.01 and <0.001, respectively.

Fig. S7

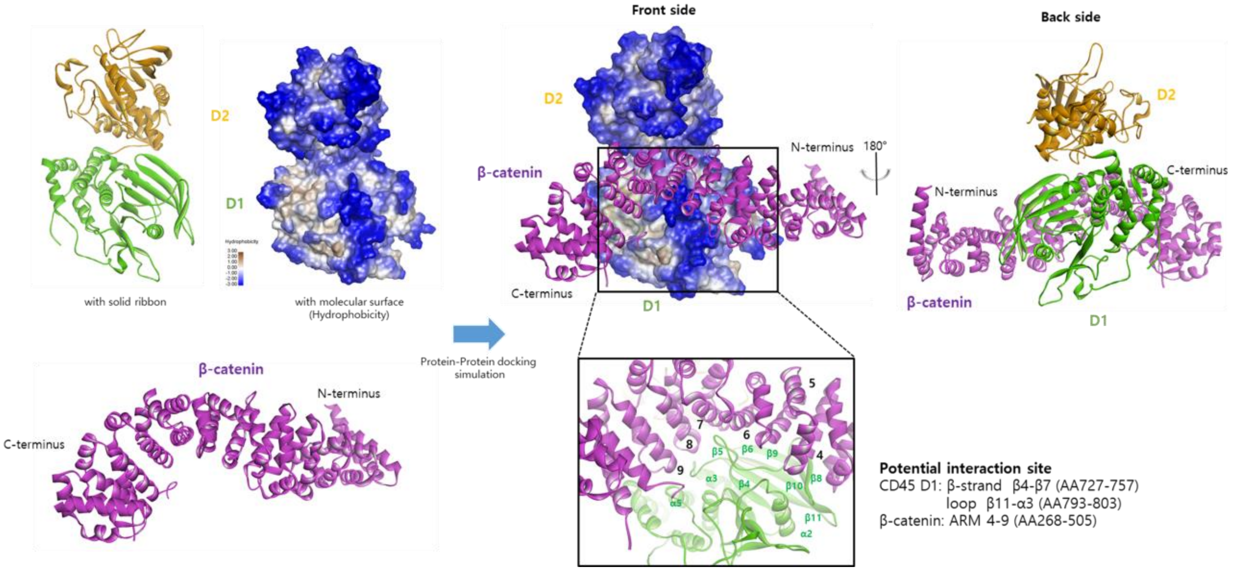

Figure S7 (related to Discussion).

Protein-protein interaction of CD45 and β-catenin was simulated. The result showed that the phosphatase domain D1 of CD45 is more favorable for interaction of ARM domain of β-catenin. Structure are represented in solid ribbon. The domain D1 and D2 of CD45 are colored in green and orange. The β-catenin is in magenta. Molecular surfaces of CD45 are depicted in brown and blue with color intensity increasing with the module of positive and negative values of molecular hydrophobicity potential, respectively.

Table S1. DEG list for residual tissues after CRT and metastatic tissues from patients with CRC

Attached at the end of this file.

Table S2. Clinical information for samples from patients with CRC used in this study

Primary tumor cells from tissues obtained from patients with CRC

Patient ID	Sex	Age	Diagnosis	Surgical staging (Stage)	Pathological diagnosis			
					K-ras	EGFR	p53	MS
P#31784993	M	67	Rectal cancer	T3N0M0	Wild-type	Mutation	Positive	MSS
P#14005083	M	45	Perforated S colon cancer with liver and lung metastasis	T4aN2b M1	Wild-type	Mutation	Positive	MSS
P#6441493	M	58	Upper rectal cancer with lung metastasis	T4N2M1	Mutation	Mutation	Negative	MSS
P#21257113	M	84	Proximal a-colon cancer with liver metastasis	T3N1M1	Wild-type	Mutation	Positive	MSS
P#22611293	F	50	Sigmoid colon cancer	T3N2M0	Wild-type	Wild-type	Positive	MSS
P#31815783	F	75	Sigmoid colon cancer	T3N0M0	Wild-type	Wild-type	Positive	Low
P#31325173	M	57	Rectal cancer with multiple liver metastases	T4aN2a M1b	Wild-type	Wild-type	Positive	Low
P#31701313	F	43	Rectal cancer with liver metastasis and vaginal invasion	T4aN2b M1	Mutation	Wild-type	Negative	MSS

P#27423233	F	55	Rectal cancer	T3N1aM0	Wild-type	Wild-type	Negative	MSI
P#20051910	M	58	Right colon cancer	T3N1M0	Wild-type	Mutation	Positive	Low
P#20051914	M	68	Right colon cancer	T2N0M0	Wild-type	Mutation	Negative	MSS
P#20052910	F	64	Sigmoid colon cancer	T4aN2bM0	Mutation	Mutation	Positive	MSS
P#20052914	F	49	Rectal cancer	T1N0M0	Mutation	Mutation	Negative	MSS

For immunohistological analysis

Patient ID	Sex	Age	Tumor site	Histological diagnosis	Histological grade	Lympho-vascular invasion	Peri-neural invasion	Tumor budding	Resection margin	pT stage	pN stage	Positive LNs	Total LNs
P#177936	M	66	Sigmoid colon	Adenocarcinoma	Moderately diff.	Yes	Yes	Yes	No	pT3	pN1b	3	22
P#177318	F	72	Sigmoid colon	Adenocarcinoma	Moderately diff.	No	Yes	No	No	pT3	pN2a	4	15
P#176870	F	69	Sigmoid colon	Adenocarcinoma	Moderately diff.	No	Yes	Yes	No	pT3	pN1b	2	17

MS, microsatellite; MSS, microsatellite stable; Low, low level of microsatellite instability; MSI, microsatellite instable; pT, pathological T stage; pN, pathological N stage; LN, lymph node metastasis; diff., differentiated

Table S3. Antibodies used for FACS, immunofluorescence staining, Western blotting, and immunoprecipitation (IP) analyses

Target	Type	Conjugation	Company	Cat #
For FACS analysis				
		Primary antibodies		
CD45	mMs	APC	BD Pharmingen™	555485
CD45	mRat	APC	BD Pharmingen™	561018
CD44	mRb	PE	Cell Signaling Technology	#2978
CD44	mRb	APC	Cell Signaling Technology	#2230
EpCAM	mMs	PE	BD Pharmingen™	347198
EpCAM	mMs	Alexa488	Thermo Fisher Scientific	53-8326-41
EpCAM	mRat	PE	Thermo Fisher Scientific	12-5791-82
CD133	mMs	PE	MACS	130-080-801
LGR5	mMs	APC	R&D SYSTEMS	FAB8078A
CD44v6	mMs	PE	R&D SYSTEMS	FAB3660P
CD25	mMs	FITC	BD Pharmingen™	555431
CD3	mMs	FITC	BD Pharmingen™	555339
CD3	mMs	FITC	eBioscience™	11-0037-42
CD4	mMs	FITC	BD Pharmingen™	555346
CD4	mMs	Alexa488	BD Pharmingen™	557667
CD8	mMs	FITC	BD Pharmingen™	561947
CD8	mMs	FITC	BD Pharmingen™	553031
CD56	mMs	Alexa488	BD Pharmingen™	557699
CD16	mMs	FITC	BD Pharmingen™	555406
For immunofluorescence				
		Primary antibodies		
EpCAM	mRb	-	Cell Signaling Technology	#93790
CD45	mMs	-	Abcam	ab8216

CD45	pRb	-	Abcam	ab10558
CD45	mRat	-	Abcam	ab25386
Vimentin	mMs	-	BD Pharmingen™	550513
CK7	mMs	-	DAKO	M7018
OLFM4	mRb	-	Cell Signaling Technology	#39141
LYZ1	mRb	-	Abcam	ab108508
DCLK1	pRb	-	Abcam	Ab31704
DCLK1	mMs	-	Santa Cruz Biotechnology	SC514584
CEA	mMs	-	Dako	GA62261-2
Secondary antibodies				
Alexa Fluor™ 405 goat anti-rabbit IgG (H+L)			Invitrogen	A31556
Alexa Fluor™ 488 goat anti-mouse IgG (H+L)			Invitrogen	A11001
Alexa Fluor™ 488 goat anti-rabbit IgG (H+L)			Invitrogen	A11008
Alexa Fluor™ 555 goat anti-rat IgG (H+L)			Invitrogen	A21434
Alexa Fluor™ 555 donkey anti-mouse IgG (H+L)			Invitrogen	A31570
Alexa Fluor™ 555 donkey anti-rabbit IgG (H+L)			Invitrogen	A31572
For Western blotting & IP analysis				
Primary antibodies				
CD45	pRb	-	Abcam	ab10558
Cyclin D1	mRb	-	Cell Signaling Technology	#2978
LEF1	mRb	-	Cell Signaling Technology	#2230
c-MYC	mRb	-	Abcam	ab32072
β-catenin (total)	pRb	-	Cell Signaling Technology	#9562
β-catenin (active)	mRb	-	Cell Signaling Technology	#8814
Phospho-β-catenin (Ser33/37/Thr41)	pRb	-	Cell Signaling Technology	#9561
Phospho-tyrosine	mMs	-	Cell Signaling Technology	#9411
β-actin	mMs	-	Sigma-Aldrich	A5316
Ub	mMs	-	Santa Cruz	sc-80017
PARP	mRb	-	Cell Signaling Technology	#9532

Caspase 3	pRb	-	Cell Signaling Technology	#9662
Cleaved-caspase 3	pRb	-	Cell Signaling Technology	#9661
γH2AX	pRb	-	Abcam	ab11174
		Secondary antibodies		
	HRP goat anti-mouse IgG		BD Pharmingen™	554002
	HRP goat anti-rabbit IgG		BD Pharmingen™	554021

m, monoclonal; p, polyclonal; Ms, mouse; Rb, rabbit

Table S4. Short interfering RNA (siRNA) sequences

	Sense	Antisense
**Human *PTPRC***		
#1	GUCAAGCUAAGGCGACAGA(dTdT)	UCUGUCGCCUUAGCUUGAC(dTdT)
#2	GUGUUGAACUCUCUGAGAU(dTdT)	AUCUCAGAGAGUUCAACAC(dTdT)
**#3**	**GAGAAAGGACGCAUGCUGU(dTdT)**	**ACAGCAUGCGUCCUUUCUC(dTdT)**
#4	**CUCUGAUGAUGACAGUGAU(dTdT)**	AUCACUGUCAUCAUCAGAG(dTdT)
**Mouse *Ptprc***		
#1	CUAUGAUCUGCGCAAGAAA(dTdT)	UUUCUUGCGCAGAUCAUAG(dTdT)
#2	GUUGAAAGGGAUGAUGAAA(dTdT)	UUUCAUCAUCCCUUUCAAC(dTdT)
#3	GUCACAGGGCAAACACCUA(dTdT)	UAGGUGUUUGCCCUGUGAC(dTdT)
**Human *CTNNB1***		
#1	CCUGGUGAAAAUGCUUGGU(dTdT)	ACCAAGCAUUUUCACCAGG(dTdT)
#2	CGUUCUCCUCAGAUGGUGU(dTdT)	ACACCAUCUGAGGAGAACG(dTdT)
#3	ACGACUAGUUCAGUUGCUU(dTdT)	AAGCAACUGAACUAGUCGU(dTdT)

*Bold sequences were used to generate shRNA plasmids.

Table S5. **List of primers used for RT-qPCR**

Human	Forward	Reverse
ALDH1A1	CAAATAGTGCACTGTCTCCAGG	ACGACACTACTTATTTGTAACACCT
ALPI	CCAGGACATCGCCACTCAG	TCAGTGCGGTTCCACACATA
ANPEP	CCACCTTGGACCAAAGTAAAGC	TCTCAGCGTCACCCGGTAGGA
BIRC5	CAAGGACCACCGCATCTCTAC	AGTCTGGCTCGTTCTCAGTGG
CCND1	TGTCGGTGTAGATGCACAGC	TGCATGTTCGTGGCCTCTAA
CD133	CAGAGTACAACGCCAAACCA	AAATCACGATGAGGGTCAGC
CD44	GGAGCAGCACTTCAGGAGGTTAC	GGAATGTGTCTTGGTCTCTGGTAGC
CD44v6	GGCAACTCCTAGTAGTACAACG	GTCTTCTTTGGGTGTTTGGC
CTNNB1	GAGCCTGCCATCTGTGCTCT	ACGCAAAGGTGCATGATTTG
DCLK1	TAGCCAGCGCCATCAAATAC	ACCCAGCTTCAGTGATTTGC
FABP1	TCACCTTCCAACTGAACCAC	GGAAGGATATCAAGGGGGTG
HOXD11	TCTCCGAGTCCTCGTGGGGA	GCAAAACACCAGCGCCTTCTA
KLF4	TTCTGGCAGTGTGGGTCATA	GAACTGACCAGGCACTACCG
KLF5	GGATGGAGGTGGGGTTAAAT	CCCTTGCACATACACAATGC
LEF1	AGCCTTCTTTTTCTGAGACAGC	GAACACCTTACAAGGGCGGA
LGR5	CTCAGCGTCTTCACCTCCTAC	TCTGCAGCATAAGAACTTTAAGAC
MCM3	CCAGTGTTCGGGCTGTAACT	GGCCACCTACATTGCAGAAG
MYC	CAAGTATACGTGGCAATGCGT	TCAAGAGTCCCAGGGAGAGT
NANOG	TGGGATTTACAGGCGTGAGC	AAGCAAAGCCTCCCAATCCC
POU5F1	GGGCTCTCCCATGCATTCAA	CACCTTCCCTCCAACCAGTT
PPIA	TGCCATCGCCAAGGAGTAG	TGCACAGACGGTCACTCAAA

PTPRC	TTCAGCCTGTTCCTTTGCTT	AGCACCTACCCTGCTCAGAA
SOX2	TCGGCAGACTGATTCAAATAATAC<br>AG	CCATGCAGGTTGACACCGTTG
SOX4	GAGAAACTGTGTGTGAGGGGA	AAAAAGCCTGCATGCAACAGA
SOX9	CATGAGCGAGGTGCACTCC	TCGCTTCAGGTCAGCCTTG
WSB1	GATCGTGGTTAGTTTGGG	TGGGTCACCAACTTGAG
Mouse	Forward	Reverse
Ptprc	ATGGTCCTCTGAATAAAGCCCA	TCAGCACTATTGGTAGGCTCC
Olfm4	CTGCTCCTGGAAGCTGTAGT	ACCTCCTTGGCCATAGCGAA
Lyz1	GAGACCGAAGCACCGACTATC	CGGTTTTGACATTGTGTTCGC
Dclk1	AGCGGAGAACCGCATTTCAA	ATCTCTGCCGAACGACATGG
Hprt	GCCTAAGATGAGCGCAAGTTG	TACTAGGCAGATGGCCACAGG

Supplementary materials and methods

Chemicals

Radioimmunoprecipitation assay (RIPA) buffer was prepared in our laboratory as previously reported [1]. The 10X cell lysis buffer (#9803), 3X SDS sample buffer (#7722), and Protein A agarose beads (#9863) were purchased from Cell Signaling Technology (Beverly, MA, USA). FACS Lysing™ Solution was purchased from BD Biosciences (San Diego, CA, USA). TRIzol reagent was purchased from Ambion (Austin, TX, USA). NQ-301 used in this study was kindly provided by Korea Chemical Bank (www.chembank.org) of Korea Research Institute of Chemical Technology.

Visualization of scRNA-seq data

The scRNA-seq data from CRC tumors were obtained from the GEO web server (GSE81861). Gene expression files quantified as fragments per kilobase per million reads (FPKM) were downloaded from GEO with the cell type identification file and applied to GraphPad Prism software version 9 to visualize the mRNA expression patterns of epithelial and leukocyte markers. In the cell type identification file, seven distinct cell clusters were determined by performing reference component analysis (RCA) using the global panel. The Seurat algorithm was used to generate a 2D plot of cell type clusters.

FACS analysis

FACS analysis was performed using a BD Accuri™ flow cytometer (BD Biosciences). FACS data were analyzed using FlowJo software (TreeStar, San Carlos, CA, USA) as described

in our previous report [1]. Cells were stained with specific antibodies according to the manufacturers' instructions. After a 1 h incubation at 4 °C, cells were washed with phosphate-buffered saline (PBS) and analyzed using a BD FACSCalibur system (BD Biosciences). The detailed antibody list is provided in Table S3.

Isolation of primary cells from normal intestines of wild-type mice and from adenomatous polyps of APC$^{Min/+}$ mice

Normal intestines and adenomatous intestinal polyps were surgically removed from wild-type and APC$^{Min/+}$ mice, respectively. We detached intestinal epithelial cells from the basement membrane using a previously reported EDTA/DTT protocol with slight modifications [2]. Briefly, intestinal tissues were incubated in dissociation buffer #1 (47 mL of DPBS, 3 mL of 0.5 M EDTA, and 75 µL of 1 M DTT) for 20 minutes on ice. The tissues were removed from dissociation buffer #1 and placed in dissociation buffer #2 (47 mL of DPBS and 3 mL of 0.5 M EDTA) and incubated for 10 minutes at 37 °C. After shaking, the intestinal epithelium was released from the basement membrane. Remnant tissues were removed, and epithelial cells were pelleted by centrifugation (1,000 × g for 5 min at 4 °C). Then, these epithelial clumps were incubated with collagenase III and dissociated into single cells using 100 µm nylon mesh. These single epithelial cells were subjected to FACS analyses. Live cells were gated based on Annexin V staining.

Comparison of relative sensitivity to 5-FU or radiation

Relative sensitivities to 5-FU were compared by determining the half-maximal inhibitory concentration (IC_{50}) values based on reductions in cell viability. Briefly, cells were seeded in 96-

well plates (5,000 cells/well) and incubated for 24 hours for attachment. Then, the cells were treated with 5-FU at various concentrations and incubated for 48 hours. Cell viability was measured by staining with thiazolyl blue tetrazolium bromide (MTT, Sigma-Aldrich, St. Louis, MO, USA), and the absorbance was measured using a microplate spectrophotometer (Bio-Tek Instruments Inc., Winooski, VT, USA). In addition, the sensitivity of the cells to radiation was measured using traditional methods [3]. Briefly, the survival potential of irradiated cells was estimated with clonogenic assays, and the radiation-related biological parameters and statistical significance were then analyzed using a linear-quadratic model (GraphPad Prism version 5, GraphPad Software, San Diego, CA, USA). Cell proliferation was measured using a fluorescence-based BrdU labeling and detection kit (Thermo Fisher Scientific) based on a 2-hour incorporation of BrdU.

Retrospective study population of rectal cancer for determining the correlation between marker expression and therapeutic response to preoperative chemoradiotherapy.

The clinical validation study set consisted of 44 pretreatment paraffin-embedded tissue samples obtained from patients with locally advanced distal rectal cancer (cT3-T4, N+) who had been treated at Chungnam National University Hospital. Clinicopathological characteristics are summarized in Supplementary Figure S2B. The cases had been previously reported [4]. Among the previously reported 93 cases, 53 cases with sufficient amount of cancer tissue were arranged in one tissue microarray block containing one representative tissue core of 2mm in diameter from each case. Out of the 53 cases, 9 cases were excluded from the analysis, since 5 cases were not available for tissue within the core, 2 cases showed no more cancer gland within the core, and the other two were poor quality of tissue with artifact. All cases were histologically proven low-

grade (well to moderately differentiated) adenocarcinomas and the patients had received preoperative chemoradiotherapy (CRT) consisting of 50.4 Gy of pelvic irradiation in 28 fractions, combined with 2 cycles of 5-fluorouracil (5-FU) (400 mg/m^2 per day) or capecitabine (1650 mg/m^2 per day) and leucovorin (20 mg/m^2 per day), followed by curative surgery average 6 weeks after completion of CRT. The surgically resected cases were pathologically diagnosed according to WHO classification [5], and were classified according to AJCC TNM system [6]. Therapeutic responses to preoperative CRT were estimated by two pathologists (J.M.K and H.J.C) according to AJCC tumor regression grade (TRG): complete response (TRG0); no viable cancer cells, near-complete response (TRG1); single cells or rare small groups of cancer cells, partial response (TRG2); residual cancer with evident tumor regression (partial response), poor response (TRG3); extensive residual cancer [7]. The patients were followed up for local recurrence or distant metastasis every 3 months for the first two postoperative years, and every 6–12 months thereafter. Mean follow-up duration was 100 months (range 5-237 months). Physical examination, serum carcinoembryonic antigen (CEA) assay, chest X-ray and abdominal ultrasound or CT scan were performed in every 6 months. Informed consent was obtained from each patient before they received preoperative CRT, and this retrospective study was approved by the Institutional Review Board of GIST.

CD45 expression levels in epithelial cells (EpCAM+) were visualized in pretreatment cancer tissues by immunofluorescence assays. Anti-EpCAM antibodies (cat# 93790, Cell Signaling Technology) were used at 1:200 dilution, and anti-CD45 antibodies (cat# ab8216, Abcam) were used at1:500 dilution. Alexa-555-anti-mouse IgG antibodies or Alexa488-anti-rabbit IgG antibodies were applied as 1:1000 dilution. 3 ~ 5 spots per each samples were randomly selected and applied to microscopic evaluation at total X 200 magnification. DAPI was

counterstained to visualize nuclei. The existence of CD45+ epithelial cells in primary tumors were scored based on the ratio of co-localized area (EpCAM+CD45+) within the epithelial cells (EpCAM+) using an image analysis software (Image Pro Premier 9.0, Media Cybermetics, MD, USA). H&E counterstaining was used for distinguishing normal and tumor region. Patients were divided in two groups by the mean value of CD45 expression levels in epithelial cells (mean ratio 0.16096 ± 0.24376; high: n = 16, low: n = 28). The immunofluoresent analysis was performed blindly without any information for therapeutic outcome.

The $\chi 2$-test and t-test or ANOVA test were performed to determine the correlation between marker expression and tumor regression grade, ypT, ypN or yStage, and survival curves were plotted using Kaplan-Meier method and compared using the log-rank test. Disease-specific survival (DSS) was defined as the time from the diagnosis date to rectal cancer related death. Recurrence free survival (RFS) was defined as the time from the operation date to any type of recurrence proven by CT, MRI or histology. Multivariate analyses with a Cox proportional hazard model using a forward conditional variable selection method was also performed with yStage (0-II vs. III-IV), TRG (0-1 vs. 2-3), CD45 expression level (low vs. high) as covariates. All results were considered statistically significant when P values were <0.05, and all analysis was performed using IBM SPSS 20 software for windows (IBM corp, Somers, New York, USA).

Gene expression modification by knockdown or overexpression

Cells were transfected with siRNAs targeting specific genes or a nonspecific negative control siRNA (Bioneer, Daejeon, Republic of Korea) in media (serum-, phenol-, antibiotic-free) with Lipofectamine^{TM} 2000 (Invitrogen) according to the manufacturer's instructions. Knockdown efficiency was confirmed by measuring mRNA expression using reverse

transcription PCR and RT-qPCR. The sequences of the siRNAs are listed in Table S4. After the most effective sequence was validated by RT-qPCR, that particular sequence was synthesized as short hairpin (sh) RNA and inserted into the pLKO.1 puro vector. The shRNA vectors were transformed into DH5α *E. coli* (Real Biotech Corporation, Banqiao City, Taipei, Taiwan). Then, the transformed cDNA was purified using the QIAGEN plasmid Maxi kit (QIAGEN, Hilden, Germany), transfected into 293FT cells (Invitrogen) using Lipofectamine 2000 as described above, and viral packaging mix (Sigma-Aldrich) was added. Viral supernatants were used for transfection. Cells were transfected with *PTPRC*-lentiviral vector (pLenti-GIII-CMV) (ABMgood, Richmond, CA, USA) using viral supernatants to establish CD45-OE cells. Empty vector-transfected cells were used as controls.

Immunofluorescence staining of cells

Proteins were visualized using the specific antibodies described in Table S3. Nuclei were counterstained with DAPI (Sigma-Aldrich). Secondary antibodies conjugated with fluorescent dyes were used to visualize target proteins. Alexa Fluor 555-conjugated anti-rabbit (Invitrogen) and Alexa Fluor 488-conjugated anti-mouse antibodies (Life Technologies, Carlsbad, CA, USA) were used. Fluorescence was visualized using Axio Imager 2 (Carl Zeiss, Oberkochen, Germany) (total magnification: 200X or 400X).

RNA isolation, reverse transcription PCR, and RT-qPCR

Total RNA was isolated from mouse tissues or cells using TRIzol reagent (Invitrogen). The purity of RNA was verified by measuring the 260/280 and 260/230 absorbance ratios. The cDNA templates were synthesized from 2 μg of total RNA using the PrimeScript^{TM} 1st strand

cDNA Synthesis Kit (Takara Biomedicals, Kusatsu, Japan) with random primers. Then, Power SYBR Green PCR Master Mix and Step-One Real-time PCR systems (Applied Biosystems, Foster City, CA, USA) were used for the PCR amplification of cDNAs. The primers are listed in Table S5.

Protein isolation and western blot analysis

Tissues or cells were homogenized in RIPA buffer for 20 min on ice. Protein concentrations were determined based on bicinchoninic acid (BCA) assay using the BCA Protein Assay kit (Thermo Fisher Scientific, Waltham, MA, USA). Proteins were denatured with sodium dodecyl sulfate (SDS) (Sigma-Aldrich) by boiling at 95 °C for 5 min. Equal amounts of total protein (4-15 μg) were electrophoresed on 8% or 10% acrylamide gels, and separated proteins were transferred to a polyvinylidene difluoride membrane (Millipore, Billerica, MA, USA). The membrane was blocked with 5% bovine serum albumin (Sigma-Aldrich) and incubated overnight at 4 °C with the indicated primary antibodies. The membrane was then incubated with horseradish peroxidase-conjugated secondary antibodies. Chemiluminescence of horseradish peroxidase was developed with ECL reagent (Atto, Tokyo, Japan) and detected with a digital imaging system (ProteinSimple, San Jose, CA). Antibodies used for Western blot analyses are listed in Table S3.

Apoptosis assay (Annexin V+)

The rate of cell apoptosis was quantitatively analyzed by performing apoptosis assays using an Annexin V-Fluorescein Isothiocyanate (FITC) Apoptosis Detection Kit I (BD Biosciences). Cell suspensions (1 x 10^6/mL) were prepared by washing cells twice with cold

PBS. Then, 100 μL of the suspension were transferred to a tube to which 5 μL of FITC, Annexin V, and propidium iodide (PI) were added. The mixture was incubated at room temperature for 15 min in the dark after gentle vortexing. After incubation, 400 μL of 1X binding buffer were added before analysis using flow cytometry.

Immunofluorescence staining of paraffin-embedded tissues

Formalin-fixed and paraffin-embedded sections of primary tumors were dewaxed and hydrated in an OTTIX bath (Diapath, Martinengo, Italy). The dewaxed slides were incubated with specific primary antibodies at 4 °C overnight. After washes with Tris-buffered saline containing 0.5% Tween-20 (TBST), slides were incubated with secondary antibodies at room temperature for 20 min. Alexa Fluor 488-conjugated anti-mouse antibodies (Cell Signaling) and Alexa Fluor 555-conjugated anti-rabbit antibodies (Cell Signaling) were used to visualize target proteins. Then, slides were washed with TBST, and the nuclei were counterstained with DAPI for 10 s. Slides were dehydrated using an OTTIX bath (Diapath) and mounted with mounting solution (Vector Laboratories, Burlingame, CA, USA). Fluorescence was visualized and analyzed using an LSM 510 META laser confocal microscopy system (Carl Zeiss). The dewaxed slides were stained with hematoxylin (Sigma-Aldrich) and eosin (Diapath) to histologically observe the morphology of tissues. Images were captured with a phase-contrast microscope (Carl Zeiss). Detailed information about the antibodies is provided in Table S3.

Luciferase reporter assays

A TOP-FOP luciferase assay was performed as described in our previous report to analyze the transcriptional activity of Wnt signaling [1]. CRC cells were seeded into 12-well

plates. The mixture of DNA (TOP or FOP), β-galactosidase, and Lipofectamine was prepared in the proportions of 1 μg:1 μg:2 μL in 50 μL of serum-free medium/well. Twenty-four hours after the mixed solution was applied to the seeded cells, cells were treated with LiCl, a Wnt-activating chemical. Cell lysis buffer was added after 17 h of LiCl treatment. Luciferase reagent was added to detect luciferase activity and detected using a luminometer (Promega, Madison, WI, USA, G3250) according to the manufacturer's recommendations. The relative Wnt transcriptional activity was determined by measuring luciferase activity and normalizing the raw results of TOP/FOP expression to β-galactosidase expression.

IP

IP was performed specifically for β-catenin to detect its ubiquitination. Cells were harvested by adding complete RIPA buffer to the plate. The supernatant of the sample was considered the cell lysate. The concentration of the cell lysate was adjusted to 1 μg/μL and then reacted with a β-catenin antibody (Cell Signaling) or normal rabbit IgG (Cell Signaling). Protein A agarose beads were added to each β-catenin antibody, normal rabbit IgG, and control sample (input) and bound by rotating at 4 °C. After the bead binding step, lysates bound to antibodies were centrifuged, and their pellets were resuspended and prepared for Western blotting. Mouse anti-rabbit IgG (light-chain specific) was used as the secondary antibody to detect β-catenin.

In vivo limiting dilution assay (LDA)

For the comparison of the tumor-initiating potential between $CD45^{high}$ and $CD45^{low}$ cells, patient-derived primary CRC cells or HCT116 cells were sorted into two groups according to the CD45 expression level by FACS ($CD45^{high}$ and $CD45^{low}$), and the cells were then subcutaneously

inoculated into NSG mice (NOD.Cg-Prkdc^{scid} Il2rg^{tm1Wjl}/SzJ, #005557, Jackson Laboratory, Bar Harbor, ME, USA) at various cell densities. After 28 days of observation, the frequency of tumor formation was monitored for 28 days and definitely determined by necropsy (n = 8 animals/group).

For the comparison of tumor-initiating potential between PTPRC knockdown and control HCT116 cells, cells were subcutaneously inoculated into NSG mice. On day 35, the mice were sacrificed, and the primary tumors were removed. Single tumor cells were isolated from the primary tumors by depleting mouse stromal cells with a mouse cell depletion kit (Miltenyl Biotec, Bergisch Gladbach, Germany), and then CRC cells were subjected to a limiting dilution assay to test their tumor-repopulating capability. The incidence of tumors in mice was monitored for 16 weeks and determined by definitive necropsy. LDA graphs were generated and statistical values were calculated using online software provided by Walter+Eliza Hall Bioinformatcis (http://bioinf.wehi.edu.au/software/elda/) as described in a previous report [8].

In vitro LDA and sphere-forming assays

For the *in vitro* sphere-forming assay, cells were seeded at varying cell densities and incubated under sphere culture conditions (poly-HEMA-coated 96-well plates; poly 2-hydroxyethyl methacrylate, Sigma Cat # P3932) for 14 days, and then the number of wells without spheres was counted (n = 12/group). The generation of LDA graphs and statistical calculations were performed using online software provided by Walter+Eliza Hall Bioinformatcis (http://bioinf.wehi.edu.au/software/elda/) as described in a previous report [8]. For the sphere formation assay, cells were seeded at varying cell densities and incubated under sphere culture conditions (poly-HEMA-coated 6-well plates) for 14 days, and then the number

and size of spheres were counted (n = 6/group).

Splenic injection mouse model

A splenic injection experiment was performed to estimate the step governing metastasis and distant organ colonization [9]. In this model, shCTRL- or shPTPRC-transfected HCT116-luc cells ($1X10^6$ cells/mouse) were inoculated into the spleen followed by splenectomy, and the surviving cells that grew in distant organs then contributed to the formation of liver metastases. We routinely monitored liver metastasis weekly by visualizing luciferase activity for 28 days (n = 9 for shCTRL, n = 8 for shPTPRC). After sacrifice, the livers were removed to determine liver metastasis.

$APC^{Min/+}$ mouse polyp-derived organoid culture

Single cells were isolated from the intestinal polyps of 20-week-old $APC^{Min/+}$ mice and cultured as described in a previous report with slight modifications [10]. Briefly, mouse intestines containing polyps were incubated with EDTA chelation buffer [10] for 60 min on ice. After chelation, the detached normal intestinal epithelial cells were removed by centrifugation, while tumor cells remained attached to the mesenchyme. Then, the intestinal fragments with tumor cells were dissociated with collagenase as described in a previous report [10]. The isolated tumor cells were counted and pelleted, and a total of 20,000 cells or 100 cells were then mixed with 50 µL or 10 µL of Matrigel (Corning® Matrigel® Growth Factor Reduced (GFR) Basement Membrane Matrix, #356231, Corning NY) and plated in 24-well plates or 96-well plates, respectively. After the polymerization of Matrigel, 500 mL of IntestiCult™ Organoid Growth Medium (Mouse, #06005, STEMCELL Technology) were added. Beginning on the day of

seeding, the growth and morphology of organoids were observed daily, and the viability of organoids was compared by performing a resazurin-based Cell Titer Blue assay (Promega, Leiden, The Netherlands) on the 7th day of organoid culture. For generation of the CD45 knockdown organoids, the isolated tumor cells were transfected with a small interfering RNA against the *PTPRC* gene using an NEPA21 superelectroporator (NEPAGENE, Chiba, Japan). Then, the transfected cells were cultured and monitored as described above. To test the effect of CD45 pharmacological inhibition on organoid growth, NQ-301 (0.5 μM) was treated on the 1st day after seeding, and measured the organoid viability on the 7th day of organoid culture as described above.

Migration and invasion assay

The Transwell system (8 μm pore size, Corning) was employed for migration and invasion assays. For migration assay, 3 x 10^5 cells were seeded on the upper chambers in serum-free medium with or without NQ-301 treatment (0.1 μM). And for invasion assay, 3 x 10^5 cells were seeded on the upper chamber of matrigel-coated Transwell system (8 μm pore size, Corning) in serum-free medium with or without NQ-301 treatment (0.1 μM). Then bottom chamber was filled with medium supplemented with 20% FBS. After incubation for 24 hours at 37 °C, the cells migrated or invaded through to the bottom of the insert membrane were fixed, stained with crystal violet and counted under observation with a phase-contrast microscope (Carl Zeiss, biological triplicates).

Protein-protein interaction docking simulation

The possible molecular interaction between the CD45 (PDB code: 1YGR) and β-catenin (PDB

code: 1G3J) was analyzed. Protein structure was prepared by adding missing residues, neutralization and energy minimization using Protein Preparation Wizard of Schrödinger program (Schrödinger, Inc., New York, NY). A protein-protein docking simulation was carried out using ZDOCK and ZRANK algorithms in Discovery Studio program (Accelrys, San Diego, CA). The 2000 interaction poses of CD45 and β-catenin based on electrostatic and shape complementarity were generated using ZDOCK and they were reranked by ZRNAK. The best-ranked pose in top cluster was chosen and was analyzed for protein-protein interactions of CD45 and β-catenin. The visualization of possible protein-protein interactions was performed by using Discovery Studio.

Supplementary references

1. Park SY, Kim JY, Choi JH, Kim JH, Lee CJ, Singh P*, et al.* Inhibition of LEF1-mediated DCLK1 by niclosamide attenuates colorectal cancer stemness. *Clin Cancer Res* **25,** 1415-1429 (2019)

2. Gracz AD, Puthoff BJ, Magness ST. Identification, isolation, and culture of intestinal epithelial stem cells from murine intestine. *Methods Mol Biol* **879,** 89-107 (2012)

3. Fertil B, Malaise EP. Intrinsic radiosensitivity of human cell lines is correlated with radioresponsiveness of human tumors: analysis of 101 published survival curves. *Int J Radiat Oncol Biol Phys* **11,** 1699-1707 (1985)

4. Kim JS, Kim JM, Liang ZL, Jang JY, Kim S, Huh GJ, *et al*. Prognostic Significance of Human Apurinic/Apyrimidinic Endonuclease (APE/Ref-1) Expression in Rectal Cancer Treated With Preoperative Radiochemotherapy. *Int J Radioation Oncology Biology Physics* **82**, 130-137 (2012)

5 Bosman FT, World Health Organization., International Agency for Research on Cancer.: WHO classification of tumours of the digestive system. 4th ed. Lyon: IARC Press, 2010.

6. Greene FL, American Joint Committee on Cancer., American Cancer Society.: AJCC cancer staging manual. 6th ed. New York: Springer, 2002

7. Amin MB, American Joint Committee on Cancer., American Cancer Society.: AJCC cancer staging manual. 8th ed. New York: Springer, 2017

8. Hu Y, Smyth GK. ELDA: extreme limiting dilution analysis for comparing depleted and enriched populations in stem cell and other assays. *J Immunol Methods* **347,** 70-78 (2009)

9. Bouvet M, Tsuji K, Yang M, Jiang P, Moossa AR, Hoffman RM. In vivo color-coded imaging of the interaction of colon cancer cells and splenocytes in the formation of liver

metastases. *Cancer Res* **66,** 11293-11297 (2006)

10. Xue X, Shah YM. In vitro organoid culture of primary mouse colon tumors. *J Vis Exp*, e50210 (2013).

Supplementary Table S1.

Up-regulated genes in metastatic colorectal cancer versus primary tumor (GSE68468, total 311 genes, P<0.0005, FC≥1.5) © 2000-2017 © 2000-2017 QIAGEN. All rights reserved.				
#	ID	Symbol	Expr Fold Change	Expr p-value
1	206155_at	ABCC2	1.618	0.000436
2	200045_at	ABCF1	3.159	0.00000719
3	207268_x_at	ABI2	2.667	4.55E-08
4	213102_at	ACTR3	1.502	0.00000176
5	222147_s_at	ACTR5	1.827	0.0000106
6	206833_s_at	ACYP2	2.013	0.00000003
7	217419_x_at	AGRN	1.706	0.0000176
8	220841_s_at	AHI1	2.914	1.54E-11
9	204664_at	ALPP	1.672	0.00000169
10	212211_at	ANKRD17	1.543	0.0000765
11	200782_at	ANXA5	20.576	3.28E-09
12	214341_at	AP1G2	1.705	0.0000774
13	214959_s_at	API5	1.928	0.00000182
14	213892_s_at	APRT	1.598	0.000378
15	215927_at	ARFGEF2	1.558	0.0000339
16	202207_at	ARL4C	1.621	6.64E-08
17	49111_at	ARRB1	1.59	0.000136
18	205894_at	ARSE	1.968	0.000000448
19	207284_s_at	ASPH	2.121	0.00000994
20	203168_at	ATF6B	1.538	0.000355
21	220237_at	ATG3	1.501	0.00000123
22	212280_x_at	ATG4B	1.727	2.55E-11
23	210205_at	B3GALT4	2.93	0.00000339
24	210535_at	B9D1	1.903	3.74E-08
25	205294_at	BAIAP2	1.662	0.00000122
26	208368_s_at	BRCA2	1.616	0.00029
27	215010_s_at	BRSK2	2.03	9.51E-11
28	214117_s_at	BTD	1.528	0.00000313
29	220152_at	C10orf95	6.246	1.99E-20
30	220601_at	C16orf70	2.925	0.000000903
31	218123_at	C21orf59	2.646	0.000333
32	204968_at	C6orf47	1.66	0.000413
33	206727_at	C9	1.969	0.00000621
34	212712_at	CAMSAP1	1.626	4.17E-11
35	221167_s_at	CCDC70	2.593	0.0000741
36	206407_s_at	CCL13	3.389	1.48E-15
37	219025_at	CD248	1.522	0.0000125
38	205692_s_at	CD38	1.657	4.46E-08
39	206680_at	CD5L	1.92	0.0000228
40	207729_at	CDH9	1.703	0.000129
41	207647_at	CDY1 (incl	1.615	0.0000716
42	204739_at	CENPC	1.512	0.000297
43	209667_at	CES2	1.757	0.00000611
44	220308_at	CFAP45	1.646	0.000443
45	200021_at	CFL1	1.588	0.00000169
46	217654_at	CFLAR	1.942	0.000389
47	207024_at	CHRND	1.538	0.000315
48	203921_at	CHST2	1.739	0.0000935
49	221065_s_at	CHST8	1.573	0.0000878
50	205101_at	CIITA	3.727	5.88E-10
51	219947_at	CLEC4A	2.309	0.00000044
52	205944_s_at	CLTCL1	1.762	2.76E-11
53	210571_s_at	CMAHP	2.457	0.000059
54	217404_s_at	COL2A1	1.706	0.00000539
55	211473_s_at	COL4A6	2.442	0.000000065
56	213428_s_at	COL6A1	1.616	0.000000505
57	208684_at	COPA	1.889	0.00000119
58	221550_at	COX15	1.526	0.00000472
59	205615_at	CPA1	1.583	1.28E-10
60	202977_s_at	CREBZF	3.472	7.4E-11

Up-regulated genes in residual colorectal tumors after CRT versus pre-treatment tumors (GSE93375, total 116 genes, P<0.005, FC≥2) © 2000- © 2000-2017 QIAGEN. All rights reserved.				
#	ID	Symbol	Expr Fold Change	Expr p-value
1	11748016_a_at	ACTG2	2.49254077	2.52E-03
2	11715793_a_at	ACTG2	2.14677077	1.31E-03
3	11715794_x_at	ACTG2	2.05645667	8.63E-04
4	11750687_a_at	ADAM28	1.19091342	2.53E-03
5	11722994_s_at	ADH1A ///	1.26906171	2.60E-03
6	11747167_x_at	ADH1B	1.20476214	4.58E-04
7	11730457_a_at	AIM2	1.34160333	2.59E-05
8	11720013_a_at	ATM	1.08761231	1.67E-03
9	11746528_a_at	ATP2B4	1.49360043	4.35E-05
10	11733023_s_at	BTG1	1.08851248	9.09E-05
11	11757933_s_at	BTG2	1.0392412	3.15E-03
12	11715614_at	BTG2	1.01674103	1.63E-03
13	11717978_at	C7	1.42891282	2.35E-04
14	11717980_x_at	C7	1.32794803	3.05E-03
15	11749537_a_at	C7	1.24053385	3.13E-03
16	11746954_s_at	CCL4 /// CC	1.29758462	2.48E-03
17	11718982_s_at	CCL4 /// CC	1.05182316	3.90E-03
18	11732275_at	CCL5	1.66635889	9.36E-05
19	11732276_x_at	CCL5	1.53792556	5.66E-05
20	11753810_a_at	CCL5	1.02712316	9.52E-04
21	11728679_a_at	CD163	1.53872197	1.42E-06
22	11716842_a_at	CD53	1.29937598	2.41E-04
23	11753555_a_at	CD53	1.12365077	3.05E-03
24	11764029_at	CEBPD	1.14346692	8.99E-05
25	11752869_s_at	CEBPD	1.11158017	1.07E-04
26	11764030_x_at	CEBPD	1.07524829	1.29E-04
27	11747163_a_at	CEP85L	1.18891282	6.13E-05
28	11756547_a_at	CLU	1.37485308	1.38E-03
29	11747737_x_at	CNN1	1.28600256	6.10E-04
30	11747736_s_at	CNN1	1.16851051	1.52E-03
31	11734310_a_at	CNN1	1.1108588	1.81E-03
32	11757921_s_at	COL14A1	1.6198306	3.10E-03
33	11749658_a_at	COL14A1	1.17953932	2.26E-03
34	11762326_at	CRISPLD2	1.25146308	5.48E-07
35	11753257_a_at	CXCL12	1.28807632	1.47E-04
36	11720818_a_at	CXCL12	1.20073538	2.24E-04
37	11728189_a_at	CXCR4	2.36070521	2.52E-05
38	11728191_x_at	CXCR4	1.9884006	8.15E-06
39	11739094_a_at	CXCR4	1.92277316	1.43E-05
40	11728190_s_at	CXCR4	1.78604983	1.50E-04
41	11749905_a_at	CYR61	1.64176333	4.30E-04
42	11734690_a_at	CYTIP	1.16248162	2.84E-03
43	11717048_a_at	DES	1.8944353	2.14E-04
44	11717049_s_at	DES /// SUI	1.89515726	4.81E-05
45	11715766_a_at	DUSP1	2.11246043	3.40E-04
46	11752993_a_at	DUSP1	1.52321427	2.91E-03
47	11747508_a_at	EPB41L3	1.31533932	5.81E-06
48	11727142_a_at	EPB41L3	1.05406017	6.76E-06
49	11727143_x_at	EPB41L3	1.04016376	6.48E-05
50	11755895_a_at	FAM129A	1.07709718	4.84E-04
51	11739340_at	FAM46C	1.07758915	2.43E-03
52	11719394_a_at	FBXO32	1.35684085	7.64E-04
53	11734947_a_at	FGF7	1.10963026	3.73E-03
54	11733818_x_at	FHL1	2.05290342	5.49E-04
55	11745722_x_at	FHL1	2.01684761	6.96E-04
56	11733817_s_at	FHL1	1.94588197	8.79E-05
57	11753010_x_at	FHL1	1.57893573	9.96E-05
58	11748655_x_at	FHL1	1.56850085	6.46E-04
59	11753599_x_at	FHL1	1.50902991	2.22E-05
60	11753338_x_at	FHL1	1.45812137	4.58E-05

61	205474_at	CRLF3	1.559	0.00000192
62	200838_at	CTSB	1.53	0.00000176
63	211122_s_at	CXCL11	1.743	0.000499
64	215101_s_at	CXCL5	2.351	0.000133
65	203922_s_at	CYBB	2.031	2.01E-08
66	200046_at	DAD1	1.562	0.0000467
67	1007_s_at	DDR1	1.574	0.0000281
68	31807_at	DDX49	1.629	0.000303
69	207959_s_at	DNAH9	1.802	0.000000216
70	209015_s_at	DNAJB6	3.962	0.000000707
71	207192_at	DNASE1L	2.564	0.00000001
72	212538_at	DOCK9	1.713	0.000000234
73	204464_s_at	EDNRA	1.822	0.00000665
74	220006_at	EFCC1	4.664	2.81E-11
75	205222_at	EHHADH	1.634	0.00000209
76	200023_s_at	EIF3F	1.744	0.000000432
77	220624_s_at	ELF5	3.226	0.00000679
78	214445_at	ELL2	1.823	0.000000127
79	206605_at	ENDOU	1.94	0.00000665
80	206191_at	ENTPD3	3.147	1.15E-12
81	220977_x_at	EPB41L5	1.873	0.000000532
82	212087_s_at	ERAL1	1.765	0.000000183
83	203719_at	ERCC1	2.169	0.000000667
84	210158_at	ERCC4	2.414	0.00000302
85	203249_at	EZH1	1.523	0.0000246
86	205756_s_at	F8	3.433	0.000000411
87	203980_at	FABP4	2.969	3.03E-08
88	202916_s_at	FAM20B	5.824	3.33E-18
89	201889_at	FAM3C	1.969	0.000000411
90	216897_s_at	FAM76A	2.11	0.000104
91	211333_s_at	FASLG	1.778	3.27E-09
92	210889_s_at	FCGR2B	1.501	0.00000496
93	206412_at	FER	1.933	6.15E-09
94	205973_at	FEZ1	1.568	0.0000042
95	215000_s_at	FEZ2	4.935	0.0000117
96	210655_s_at	FOXO3B	2.958	0.0000348
97	209990_s_at	GABBR2	1.525	0.000000682
98	206670_s_at	GAD1	2.327	1.07E-10
99	209729_at	GAS2L1	1.526	0.00000142
100	210358_x_at	GATA2	1.535	0.0000545
101	202832_at	GCC2	1.982	0.00028
102	205505_at	GCNT1	3.676	6.72E-12
103	200009_at	GDI2	11.592	0.0000491
104	205527_s_at	GEMIN4	1.639	0.00000132
105	208913_at	GGA2	1.969	2.49E-08
106	206195_x_at	GH2	2.667	3.9E-13
107	207899_at	GIP	1.623	0.0000158
108	204763_s_at	GNAO1	2.737	5.89E-10
109	214605_x_at	GPR1	1.862	0.0000621
110	206190_at	GPR17	6.973	7.01E-10
111	214864_s_at	GRHPR	1.569	1.25E-09
112	207454_at	GRIK3	1.709	1.17E-08
113	207036_x_at	GRIN2D	6.202	5.13E-15
114	208465_at	GRM2	1.711	0.0000529
115	210234_at	GRM4	1.746	0.0000577
116	221549_at	GRWD1	1.539	0.00000663
117	210892_s_at	GTF2I	1.745	0.0000248
118	220142_at	HAPLN2	1.563	1.47E-10
119	207642_at	HCRT	1.632	0.00000706
120	209558_s_at	HIP1R	2.214	1.38E-10

61	11753598_a_at	FHL1	1.3968735	7.98E-05
62	11747328_a_at	FHL1	1.35841419	3.74E-03
63	11747329_x_at	FHL1	1.23825316	9.28E-04
64	11743917_a_at	FKBP5	1.90982051	3.56E-07
65	11739566_a_at	FKBP5	1.56715043	5.51E-08
66	11739567_s_at	FKBP5	1.56700675	1.21E-05
67	11746275_a_at	FKBP5	1.32384667	1.49E-06
68	11739565_a_at	FKBP5	1.29194265	5.80E-06
69	11727111_a_at	FNBP1	1.05590436	1.22E-03
70	11719447_s_at	GBP2	1.22408795	1.33E-03
71	11720558_a_at	GEM	1.12160607	8.99E-05
72	11721625_s_at	GLUL	1.51120615	1.18E-04
73	11725521_x_at	GLUL	1.42191889	6.85E-05
74	11744337_a_at	GLUL	1.33984906	2.21E-04
75	11758555_s_at	GPR183	1.12888855	2.56E-04
76	11720496_at	GZMA	1.15665692	7.90E-04
77	11715514_a_at	HERPUD1	1.17457658	1.05E-03
78	11749257_a_at	HERPUD1	1.04771376	4.30E-03
79	11741510_a_at	HERPUD1	1.04609966	1.12E-03
80	11738103_at	HIST1H4F	1.08531829	4.71E-03
81	11716554_a_at	HLA-DMA	1.07762863	1.94E-03
82	11746961_a_at	HLA-DMB	1.41276821	3.02E-03
83	11758231_x_at	HLA-DPA1	1.88436573	1.44E-03
84	11758417_s_at	HLA-DPA1	1.29664615	3.64E-03
85	11715583_s_at	HLA-DPA1	1.23343829	4.20E-03
86	11757511_x_at	HLA-DPA1	1.06871829	3.16E-03
87	11758369_x_at	HLA-DPB1	2.91386444	1.26E-03
88	11757801_x_at	HLA-DPB1	2.37598179	4.14E-05
89	11756073_x_at	HLA-DPB1	1.8587406	2.75E-03
90	11759666_x_at	HLA-DPB1	1.13528504	2.70E-03
91	11758772_x_at	HLA-DPB1	1.00083051	2.16E-03
92	11753898_x_at	HLA-DQA1	1.66624521	2.70E-04
93	11758340_x_at	HLA-DQA1	1.18993316	1.29E-03
94	11723194_x_at	HLA-DRB1	1.33985256	2.56E-03
95	11740359_a_at	HOXD10 ///	1.41870709	6.13E-04
96	11746088_a_at	IFI44	1.11385803	2.45E-03
97	11745244_x_at	IGHG1 /// I	1.75767239	2.67E-03
98	11754032_x_at	IGHG1 /// I	1.70608974	4.07E-03
99	11760929_x_at	IGHG1 /// I	2.24258632	1.91E-03
100	11759852_x_at	IGHG1 /// I	1.84556137	2.78E-03
101	11750231_x_at	IGHG1 /// I	1.81942453	2.40E-03
102	11760819_x_at	IGHG1 /// I	2.0115006	2.34E-03
103	11761467_x_at	IGHG3	1.94497137	1.34E-03
104	11753878_s_at	IL6ST	1.17053051	1.66E-03
105	11753886_a_at	IL6ST	1.12498026	2.22E-04
106	11753579_a_at	IL6ST	1.01715915	2.83E-03
107	11753667_s_at	ITM2C	1.15798342	1.20E-03
108	11755661_a_at	KCNMA1	1.63718949	2.16E-04
109	11744702_a_at	KCNMA1	1.15441684	3.92E-06
110	11759671_s_at	KCNMA1	1.13692974	2.33E-06
111	11753220_a_at	KCNMB1	1.34971615	4.19E-03
112	11717327_at	KLF9	1.03434752	9.99E-04
113	11727695_a_at	KLRC4-KLR	1.16875607	2.84E-03
114	11716771_s_at	LOC102724	1.16659573	4.68E-04
115	11757798_s_at	MAFB	1.09403829	2.41E-04
116	11745724_at	MALAT1	1.1713253	1.84E-03
117	11741548_a_at	MBNL1	1.20734077	5.51E-05
118	11715484_a_at	MCL1	1.19341812	3.80E-03
119	11716846_a_at	MS4A6A	1.38035675	1.45E-03
120	11728397_at	MT1M	1.12490479	2.33E-05

121	214290_s_at	HIST2H2A.	1.898	0.000111		121	11757581_x_at	MT1X	1.42126521	3.33E-06
122	214604_at	HOXD11	7.303	2.63E-11		122	11726385_a_at	MT1X	1.2310441	8.89E-06
123	205580_s_at	HRH1	19.645	9.84E-15		123	11753900_x_at	MT2A	2.0471788	5.66E-04
124	206294_at	HSD3B2	4.462	4.52E-09		124	11732179_x_at	MYH11	2.02698393	1.16E-03
125	117_at	HSPA6	1.606	0.00000084		125	11732178_a_at	MYH11	1.89372325	1.14E-03
126	206855_s_at	HYAL2	1.584	0.00000423		126	11732177_s_at	MYH11	1.60839085	1.19E-03
127	209575_at	IL10RB	1.557	0.00000581		127	11727361_a_at	MYLK	2.18303846	3.45E-03
128	206890_at	IL12RB1	2.455	4.81E-11		128	11725110_a_at	NDE1	1.39450863	1.66E-03
129	206295_at	IL18	3.65	1.44E-11		129	11756077_a_at	NDE1	1.20746145	1.75E-03
130	220322_at	IL36G	2.761	0.000123		130	11717994_a_at	NR4A1	1.23065009	2.33E-04
131	205798_at	IL7R	1.929	0.0000119		131	11717995_x_at	NR4A1	1.00709846	9.00E-05
132	205376_at	INPP4B	1.749	0.0000878		132	11743830_a_at	PAM	1.01407752	5.51E-04
133	206766_at	ITGA10	1.728	3.95E-08		133	11743354_a_at	PDCD4	1.19914368	1.69E-04
134	204990_s_at	ITGB4	2.871	0.000146		134	11716975_a_at	PDK4	1.23896496	2.13E-05
135	205842_s_at	JAK2	2.754	0.000000148		135	11717168_a_at	PER1	1.42894769	1.44E-06
136	203845_at	KAT2B	1.67	0.000105		136	11756898_a_at	PGM5	1.30434564	3.88E-03
137	215138_s_at	KAZN	1.523	0.0000312		137	11739541_a_at	PIK3R1	1.01778154	2.87E-03
138	205903_s_at	KCNN3	2.262	0.00000925		138	11754033_a_at	PLA2G2A	2.06029949	4.23E-04
139	211486_s_at	KCNQ2	1.722	0.0000206		139	11731550_a_at	PLSCR4	1.05140752	2.17E-04
140	206017_at	KIAA0319	3.378	7.59E-10		140	11749039_x_at	PNRC1	1.42600701	1.09E-03
141	206551_x_at	KLHL24	1.581	3.45E-10		141	11752095_a_at	PTPRC	1.52148709	1.54E-03
142	220646_s_at	KLRF1	1.677	0.0000155		142	11748907_a_at	RARRES3	1.09580795	3.87E-03
143	200650_s_at	LDHA	1.615	9.28E-08		143	11743171_a_at	RCSD1	1.02309077	6.45E-05
144	217173_s_at	LDLR	1.575	0.0000135		144	11742765_at	RGS1	1.74008017	2.00E-04
145	207409_at	LECT2	1.509	0.000259		145	11715757_a_at	RGS2	1.68204932	2.36E-04
146	210731_s_at	LGALS8	1.537	0.000000212		146	11757177_s_at	RNF149 ///	1.56097385	1.36E-03
147	215929_at	LINC00837	2.076	5.69E-08		147	11763955_at	SCARNA10	1.3486253	1.15E-03
148	208186_s_at	LIPE	1.576	0.000262		148	11757260_at	SCARNA10	1.29938282	3.67E-03
149	219181_at	LIPG	1.518	0.0000336		149	11731433_a_at	SEPP1	1.70707504	2.02E-03
150	220764_at	LOC10537	1.519	0.000127		150	11741874_x_at	SEPP1	1.6216541	3.68E-04
151	207762_at	LPAL2	1.571	0.000175		151	11720606_a_at	SFRP2	1.98847094	2.00E-03
152	209840_s_at	LRRN3	1.545	0.000108		152	11745903_a_at	SLAMF7	1.1774388	2.01E-03
153	214460_at	LSAMP	2.02	0.000205		153	11742188_a_at	SLC4A4	1.21436581	4.28E-03
154	203534_at	LSM1	3.983	2.25E-09		154	11747948_a_at	SMAP2	1.53630573	6.75E-06
155	206609_at	MAGEC1	2.012	0.0000456		155	11719845_a_at	SMAP2	1.21037222	2.57E-07
156	209014_at	MAGED1	12.511	3.3E-17		156	11757163_at	SNORA54	1.29341769	2.66E-03
157	206296_x_at	MAP4K1	2.549	4.23E-15		157	11754272_x_at	SNRPN /// !	1.05152889	3.14E-04
158	206040_s_at	MAPK11	1.671	5.47E-09		158	11732913_a_at	SP140	1.3111065	3.05E-04
159	221047_s_at	MARK1	1.683	0.0000285		159	11752251_a_at	SPARCL1	1.87432684	2.88E-04
160	210958_s_at	MAST4	1.907	0.000123		160	11725023_a_at	SPARCL1	1.79961726	1.30E-04
161	216567_at	MBP	1.611	5.24E-08		161	11730298_a_at	SPARCL1	1.65258726	5.26E-04
162	205386_s_at	MDM2	3.215	1.45E-15		162	11742710_a_at	SRGN	1.90330077	5.20E-05
163	214778_at	MEGF8	1.875	0.000000694		163	11760894_s_at	SRSF5	1.09644838	4.13E-03
164	214972_at	MGEA5	1.505	0.000285		164	11718364_a_at	ST6GALNA	1.46651538	2.07E-03
165	221177_at	MIA2	1.604	0.000122		165	11726689_a_at	STAT1	1.07236897	3.27E-03
166	221365_at	MLNR	2.389	0.000257		166	11728497_s_at	SVIL	1.0341706	4.54E-03
167	220688_s_at	MRTO4	2.04	7.95E-08		167	11755757_a_at	SYNM	1.9106941	3.43E-03
168	207496_at	MS4A2	2.302	0.00000468		168	11740786_a_at	SYNM	1.17753855	4.83E-04
169	210533_at	MSH4	1.945	1.36E-08		169	11731557_at	SYNPO2	1.40553675	3.08E-03
170	204956_at	MTAP	4.372	2.47E-10		170	11721091_a_at	THBS1	1.16253308	4.04E-03
171	216095_x_at	MTMR1	1.822	0.000468		171	11763675_at	THEMIS2	1.14058077	2.61E-04
172	216671_x_at	MUC8	1.687	0.000125		172	11752610_a_at	THEMIS2	1.09772573	2.03E-04
173	206717_at	MYH8	1.967	1.83E-10		173	11721615_a_at	THEMIS2	1.06982214	1.40E-04
174	219728_at	MYOT	1.531	0.0000224		174	11718611_at	TP53INP1	1.43723214	2.49E-04
175	220656_at	NAA16	1.88	0.000294		175	11722369_x_at	TRIM22	1.15435786	3.09E-04
176	207279_s_at	NEBL	1.516	3.74E-11		176	11750170_a_at	TRIM22	1.02612624	3.63E-04
177	215005_at	NECAB2	2.151	1.77E-09		177	11717830_a_at	TSC22D3	2.15434735	1.44E-05
178	210162_s_at	NFATC1	3.308	5.37E-08		178	11717829_s_at	TSC22D3	1.92113641	9.49E-05
179	210268_at	NFX1	1.562	0.000154		179	11751415_a_at	TSC22D3	1.75332462	1.92E-06
180	219594_at	NINJ2	1.794	0.00000136		180	11719030_a_at	TSPYL2	1.33232171	5.34E-04

181	207075_at	NLRP3	1.821	0.0000317		181	11752765_s_at	TXNIP	1.76426085	5.38E-05
182	205581_s_at	NOS3	10.186	1.3E-15		182	11748543_a_at	TXNIP	1.7370294	2.41E-05
183	205460_at	NPAS2	1.545	0.00000431		183	11717190_s_at	TXNIP	1.72707991	1.41E-05
184	216344_at	NPHP4	1.677	0.0000442		184	11748544_s_at	TXNIP	1.4782812	3.21E-06
185	205259_at	NR3C2	1.749	0.0000442		185	11756431_s_at	TXNIP	1.46024573	5.76E-05
186	204621_s_at	NR4A2	1.963	0.000217		186	11746454_a_at	USP15	1.01510179	6.43E-04
187	214632_at	NRP2	1.931	0.00000209		187	11746616_a_at	WSB1	1.10798479	6.77E-04
188	201173_x_at	NUDC	1.611	0.000000502		188	11729371_a_at	ZBTB16	1.01816692	2.02E-04
189	206215_at	OPCML	1.988	2.36E-09		189	11715691_s_at	ZFP36	1.15287573	3.21E-03
190	221327_s_at	OPN1MW	1.94	0.0000654						
191	206880_at	P2RX6	1.713	0.0000174						
192	220005_at	P2RY13	3.391	5.52E-10						
193	210160_at	PAFAH1B	2.373	0.00000206						
194	208051_s_at	PAIP1	1.694	3.39E-10						
195	218886_at	PAK1IP1	1.541	0.000112						
196	205962_at	PAK2	2.111	0.0000115						
197	206594_at	PASK	4.429	2.47E-10						
198	205253_at	PBX1	1.67	7.67E-09						
199	208366_at	PCDH11X	1.517	0.000217						
200	211877_s_at	PCDHGA1	2.242	0.000292						
201	214826_at	PDE12	2.176	0.00000236						
202	210937_s_at	PDX1	2.017	0.000128						
203	200886_s_at	PGAM1	2.811	1.24E-17						
204	204049_s_at	PHACTR2	1.54	0.00000174						
205	207081_s_at	PI4KA	1.765	0.0000175						
206	204691_x_at	PLA2G6	1.569	0.00000561						
207	203470_s_at	PLEK	1.608	0.00000128						
208	204519_s_at	PLLP	1.531	1.04E-08						
209	212235_at	PLXND1	1.705	0.000161						
210	213893_x_at	PMS2P5/F	1.522	0.00000489						
211	210830_s_at	PON2	1.55	0.000175						
212	203338_at	PPP2R5E	1.656	6.12E-09						
213	209766_at	PRDX3	1.537	0.0000116						
214	216051_x_at	PRINS	1.695	0.000333						
215	207957_s_at	PRKCB	2.035	0.000000016						
216	209334_s_at	PSMD9	1.942	0.000159						
217	209852_x_at	PSME3	1.867	0.0000111						
218	206361_at	PTGDR2	5.384	2.81E-11						
219	207238_s_at	PTPRC	10.284	3.43E-21						
220	205924_at	RAB3B	1.772	2.76E-11						
221	208640_at	RAC1	1.581	1.43E-08						
222	205326_at	RAMP3	1.523	0.00000676						
223	213852_at	RBM8A	1.556	2.49E-08						
224	205091_x_at	RECQL	2.478	0.00000136						
225	1053_at	RFC2	1.741	0.000032						
226	203169_at	RGP1	1.783	0.0000148						
227	211872_s_at	RGS11	11.528	2.82E-12						
228	202976_s_at	RHOBTB3	3.369	5.04E-13						
229	206154_at	RLBP1	2.866	0.0000545						
230	220329_s_at	RMND1	1.744	0.00000103						
231	206845_s_at	RNF40	2.47	8.58E-09						
232	207939_x_at	RNPS1	1.507	0.000187						
233	206608_s_at	RPGRIP1	1.814	0.0000333						
234	213959_s_at	RPGRIP1L	2	0.0000159						
235	200010_at	RPL11	3.48	0.000163						
236	200022_at	RPL18	1.7	0.0000184						
237	200029_at	RPL19	1.529	1.59E-08						
238	207283_at	RPL23AP3	1.558	0.0000649						
239	200026_at	RPL34	1.563	7.73E-10						
240	200018_at	RPS13	2.898	0.00000262						

241	200017_at	RPS27A	4.005	0.00000214
242	200024_at	RPS5	1.51	0.000000322
243	200858_s_at	RPS8	1.869	2.52E-08
244	218166_s_at	RSF1	2.962	0.000000476
245	200042_at	RTCB	3.203	0.00043
246	211509_s_at	RTN4	5.452	1.73E-08
247	205528_s_at	RUNX1T1	14.916	8.85E-14
248	216162_at	SBNO1	1.596	0.000266
249	206799_at	SCGB1D2	4.654	2.57E-11
250	207295_at	SCNN1G	2.061	0.000000183
251	206832_s_at	SEMA3F	1.536	0.000194
252	205405_at	SEMA5A	1.723	0.0000916
253	214293_at	SEPT11	1.635	0.000184
254	208313_s_at	SF1	2.489	0.0000111
255	214781_at	SHANK1	1.507	0.00000398
256	214095_at	SHMT2	2.51	7.95E-12
257	217278_x_at	SHOX2	1.939	0.00000746
258	206510_at	SIX2	3.943	2.13E-11
259	210423_s_at	SLC11A1	2.605	7.82E-08
260	205244_s_at	SLC13A3	2.263	0.000262
261	205074_at	SLC22A5	5.112	1.13E-12
262	201802_at	SLC29A1	1.974	3.02E-08
263	220413_at	SLC39A2	3.303	2.23E-14
264	217859_s_at	SLC39A9	1.924	0.000000856
265	205920_at	SLC6A6	1.539	0.000017
266	215469_at	SLITRK5	1.741	7.35E-08
267	206565_x_at	SMA4	1.546	3.93E-08
268	205622_at	SMPD2	1.904	0.000457
269	206360_s_at	SOCS3	2.154	0.00000274
270	210536_s_at	SPAM1	2.814	0.0000835
271	215383_x_at	SPG21	2.672	0.000000424
272	209857_s_at	SPHK2	1.677	0.0000271
273	200044_at	SRSF9	13.747	0.000187
274	215710_at	ST3GAL4	1.719	0.0000434
275	207524_at	ST7	1.608	0.000000707
276	211078_s_at	STK3	1.678	4.48E-09
277	200783_s_at	STMN1	1.556	3.74E-11
278	210247_at	SYN2	1.637	5.36E-08
279	206161_s_at	SYT5	1.868	0.0000196
280	221393_at	TAAR3P	1.516	0.0000605
281	204877_s_at	TAOK2	1.534	0.000000156
282	204931_at	TCF21	1.589	9.96E-08
283	210776_x_at	TCF3	2.248	0.000102
284	205254_x_at	TCF7	1.883	0.00000201
285	41037_at	TEAD4	1.597	0.00000121
286	214476_at	TFF2	1.577	8.42E-08
287	207334_s_at	TGFBR2	1.512	0.000000688
288	206260_at	TGM4	2.96	0.00000162
289	208700_s_at	TKT	1.907	0.00000063
290	216100_s_at	TOR1AIP1	1.974	0.0000237
291	206117_at	TPM1	2.45	0.0000111
292	203375_s_at	TPP2	3.139	2.21E-08
293	210733_at	TRAM1	1.604	0.0000226
294	210159_s_at	TRIM31	4.325	9.88E-08
295	200668_s_at	UBE2D3	1.897	2.08E-10
296	217825_s_at	UBE2J1	1.569	0.000032
297	220083_x_at	UCHL5	1.797	0.000043
298	215737_x_at	USF2	1.541	0.0000408
299	203940_s_at	VASH1	2.206	2.45E-11
300	207045_at	VPS50	8.059	0.000000151
301	211992_at	WNK1	3.229	2.47E-10
302	205648_at	WNT2	2.101	6.86E-14
303	210561_s_at	WSB1	1.815	0.000119
304	217065_at	YME1L1	1.504	0.00000134
305	212455_at	YTHDC1	1.565	0.000395
306	200047_s_at	YY1	3.462	0.000173
307	214482_at	ZBTB25	1.517	0.00000417
308	206744_s_at	ZMYM5	2.136	0.0000305
309	207296_at	ZNF343	1.733	0.000162
310	216780_at	ZNF443	3.139	3.9E-09
311	205494_at	ZNF821	2.68	0.0000208

Down-regulated genes in metastatic colorectal cancer versus primary tumor (GSE68468, total 222 genes, P<0.0005, FC≤1.5)

© 2000-2017 QIAGEN. All rights reserved.

#	ID	Symbol	Expr Fold Change	Expr p-value
1	202502_at	ACADM	-1.886	0.000114
2	205213_at	ACAP1	-3.236	0.0000106
3	206811_at	ADCY8	-1.533	0.000397
4	209614_at	ADH1B	-1.813	0.00000293
5	203321_s_	ADNP2	-1.687	0.000000227
6	222160_at	AKAP8L	-1.997	0.000201
7	220365_at	ALLC	-3.46	0.0000187
8	206656_s_	APMAP	-1.631	0.00000172
9	214483_s_	ARFIP1	-1.615	0.000113
10	201878_at	ARIH1	-1.981	0.00000574
11	217852_s_	ARL8B	-1.98	0.0000064
12	213768_s_	ASCL1	-1.561	0.000000196
13	204903_x_	ATG4B	-1.648	0.0000207
14	204516_at	ATXN7	-2.477	0.0000659
15	219688_at	BBS7	-1.885	1.04E-08
16	203755_at	BUB1B	-1.583	0.000000444
17	219009_at	C14orf93	-3.694	1.19E-12
18	218130_at	C17orf62	-2.558	4.36E-10
19	219010_at	C1orf106	-2.809	8.53E-11
20	219288_at	C3orf14	-1.717	0.0000187
21	204508_s_	CA12	-1.511	0.000000557
22	214880_x_	CALD1	-1.61	0.00000907
23	212252_at	CAMKK2	-1.877	0.000198
24	211208_s_	CASK	-1.979	0.000011
25	202763_at	CASP3	-2.549	5.88E-10
26	220018_at	CBLL1	-1.533	0.000042
27	204609_at	CCDC85B	-1.522	0.0000521
28	209953_s_	CDC37	-1.955	0.0000117
29	214464_at	CDC42BP/	-1.674	0.000136
30	207172_s_	CDH11	-1.763	0.0000143
31	222063_s_	CDS1	-2.305	0.000000499
32	218542_at	CEP55	-4.357	2.86E-08
33	203536_s_	CIAO1	-6.944	0.000000114
34	210716_s_	CLIP1	-1.523	0.0000567
35	220739_s_	CNNM3	-1.526	0.000012
36	210867_at	CNOT4	-3.012	1.37E-20
37	220095_at	CNTLN	-1.77	0.000164
38	203073_at	COG2	-2.591	0.0000325
39	212937_s_	COL6A1	-3.201	0.0000153
40	218358_at	CRELD2	-3.953	0.0000348
41	214334_x_	DAZAP2	-1.579	0.000245
42	201571_s_	DCTD	-1.824	0.000259
43	212384_at	DDX39B	-1.726	0.0000099
44	209190_s_	DIAPH1	-1.529	0.000163
45	213546_at	DKFZP586	-1.922	3.02E-08
46	215266_at	DNAH3	-2.064	7.19E-11
47	215252_at	DNAJC7	-1.645	0.000000241
48	206531_at	DPF1	-3.181	1.17E-16
49	205031_at	EFNB3	-1.612	6.99E-08
50	205249_at	EGR2	-1.503	0.000306
51	212225_at	EIF1	-1.57	0.000143
52	220029_at	ELOVL2	-1.504	0.000143
53	210868_s_	ELOVL6	-3.09	1.13E-08
54	202017_at	EPHX1	-1.521	4.92E-08
55	206674_at	FLT3	-2.814	1.78E-09
56	207178_s_	FRK	-2.165	5.98E-08
57	215052_at	FRMPD4	-3.072	3.46E-08
58	209702_at	FTO	-1.736	0.000093
59	203725_at	GADD45A	-1.639	8.35E-12
60	210565_at	GCGR	-1.69	0.000483

Down-regulated genes in residual colorectal tumors after CRT versus pre-treatment tumors (GSE93375, total 64 genes, P<0.005, FC≤2)

© 2000-2017 QIAGEN. All rights reserved.

#	ID	Symbol	Expr Fold Change	Expr p-value
1	11737746_a_a	ADGRG1	-1.4489671	7.45E-04
2	11732450_s_a	AGRN	-1.214762	1.48E-03
3	11751629_a_a	ASF1B	-1.0696494	8.92E-04
4	11732853_at	ATP1B4	-1.2429976	2.27E-03
5	11754109_s_a	BIRC5	-1.6215812	3.81E-03
6	11747653_x_a	CDC25B	-1.0178781	3.00E-03
7	11747652_a_a	CDC25B	-1.0207493	4.93E-03
8	11758478_s_a	CDCA7	-1.2553986	3.86E-05
9	11756069_x_a	CDKN3	-1.202233	4.45E-03
10	11725788_a_a	CENPN	-1.0804103	7.68E-04
11	11728233_a_a	CLDN1	-1.3813933	2.50E-03
12	11724872_a_a	CLDN2	-1.8488924	4.48E-03
13	11715290_s_a	CLDN3	-1.0663775	3.90E-03
14	11762083_at	CSNK2A1	-1.0059327	1.69E-03
15	11754114_a_a	CXCL1	-1.0020461	9.58E-04
16	11733296_s_a	CYP4F2 /// C	-1.096088	3.18E-03
17	11729446_a_a	DCT	-1.0069681	3.15E-03
18	11763218_at	DEFB121	-1.0514658	4.89E-03
19	11758393_s_a	DNAJC30	-1.1027143	2.00E-03
20	11723166_a_a	EPHB2	-1.3215353	1.58E-03
21	11747996_a_a	ETV4	-1.1656323	1.18E-03
22	11762530_x_a	FERMT1	-1.4045838	9.95E-05
23	11755613_a_a	FERMT1	-2.3591342	1.19E-04
24	11758028_s_a	FOXQ1	-1.0496865	3.48E-03
25	11749970_a_a	GINS1	-1.035843	2.02E-04
26	11755276_a_a	GPX2	-1.7622165	4.95E-03
27	11754183_s_a	HMGB3	-1.1179417	3.13E-03
28	11730058_at	HNF4A	-1.5551777	3.31E-03
29	11737053_s_a	HSPD1	-1.071725	3.31E-03
30	11753017_s_a	HSPD1 /// HS	-1.4465407	9.12E-04
31	11717096_a_a	HSPH1	-1.2336645	1.54E-03
32	11753661_a_a	ID1	-1.4819695	1.17E-03
33	11756753_a_a	IQGAP3	-1.3465217	5.28E-04
34	11723896_a_a	JAG2	-1.500645	9.05E-06
35	11754765_s_a	KPNA2 /// LC	-1.178724	1.49E-04
36	11752331_s_a	LOC1053692	-1.0071931	5.10E-04
37	11751835_a_a	LTV1	-1.1476567	3.94E-03
38	11743722_x_a	MARCKSL1	-1.0350974	1.79E-05
39	11742996_a_a	MCM3	-1.0068156	5.18E-05
40	11721142_a_a	MKI67	-1.1934876	2.57E-04
41	11721143_a_a	MKI67	-1.3629497	6.51E-05
42	11746135_x_a	NOP56	-1.0688361	4.24E-04
43	11755342_x_a	NOP56	-1.1078862	8.20E-05
44	11758679_s_a	NOP56	-1.1889887	3.52E-04
45	11746134_s_a	NOP56	-1.2223153	3.48E-04
46	11718796_x_a	PAQR4	-1.152361	3.83E-05
47	11756635_a_a	PLCB4	-1.080865	4.85E-04
48	11735722_a_a	PNKD	-1.1099886	4.45E-04
49	11743950_s_a	POU4F1	-1.0839214	3.57E-03
50	11758134_s_a	PPM1H	-1.0455768	1.19E-04
51	11759665_a_a	SLC1A6	-1.1189464	1.64E-03
52	11742878_a_a	SLC52A2	-1.1118376	4.70E-03
53	11720333_a_a	SLC5A6	-1.1279472	8.28E-04
54	11757660_a_a	SNRPB	-1.2254542	1.71E-03
55	11719561_s_a	SOX4	-1.1617515	1.78E-03
56	11720447_s_a	SOX9	-1.2658216	9.11E-04
57	11751072_a_a	SPATA2	-1.6113186	3.71E-04
58	11717624_at	TBL2	-1.0463377	1.43E-03
59	11733681_a_a	TMEM132D	-1.0171428	4.60E-03
60	11721633_s_a	TMEM97	-1.0734433	1.77E-03

61	214106_s_	GMDS	-1.541	0.000407			61	11721632_a_a	TMEM97	-1.5441796	2.47E-04
62	204220_at	GMFG	-2.216	2.43E-10			62	11720970_at	TOP2A	-1.6254268	6.33E-05
63	204248_at	GNA11	-1.952	0.0000935			63	11750598_s_a	TPX2	-1.1105067	5.57E-04
64	205010_at	GNL3L	-2.031	0.000000234			64	11756670_x_a	TUBA1C	-1.0727344	3.86E-03
65	204984_at	GPC4	-1.557	0.00000113			65	11717939_a_a	U2AF2	-1.1703863	2.64E-03
66	205419_at	GPR183	-5.877	1.92E-09			66	11733695_a_a	UBE2C	-1.1441817	4.15E-04
67	214586_at	GPR37	-2.726	0.0000678			67	11724328_a_a	UBE2C	-1.3292567	1.62E-04
68	206712_at	GRTP1	-1.636	0.0000241			68	11761188_x_a	VIL1	-1.0943304	3.81E-03
69	220190_s_	GTF2A1L	-1.646	0.0000282			69	11729734_a_a	VIL1	-1.1039811	3.27E-03
70	202487_s_	H2AFV	-1.74	2.86E-08			70	11752343_a_a	XKRX	-1.0264156	4.72E-04
71	202815_s_	HEXIM1	-3.628	2.34E-15			71	11755870_s_a	YTHDF1	-1.0399127	6.59E-04
72	207721_x_	HINT1	-2.293	0.00000063			72	11738998_a_a	ZDHHC9	-1.0403087	4.75E-03
73	211931_s_	HNRNPA3	-1.822	0.00000907			73	11756626_s_a	ZWINT	-1.5228266	6.16E-05
74	205601_s_	HOXB5	-1.653	0.000000782							
75	206745_at	HOXC11	-2.335	0.000404							
76	205454_at	HPCA	-1.742	0.000372							
77	201610_at	ICMT	-2.792	6.46E-09							
78	210666_at	IDS	-1.816	0.0000467							
79	209541_at	IGF1	-2.557	0.0000345							
80	216573_at	IGLV1-44	-2.806	0.0000752							
81	220054_at	IL23A	-1.812	0.00000496							
82	211516_at	IL5RA	-1.543	0.000102							
83	203607_at	INPP5F	-1.557	0.00000151							
84	35776_at	ITSN1	-3.355	0.00000188							
85	213715_s_	KANK3	-2.017	0.0000471							
86	220412_x_	KCNK7	-1.805	0.000055							
87	212355_at	KHNYN	-1.655	0.0000659							
88	212303_x_	KHSRP	-1.666	0.0000478							
89	205306_x_	KMO	-1.667	0.000366							
90	34031_i_at	KRIT1	-4.243	2.13E-11							
91	213287_s_	KRT10	-1.533	0.000191							
92	210306_at	L3MBTL1	-1.843	0.000000286							
93	215516_at	LAMB4	-3.142	0.00000016							
94	212682_s_	LMF2	-1.527	0.000183							
95	211050_x_	LOC10028	-2.116	0.0000668							
96	216455_at	LOC10192	-3.26	0.0000525							
97	214110_s_	LOC65434	-1.931	0.000328							
98	210909_x_	LPAL2	-1.61	0.0000118							
99	202737_s_	LSM4	-1.626	0.000103							
100	202903_at	LSM5	-2.034	0.0000103							
101	202729_s_	LTBP1	-1.841	0.00000238							
102	214612_x_	MAGEA3/I	-1.512	0.000281							
103	202653_s_	MARCH7	-2.382	0.000000629							
104	201555_at	MCM3	-1.642	2.44E-09							
105	202610_s_	MED14	-1.505	0.000000368							
106	221192_x_	MFSD11	-1.977	0.00000254							
107	204580_at	MMP12	-1.872	0.00000496							
108	219909_at	MMP28	-1.563	0.00038							
109	219967_at	MRM1	-1.636	1.77E-08							
110	218890_x_	MRPL35	-1.755	8.79E-08							
111	219281_at	MSRA	-1.531	0.000031							
112	215793_at	MTMR7	-1.606	0.000131							
113	209596_at	MXRA5	-1.772	0.0000122							
114	202431_s_	MYC	-1.567	0.000000242							
115	208148_at	MYH4	-1.576	0.00000841							
116	202608_s_	NDST1	-1.845	0.000496							
117	204325_s_	NF1	-1.968	0.0000041							
118	217150_s_	NF2	-1.567	0.0000467							
119	204239_s_	NNAT	-2.887	0.0000401							
120	214685_at	NOP14-AS	-2.066	0.0000796							

121	206476_s_	NOVA2	-2.15	4.76E-13						
122	210444_at	NPY6R	-1.536	0.00000539						
123	207877_s_	NVL	-1.781	0.000000124						
124	214306_at	OPA1	-1.785	0.0000453						
125	201800_s_	OSBP	-1.574	0.000343						
126	207576_x_	OXT	-2.304	9.79E-11						
127	210401_at	P2RX1	-1.512	0.00000847						
128	200815_s_	PAFAH1B	-2.106	0.0000735						
129	213264_at	PCBP2	-1.588	0.000000212						
130	204491_at	PDE4D	-2.004	0.00000665						
131	219630_at	PDZK1IP1	-3.096	0.0000116						
132	205736_at	PGAM2	-1.728	0.000131						
133	218387_s_	PGLS	-1.742	0.000212						
134	210041_s_	PGM3	-2.562	2.24E-09						
135	206369_s_	PIK3CG	-8.185	1.99E-20						
136	202328_s_	PKD1	-5.46	0.000000414						
137	207717_s_	PKP2	-1.788	0.000306						
138	206311_s_	PLA2G1B	-1.645	0.00000423						
139	209597_s_	PNMA2	-1.543	0.0000317						
140	205909_at	POLE2	-2.094	0.00000162						
141	220632_s_	POMT2	-1.522	0.000338						
142	205478_at	PPP1R1A	-2.212	0.000042						
143	211169_s_	PPP1R3A	-1.55	0.0000197						
144	202186_x_	PPP2R5A	-2.255	0.0000295						
145	219515_at	PRDM10	-2.665	0.00000158						
146	209677_at	PRKCI	-1.643	0.0000426						
147	217786_at	PRMT5	-2.028	0.0000174						
148	203537_at	PRPSAP2	-3.139	2.02E-08						
149	218613_at	PSD3	-1.736	6.09E-09						
150	201532_at	PSMA3	-1.538	0.00000147						
151	208408_at	PTN	-2.056	0.0000108						
152	212013_at	PXDN	-1.669	0.00000556						
153	202990_at	PYGL	-2.461	4.55E-12						
154	212263_at	QKI	-3.499	0.00038						
155	213970_at	RABL3	-1.721	0.00000127						
156	213967_at	RALYL	-2.736	1.01E-11						
157	202483_s_	RANBP1	-1.882	0.00000175						
158	202362_at	RAP1A	-1.813	0.000000025						
159	204189_at	RARG	-2.532	0.00032						
160	206221_at	RASA3	-1.795	0.000264						
161	215089_s_	RBM10	-1.523	0.0000545						
162	201394_s_	RBM5	-1.689	2.49E-08						
163	212646_at	RFTN1	-1.548	0.00000772						
164	206321_at	RFX1	-1.539	0.000427						
165	214449_s_	RHOQ	-1.509	0.0000134						
166	201528_at	RPA1	-1.517	0.0000133						
167	202648_at	RPS19	-1.546	0.000000113						
168	215495_s_	SAMD4A	-1.572	0.00012						
169	210862_s_	SARDH	-1.751	0.0000654						
170	204166_at	SBNO2	-1.572	0.000172						
171	211733_x_	SCP2	-1.587	0.0000264						
172	218265_at	SECISBP2	-1.598	8.72E-08						
173	209719_x_	SERPINB3	-1.509	0.000363						
174	205933_at	SETBP1	-1.672	0.00000135						
175	214305_s_	SF3B1	-12.436	0.00000119						
176	213543_at	SGCD	-1.812	0.000000187						
177	212321_at	SGPL1	-1.502	0.0000143						
178	205367_at	SH2B2	-1.953	0.000107						
179	219083_at	SHQ1	-1.578	0.00000131						
180	204967_at	SHROOM2	-1.794	0.00000101						

181	204666_s_	SIKE1	-3.32	0.0000872						
182	222030_at	SIVA1	-1.622	0.000281						
183	205316_at	SLC15A2	-1.577	0.00000796						
184	220554_at	SLC22A7	-1.715	0.0000456						
185	213167_s_	SLC5A3	-5.084	0.0000102						
186	218317_x_	SLX1A/SL)	-1.544	0.000000133						
187	204240_s_	SMC2	-1.592	0.00000205						
188	212926_at	SMC5	-1.513	0.0000398						
189	205315_s_	SNTB2	-1.994	0.0000382						
190	210000_s_	SOCS1	-2.26	0.0000127						
191	209891_at	SPC25	-1.59	0.00000481						
192	205861_at	SPIB	-1.508	0.000461						
193	210693_at	SPPL2B	-2.011	1.48E-09						
194	204675_at	SRD5A1	-1.524	0.000000237						
195	212632_at	STX7	-1.92	0.00000108						
196	201263_at	TARS	-1.731	0.00000527						
197	204654_s_	TFAP2A	-1.661	3.39E-08						
198	205015_s_	TGFA	-14.044	1.01E-10						
199	203833_s_	TGOLN2	-2.179	0.000000782						
200	206409_at	TIAM1	-1.633	0.000341						
201	221496_s_	TOB2	-1.787	0.00000123						
202	215382_x_	TPSAB1/T	-1.813	0.0000765						
203	210882_s_	TRO	-1.619	0.00000209						
204	222244_s_	TUG1	-1.776	0.0000315						
205	206828_at	TXK	-2.547	0.0000331						
206	217497_at	TYMP	-1.573	0.00000179						
207	203721_s_	UTP18	-1.594	0.000281						
208	210322_x_	UTY	-1.768	6.38E-10						
209	201336_at	VAMP3	-1.703	0.0000259						
210	213480_at	VAMP4	-1.852	0.0000153						
211	205586_x_	VGF	-2.128	0.00000634						
212	212606_at	WDFY3	-1.501	0.000489						
213	212638_s_	WWP1	-2.142	0.000000961						
214	214567_s_	XCL2	-3.928	0.00000607						
215	203043_at	ZBED1	-1.575	0.00000772						
216	204216_s_	ZC3H14	-2.238	0.000000875						
217	33148_at	ZFR	-2.161	0.00000726						
218	213073_at	ZFYVE26	-3.081	0.00026						
219	212545_s_	ZHX3	-2.102	0.000185						
220	207117_at	ZNF117	-3.822	0.00000151						
221	219604_s_	ZNF3	-1.551	0.000014						
222	219741_x_	ZNF552	-4.853	1.51E-09						
